# Uncertainty Evaluation of Weibull Estimators through Monte Carlo Simulation: Applications for Crack Initiation Testing

**DOI:** 10.3390/ma9070521

**Published:** 2016-06-27

**Authors:** Jae Phil Park, Chi Bum Bahn

**Affiliations:** School of Mechanical Engineering, Pusan National University, Busan 46241, Korea; jppark@pusan.ac.kr

**Keywords:** crack initiation test, estimation uncertainty, Monte Carlo simulation, Weibull distribution

## Abstract

The typical experimental procedure for testing stress corrosion cracking initiation involves an interval-censored reliability test. Based on these test results, the parameters of a Weibull distribution, which is a widely accepted crack initiation model, can be estimated using maximum likelihood estimation or median rank regression. However, it is difficult to determine the appropriate number of test specimens and censoring intervals required to obtain sufficiently accurate Weibull estimators. In this study, we compare maximum likelihood estimation and median rank regression using a Monte Carlo simulation to examine the effects of the total number of specimens, test duration, censoring interval, and shape parameters of the true Weibull distribution on the estimator uncertainty. Finally, we provide the quantitative uncertainties of both Weibull estimators, compare them with the true Weibull parameters, and suggest proper experimental conditions for developing a probabilistic crack initiation model through crack initiation tests.

## 1. Introduction

Stress corrosion cracking (SCC) is one of the main material-related issues that occur in the operation of nuclear reactors [1,2,3]. Particularly, in pressurized water reactors, the occurrence of SCC at a reactor’s pressure boundary can cause a loss-of-coolant accident. Therefore, many researchers have endeavored to predict SCC initiation time for a given component. However, accurately predicting SCC initiation is difficult because the mechanism is quite complex and not yet clearly understood; instead, empirical SCC initiation models are generally considered [4,5,6].

However, most SCC experiments show significant variation in cracking time, even though all specimens are tested in the same experimental conditions (e.g., temperature and stress level). Therefore, the Weibull distribution [7], which considers the effect of time-dependent material degradation, is widely accepted as a probabilistic model for SCC initiation time [6,8,9]. Probabilistic models cannot offer an exact cracking time but can offer a cracking probability as a function of time for a given set of conditions. In this case, SCC initiation testing is required to determine the cracking probability function (i.e., the unreliability function).

The typical experimental procedure of an SCC initiation test involves an interval-censored reliability test. That is, stressed specimens are exposed to a corrosive environment and censored at scheduled periods. The results of these tests can be used to estimate the parameters of a Weibull distribution, using either maximum likelihood estimation (MLE) or median rank regression (MRR) [10].

Both estimation methods for Weibull parameters are anticipated to be more accurate with more test specimens and narrower censoring intervals. However, we do not yet know the optimal number of test specimens and censoring intervals required to estimate a sufficiently accurate Weibull distribution. In this study, we use Monte Carlo simulation to compare MLE and MRR estimators and quantify the effects of specimen number, test duration, and censoring interval on the uncertainty of the estimated Weibull parameters.

## 2. Weibull Estimation

### 2.1. Weibull Distribution

The cumulative distribution function (CDF) of a two-parameter Weibull distribution is frequently used as a cracking probability function, and is given by [10]:

(1) $$F\left(t;\beta ,\eta \right)=1-\exp\left[-{\left(\frac{t}{\eta }\right)}^{\beta }\right];\;t\geq 0;\;\beta ,\;\eta >0$$

where *t* is time, β is the shape parameter, and η is the scale parameter of the Weibull distribution.

If β < 1, the cracking rate, or hazard function, decreases with time. If β>1, the cracking rate increases monotonically. This indicates time-dependent material degradation, or aging effects. If β=1, the Weibull distribution becomes equivalent to an exponential distribution and the cracking rate is not influenced by time. The scale parameter η is also called characteristic time, which is the quantile at which the CDF of the Weibull distribution reaches approximately 0.632.

### 2.2. Median Rank Regression

MRR is a method that can derive a cracking probability function from the result of a crack initiation test. It is reasonable to assume that all specimens are tested independently; that is, the status of one specimen does not affect the cracking probability of the other specimens.

Let *N* be the total number of specimens and *j* be the number of cracked specimens. Then, the distribution of *j* at a certain time follows a binomial distribution. The CDF of the binomial distribution can be expressed as follows [11]:

(2) $$\begin{array}{cc} {\text{CDF}}_{\text{Bin}}\left(j;N,F\left(t\right)\right) & =\;\sum_{i=0}^{j}\left(\begin{array}{c} N\\ i \end{array}\right){\left[F\left(t\right)\right]}^{i}{\left[1-F\left(t\right)\right]}^{N-i}\\ & =\left(N-j\right)\left(\begin{array}{c} N\\ j \end{array}\right)\int_{0}^{1-F\left(t\right)}t^{N-j-1}{\left(1-t\right)}^{j}dt\\ & =I_{1-F\left(t\right)}\left(N-j,j+1\right) \end{array}$$

where F(t) is the cracking probability function and I is the regularized incomplete beta function. When $CDF_{Bin}$ is set to 0.5, the value of F(t) at a certain time can be calculated, and is called the median rank. If the total number of specimens is very large, the value of the median rank is close to the cracked fraction j/N.

**Figure 1:**
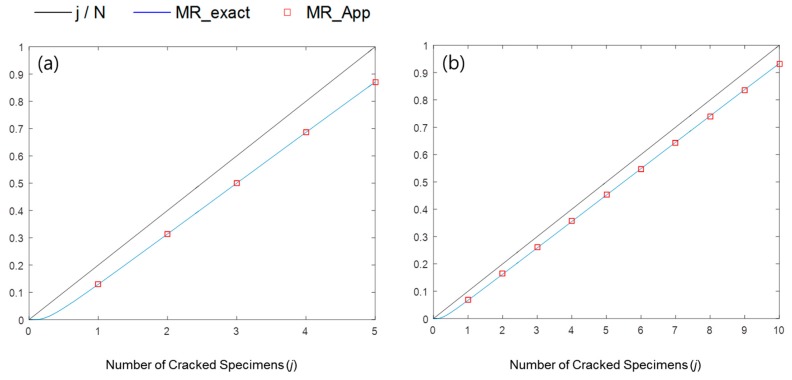
Comparison of exact median rank values (blue line) and their approximations (red squares) when: (**a**) *N* = 5; and (**b**) *N* = 10.

Benard and Bos-Levenbach [12] suggested a simple approximation for non-statisticians to easily calculate the median rank:

(3) $$\begin{array}{cc} F_{Med}\left(t\right) & =\;1-I_{0.5}^{-1}\left(N-j+1,\;j\right)\\ & \approx \;\frac{j\;-\;0.3}{N\;+\;0.4} \end{array}$$

where $F_{Med}\left(t\right)$ is the cracking probability function calculated using median rank. Figure 1 shows that the exact median rank values are very close to their approximations. Therefore, in this study, we use the approximation, defined in Equation (3), to improve calculation speed.

**Figure 2:**
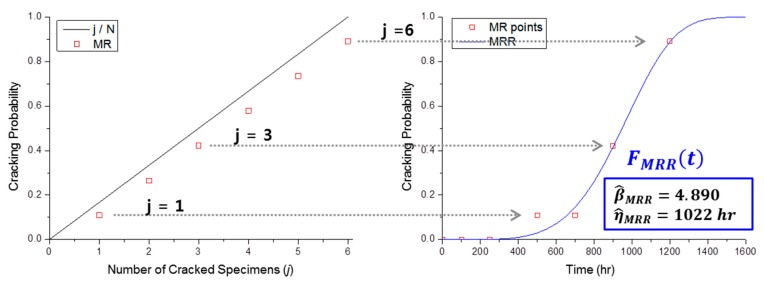
Example of Weibull estimation by MRR with the test data from Table 1.

If the test is not censored continuously (i.e., if it is an interval-censored test), the resulting $F_{\text{Med}}\left(t\right)$ must be treated as a set of unreliability points and not as a function. With this median rank point set, it is possible to estimate the Weibull distribution, which is the model of SCC initiation, through regression [10,13]. Figure 2 is an example of Weibull estimation using MRR that uses the test data in Table 1. The red dots show the median rank points, $F_{\text{MRR}}\left(t\right)$ is the estimated Weibull CDF by regression with the median rank points, and ${\hat{\beta }}_{MRR}$ and ${\hat{\eta }}_{MRR}$ are the Weibull shape and scale parameters, respectively, estimated by MRR.

A widely used Weibull regression technique employs the linearization of the Weibull distribution, which is as follows:

(4) $$\text{ln}\left[\text{ln}\left[\frac{1}{1-F\left(t\right)}\right]\right]=\beta \text{ln}\left(t\right)-\beta \text{ln}\left(\eta \right)$$

However, this Weibull estimation method encounters limitations. First, it cannot handle the case in which there are zero cracking points, which returns a negative infinity value in the linearized form. Second, the Weibull distribution is nonsymmetrical and the error in the rank probability estimation is not random in nature [14]. Weights in the MRR linear function are based on the incorrect assumption of uncorrelated, equal variance residuals [15]. Therefore, we used a nonlinear curve-fitting function, *lsqcurvefit*, provided by MATLAB, which is based on the least squares method.

### 2.3. Maximum Likelihood Estimation

The MLE method estimates the parameters of the Weibull distribution directly by using the likelihood function, instead of the cracking probability at each censoring point. The likelihood function for the interval-censored case is given by [13]:

(5) $$L\left(\beta ,\eta \right)=\;\overset{S}{\underset{i=1}{\prod }}\left[1-F\left(s_{i};\beta ,\eta \right)\right]\cdot \overset{C}{\underset{j=1}{\prod }}\left[F\left(c_{j_{U}};\beta ,\eta \right)-F\left(c_{j_{L}};\beta ,\eta \right)\right]$$

where *S* is the number of suspended specimens, $s_{i}$ is the last censoring time of *i*_th_ suspended specimen, *C* is the number of interval-censored cracked specimens, and $c_{j_{U}}$ and $c_{j_{L}}$ are the upper and lower bound times, respectively, of the censoring interval for the *j*_th_ cracking. The sum of *S* and *C* is equal to the total number of specimens *N*.

The use of log-likelihood is convenient to determine the Weibull parameters that maximize the likelihood function (i.e., $\arg\underset{\left(\beta ,\;\eta \right)}{\text{max}}\;L\left(\beta ,\eta \right)$). The log-likelihood function is as follows:

(6) $$\begin{array}{cc} \Lambda \left(\beta ,\eta \right) & =\ln L\left(\beta ,\eta \right)\\ & =\;\sum_{i=1}^{S}\ln\left[1-F\left(s_{i};\beta ,\eta \right)\right]+\;\sum_{j=1}^{C}\ln\left[F\left(c_{j_{U}};\beta ,\eta \right)-F\left(c_{j_{L}};\beta ,\eta \right)\right] \end{array}$$

The maximum likelihood point is obtained where both partial derivatives of Λ(β,η) reach zero. Therefore, the maximum likelihood point is given by:

(7) $$\{\begin{array}{c} \frac{\partial }{\partial \beta }\Lambda \left(\beta ,\eta \right)=0\\ \frac{\partial }{\partial \eta }\Lambda \left(\beta ,\eta \right)=0 \end{array}$$

Substituting Equations (1) and (6) into Equation (7), we can obtain the final simultaneous equation:

(8) $$\left\{ \begin{array}{c} \sum_{i=1}^{s}\left[-{\left(\frac{s_{i}}{\eta }\right)}^{\beta }\ln\left(\frac{s_{i}}{\eta }\right)\right]+\sum_{j=1}^{c}\left[\frac{-{\left(\frac{c_{jL}}{\eta }\right)}^{\beta }\ln\left(\frac{c_{jL}}{\eta }\right)\exp\left[-{\left(\frac{c_{jL}}{\eta }\right)}^{\beta }\right]+{\left(\frac{c_{jU}}{\eta }\right)}^{\beta }\ln\left(\frac{c_{jU}}{\eta }\right)\exp\left[-{\left(\frac{c_{jU}}{\eta }\right)}^{\beta }\right]}{\exp\left[-{\left(\frac{c_{jL}}{\eta }\right)}^{\beta }\right]-\exp\left[-{\left(\frac{c_{jU}}{\eta }\right)}^{\beta }\right]}\right]=0\\ \sum_{i=1}^{s}\left[\left(\frac{\beta }{\eta }\right){\left(\frac{s_{i}}{\eta }\right)}^{\beta }\right]+\sum_{j=1}^{c}\left[\left(\frac{\beta }{\eta }\right)\frac{{\left(\frac{c_{jL}}{\eta }\right)}^{\beta }\exp\left[-{\left(\frac{c_{jL}}{\eta }\right)}^{\beta }\right]-{\left(\frac{c_{jU}}{\eta }\right)}^{\beta }\exp\left[-{\left(\frac{c_{jU}}{\eta }\right)}^{\beta }\right]}{\exp\left[-{\left(\frac{c_{jL}}{\eta }\right)}^{\beta }\right]-\exp\left[-{\left(\frac{c_{jU}}{\eta }\right)}^{\beta }\right]}\right]=0 \end{array}\right.$$

The derivation of Equation (8) is available in the Supplementary Materials. It would be extremely difficult to determine a general analytical solution for Equation (8); therefore, we used a numerical approach. In this case, MATLAB offers the numerical nonlinear simultaneous equation function *fsolve*.

## 3. Monte Carlo Simulation

**Figure 3:**
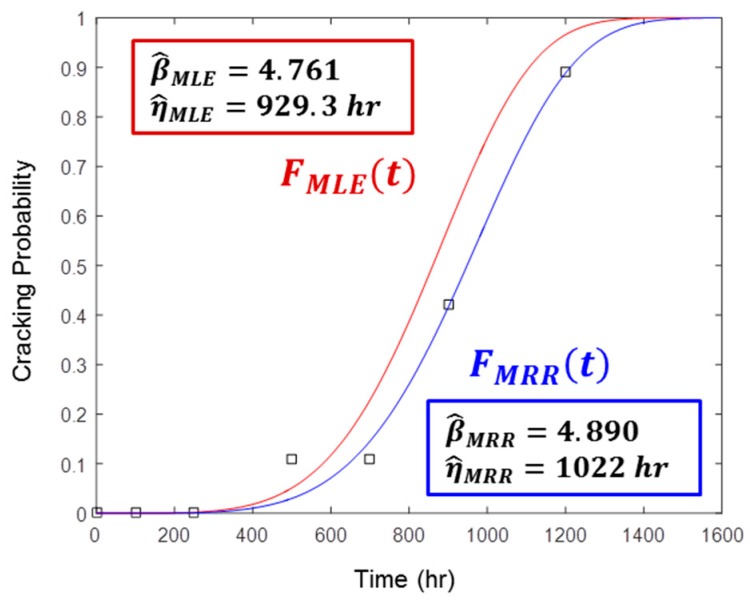
Comparison of different Weibull curves estimated by MRR or MLE from the test data in Table 1. The blue line is the Weibull curve estimated by MRR and the red line is that by MLE. The black squares are the median rank points.

The goals of MRR and MLE are the same: the estimation of Weibull parameters. However, the resulting estimators are slightly different, even though they were both deduced from the same test result. Figure 3 shows the different Weibull curves estimated from the same test data, found in Table 1. It is intriguing to know which estimation method generates more precise estimators. A Weibull distribution with precise estimators could better describe inherent SCC initiation behavior.

Theoretically, it is possible to calculate the estimation confidence for data containing the exact cracking time only; that is, when cracking is continuously monitored using a direct current potential drop technique. However, an MLE theory to set the estimation confidence for interval-censored data is not yet available [10]. Therefore, Monte Carlo simulation [16] could be used to quantitatively evaluate estimator uncertainties of MLE and MRR. The experimental factors considered in the simulation study are as follows.

- True Weibull parameters: It is assumed that the inherent cracking probability is Weibull-distributed. If the standardized estimation errors were affected by the value of the true scale parameter ($\eta_{true}$), only changing the time unit (e.g., hours to seconds) could affect standardized estimation errors. It is a contradiction. In fact, a scale parameter is just a scale factor. Therefore, standardized estimation errors are not affected by the value of $\eta_{\text{true}}$ [15]. Without loss of generality, $\eta_{true}$ can be fixed at 100, whereas the value of the true Weibull shape parameter ($\beta_{true}$) could affect the standardized estimation errors. To examine the degree of aging effects, several values of $\beta_{true}$ (2, 3, and 4) are examined. In earlier studies, the values of the Weibull shape parameter for crack initiation time range from 2 to ~4 [6,17,18,19].
- The number of specimens: The SCC initiation test for nuclear reactor materials requires a corrosive environment at high temperatures and pressures. Thus, simultaneously testing a large number of specimens is difficult. Therefore, the base number of test specimens is set at 10. To evaluate the effect of the number of specimens, additional cases were studied (see Table 2).
- Test duration: When planning the SCC test, cracking will not necessarily occur for every specimen within the available testing time. Thus, the test duration is also a factor affecting the uncertainty of Weibull estimators. For convenience, the baseline test duration is set at 120% of $\eta_{true}$. Additional test duration cases are shown in Table 2.
- Censoring interval: A shorter censoring interval may be better for developing an accurate SCC initiation model. However, frequent censoring would be inconvenient for the experimenters. Therefore, the baseline censoring interval is set at 20% of $\eta_{true}$. Other examined interval cases are shown in Table 2. Although time-dependent censoring intervals are more general for real cracking tests, it is assumed that censoring intervals do not vary with time. If we consider time-dependent censoring intervals, there are too many possible combinations of experimental conditions to perform a simulation study.

**Figure 4:**
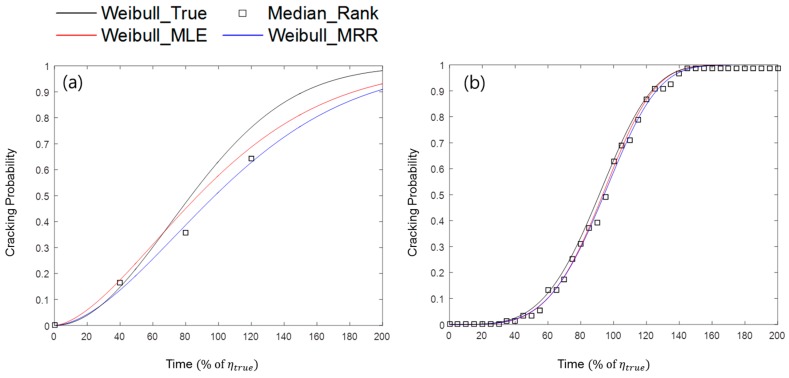
Two examples of simulation experiments with relatively (**a**) poor test condition; and (**b**) ideal test condition.

Figure 4 shows examples of the simulation experiments with different combinations of conditions. Weibull_True represents the pre-assumed true cracking probability, which follows a Weibull distribution. Median_Rank is the set of cumulative cracking point probabilities resulting from the randomly simulated cracking experiments and is calculated by the median rank method. Weibull_MLE and Weibull_MRR are the estimated Weibull distributions obtained from simulation experiments using MLE and MRR, respectively.

Figure 4a is an example of a simulation in which the number of specimens is relatively small, the censoring interval is wide, and the test duration is short. In this case, the estimated Weibull curves, Weibull_MLE and Weibull_MRR, are quite different from the true cracking probability curve, Weibull_True. Figure 4b shows another example of the simulation, in which the number of specimens is relatively large, the censoring interval is narrow, and the test duration is long. In this simulation, the estimated Weibull curves approximate the true Weibull curve, following our intuition. The detailed experimental conditions applied in Figure 4 are described in Table 3.

By combining the considered experimental conditions, a total of 441 simulation cases were studied. Each case was simulated 20,000 times.

We think that it is important to represent the degree of bias and degree of dispersion of estimators respectively in every specific experimental condition. For the experimenters who want to develop a cracking prediction model, both the degree of bias and degree of dispersion of estimators are necessary to guess their model uncertainty. For the same reason, the estimation uncertainties of *β* and *η* are respectively represented.

The 5th, 50th, and 95th percentiles of the Weibull estimators were derived from each simulation case. Further, these estimators were converted to the standard error, which is defined as follows:

(9) $$\text{SE}\left(\hat{\beta }\right)=\;\frac{\hat{\beta }-\beta_{true}}{\beta_{true}};\text{ SE}\left(\hat{\eta }\right)=\;\frac{\hat{\eta }-\eta_{true}}{\eta_{true}}$$

where $\hat{\beta }$ and $\hat{\eta }$ are the Weibull parameters estimated by MRR or MLE. To quantify the Weibull estimator deviations, we utilized a standardized length of 90% confidence interval, defined as follows:

(10) $${\text{SLCI}}_{90\%}\left(\hat{\beta }\right)=\text{SE}\left({\hat{\beta }}_{95\%}\right)\;-\text{SE}\left({\hat{\beta }}_{5\%}\right);\;{\text{SLCI}}_{90\%}\left(\hat{\eta }\right)=\text{SE}\left({\hat{\eta }}_{95\%}\right)\;-\text{SE}\left({\hat{\eta }}_{5\%}\right).$$

The true Weibull parameters ($\beta_{true},\eta_{true}$) are input as initial values of numerical solvers (i.e., the *fsolve* and *lsqcurvefit* functions in MATLAB). If a given combination of experimental conditions is too poor (e.g., cases with a small specimen number and wide censoring interval), it is possible to fail to find estimators with this numerical approach; in these cases, we exclude the failed estimators.

## 4. Results and Discussion

### 4.1. Fixed Test Duration

We fixed the test duration at 120% of $\eta_{true}$, the baseline case for test duration, to examine both the effects of the number of specimens and censoring interval.

**Figure 5:**
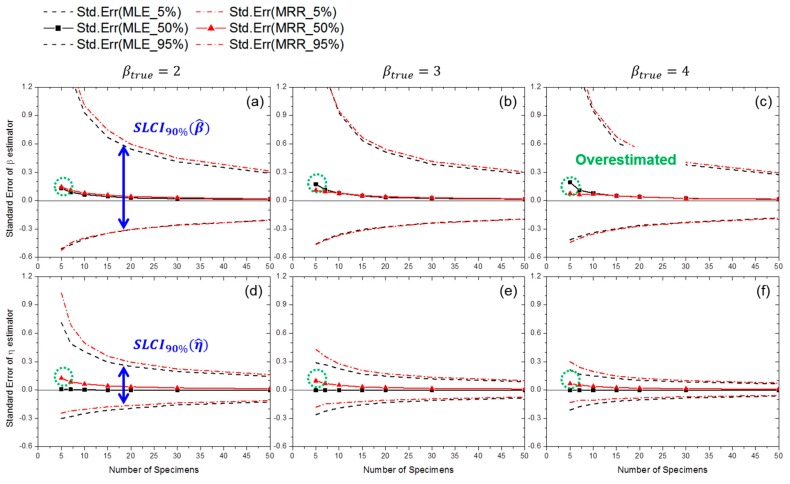
Effect of specimens number on $\text{SE}\left(\hat{\beta }\right)$ at: (**a**) $\beta_{true}=2$; (**b**) $\beta_{true}=3$; and (**c**) $\beta_{true}=4$; and on $\text{SE}\left(\hat{\eta }\right)$ at: (**d**) $\beta_{true}=2$; (**e**) $\beta_{true}=3$; and (**f**) $\beta_{true}=4$ (censoring interval: 20% of $\eta_{true}$; test duration: 120% of $\eta_{true}$).

As a special case, Figure 5 shows the effect of the number of specimens on estimation uncertainties when the censoring interval is fixed to 20% of $\eta_{true}$. When the number of specimens is large, there is a high probability of precise and accurate estimation with both MRR and MLE. For estimating the shape parameter β, MRR and MLE provide similar estimation uncertainty levels (see Figure 5a–c). It is likely that the shape parameters are overestimated with high probability when the number of specimens is less than 30 (i.e., ${\text{SE}}_{50\%}\left(\hat{\beta }\right)>0$). For the scale parameter η estimation, smaller deviation levels are observed in the estimation of scale parameter *η* for all ranges of specimen number as compared with those of the β estimators, especially at the high $\beta_{true}$ (see Figure 5d–f). Notably, the scale parameters estimated through MLE have a very slight bias in all ranges of specimen number (i.e., ${\text{SE}}_{50\%}\left({\hat{\eta }}_{\text{MLE}}\right)\approx 0$).

From these data, it is possible to calculate the confidence interval and bias of the estimators when real cracking test conditions are given. For example, if the test duration is 120% of $\eta_{true}$ and the censoring interval is 20% of $\eta_{true}$, the probability of obtaining $0.853<\frac{{\hat{\eta }}_{\text{MLE}}}{\eta_{true}}<1.151$ is approximately 90% and $\frac{{\hat{\eta }}_{\text{MLE},\;50\%}}{\eta_{true}}≅1$ with only 10 specimens when $\beta_{true}=4$ for the testing material (see Figure 5f).

**Figure 6:**
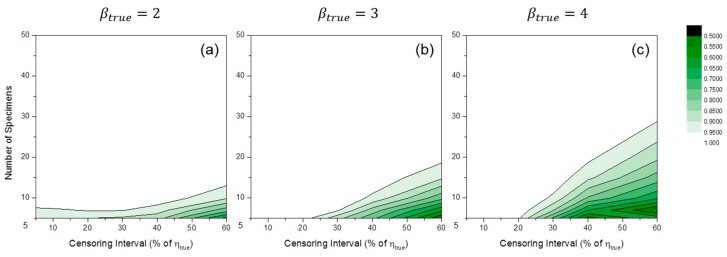
Convergence ratio distributions of MLE numerical estimation at: (**a**) $\beta_{true}=2$; (**b**) $\beta_{true}=3$; and (**c**) $\beta_{true}=4$ when test duration is 120% of $\eta_{true}$.

Figure 6 shows the convergence ratio distributions of MLE numerical estimation. The convergence ratio is defined as follows:

(11) $$\text{Convergence Ratio}=\frac{\text{Number of converged estimations by numerical solver}}{\text{Number of total simulation}(=20,000)}$$

The convergence ratio decreased when the number of specimens was small and the censoring interval was wide. This trend was enlarged when the value of $\beta_{true}$ increased. If the convergence ratio were too low, there would be a filtering effect caused by the disregard of outlier estimators. That is, for low convergence ratio region, output estimators were not purely random. It is recommended to be careful when analyzing the results in this region.

Although it is known that MLE convergence ratios might be improved by restricting *β* > 1 [20], we did not use this algorithm in this study. It will be considered in later research. For MRR estimation, the convergence ratios were mostly close to unity in all simulation cases.

**Figure 7:**
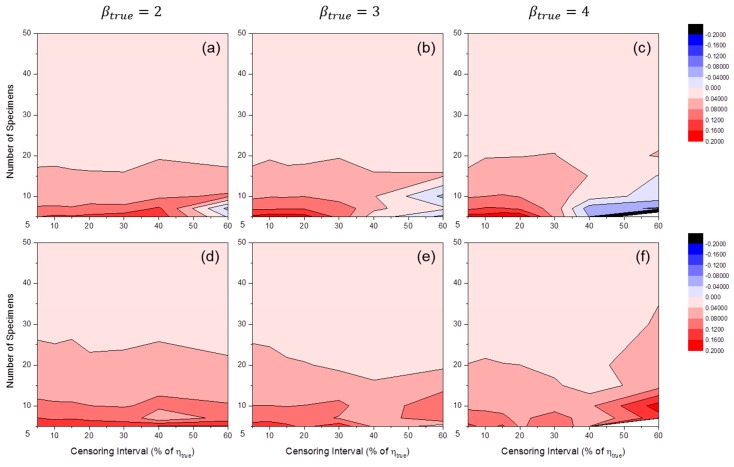
Distributions of ${\text{SE}}_{50\%}\left({\hat{\beta }}_{MLE}\right)$ at: (**a**) $\beta_{true}=2$; (**b**) $\beta_{true}=3$; and (**c**) $\beta_{true}=4$; and ${\text{SE}}_{50\%}\left({\hat{\beta }}_{MRR}\right)$ at: (**d**) $\beta_{true}=2$; (**e**) $\beta_{true}=3$; and (**f**) $\beta_{true}=4$ (test duration: 120% of $\eta_{true}$).

Figure 7 shows the distributions of ${\text{SE}}_{50\%}\left(\hat{\beta }\right)$ by MLE or MRR. These results indicate a bias in Weibull shape parameter estimation. It is likely that when the number of specimens is relatively small, $\beta_{\text{true}}$ tends to be overestimated, as in Figure 5. However, this trend did not occur when MLE was used and the censoring interval was relatively wide. Furthermore, if the value of $\beta_{\text{true}}$ was relatively large, underestimation occurred in wide censoring interval regions of MLE estimators (see Figure 7c).

**Figure 8:**
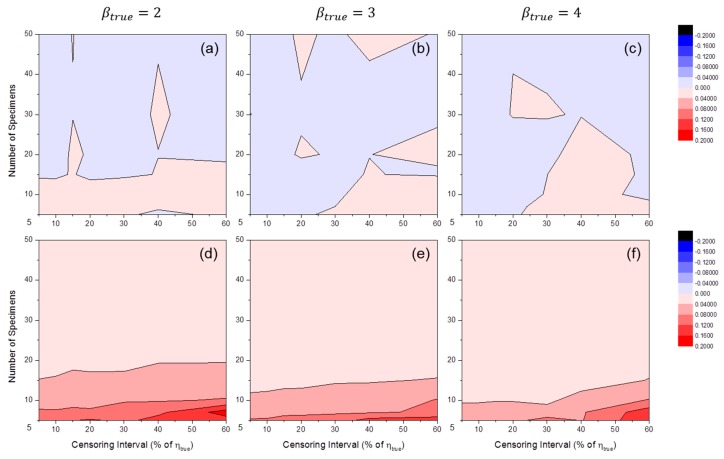
Distributions of ${\text{SE}}_{50\%}\left({\hat{\eta }}_{MLE}\right)$ at: (**a**) $\beta_{true}=2$; (**b**) $\beta_{true}=3$; and (**c**) $\beta_{true}=4$; and ${\text{SE}}_{50\%}\left({\hat{\eta }}_{MRR}\right)$ at: (**d**) $\beta_{true}=2$; (**e**) $\beta_{true}=3$; and (**f**) $\beta_{true}=4$ (test duration: 120% of $\eta_{true}$).

Figure 8 shows the distributions of ${\text{SE}}_{50\%}\left(\hat{\eta }\right)$ by MLE or MRR. These results indicate bias in Weibull scale parameter estimation. It is interesting that when MLE was used, very little bias was observed in all simulation cases. For MRR, a tendency toward overestimation (i.e., ${\text{SE}}_{50\%}\left(\hat{\eta }\right)>0$) occurred when the number of specimens was relatively small. This tendency was slightly amplified when $\beta_{true}$ was relatively small.

**Figure 9:**
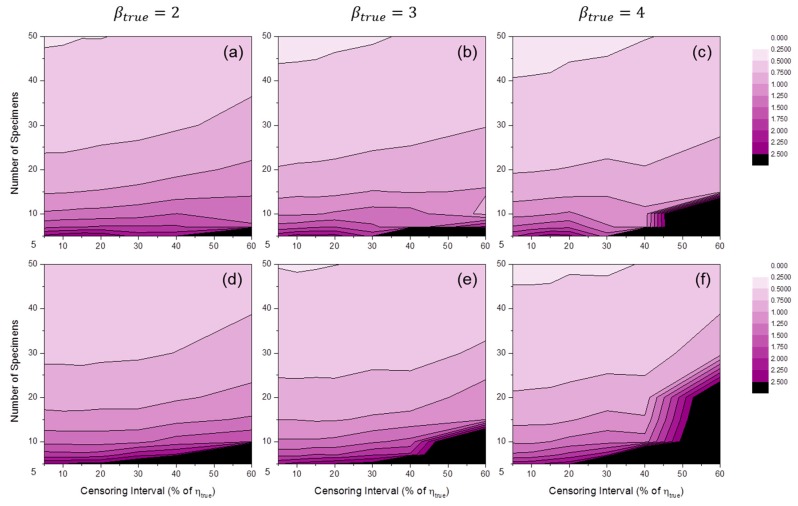
Distributions of ${\text{SLCI}}_{90\%}\left({\hat{\beta }}_{MLE}\right)$ at: (**a**) $\beta_{true}=2$; (**b**) $\beta_{true}=3$; and (**c**) $\beta_{true}=4$; and ${\text{SLCI}}_{90\%}\left({\hat{\beta }}_{MRR}\right)$ at: (**d**) $\beta_{true}=2$; (**e**) $\beta_{true}=3$; and (**f**) $\beta_{true}=4$ (test duration: 120% of $\eta_{true}$).

Figure 9 shows the distributions of ${\text{SLCI}}_{90\%}\left(\hat{\beta }\right)$ by MLE or MRR. These results illustrate the variance in Weibull shape parameter estimators. As anticipated, the variance in $\hat{\beta }$ was large when the number of specimens was relatively small and the censoring interval was wide. It is likely that there are critical lines after which estimators whose variances are too large are produced. Near the critical lines, the gradients of ${\text{SLCI}}_{90\%}\left(\hat{\beta }\right)$ were very high. Experimenters who want to develop cracking prediction models with a cracking test should avoid this region.

**Figure 10:**
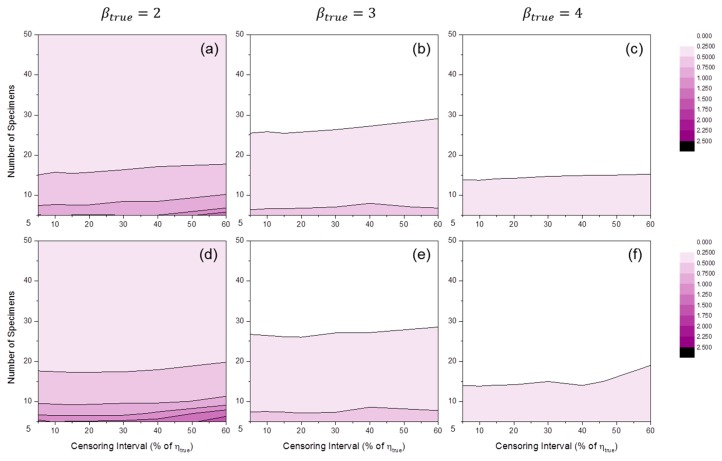
Distributions of ${\text{SLCI}}_{90\%}\left({\hat{\eta }}_{MLE}\right)$ at: (**a**) $\beta_{true}=2$; (**b**) $\beta_{true}=3$; and (**c**) $\beta_{true}=4$; and ${\text{SLCI}}_{90\%}\left({\hat{\eta }}_{MRR}\right)$ at: (**d**) $\beta_{true}=2$; (**e**) $\beta_{true}=3$; and (**f**) $\beta_{true}=4$ (test duration: 120% of $\eta_{true}$).

Figure 10 shows the distributions of ${\text{SLCI}}_{90\%}\left(\hat{\eta }\right)$ by MLE and MRR. These results show the variance in Weibull scale parameter estimators. The overall values of the ${\text{SLCI}}_{90\%}\left(\hat{\eta }\right)$ distributions were much lower than those of the ${\text{SLCI}}_{90\%}\left(\hat{\beta }\right)$ distributions, especially for the case of high $\beta_{true}$ values. Interestingly, shortening the censoring interval slightly affects the reduction of estimator deviations as compared to the case of ${\text{SLCI}}_{90\%}\left(\hat{\beta }\right)$, and there is no critical line for ${\text{SLCI}}_{90\%}\left(\hat{\eta }\right)$ distributions.

In fact, the distributions of Weibull estimators were not normal in most simulation conditions. Therefore, the upper and lower bound of the confidence intervals (e.g., ${\text{SE}}_{5\%}$ and ${\text{SE}}_{95\%}$, respectively) must be represented. We provide these data in the Supplementary Materials.

### 4.2. Fixed Censoring Interval

**Figure 11:**
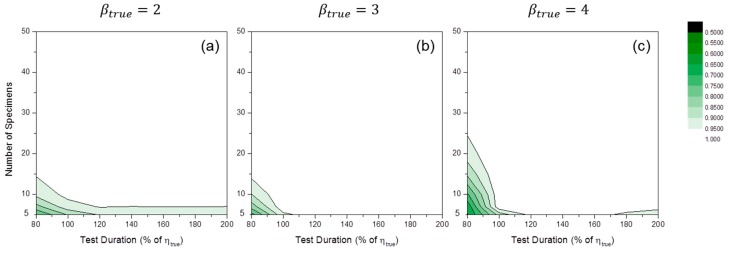
Convergence ratio distributions of MLE numerical estimation at: (**a**) $\beta_{true}=2$; (**b**) $\beta_{true}=3$; and (**c**) $\beta_{true}=4$ (censoring interval: 20% of $\eta_{true}$).

We fixed the censoring interval at 20% of $\eta_{true}$, the baseline case for the censoring interval, to examine the effects of both the number of specimens and test duration. Figure 11 shows the convergence ratio distributions of MLE numerical estimation. The convergence ratio decreased when the number of specimens was small, and the test duration short. This tendency was enlarged when the value of $\beta_{true}$ was increased. As previously mentioned, if the convergence ratios were too low, there would be the filtering effect. For MRR estimation, the convergence ratios were mostly close to unity in all simulation cases.

**Figure 12:**
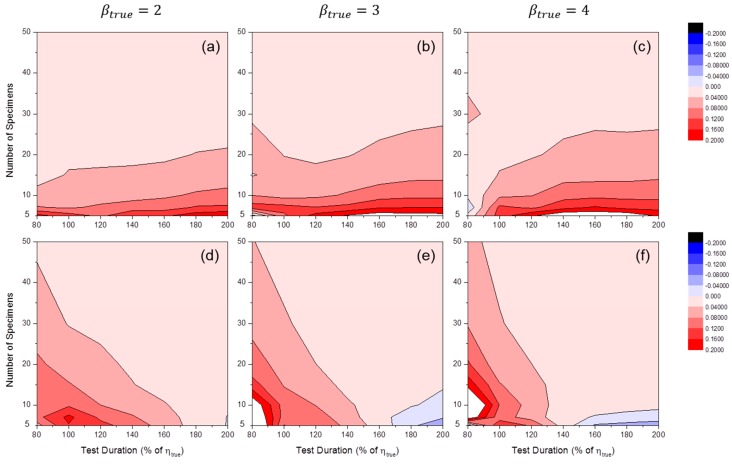
Distributions of ${\text{SE}}_{50\%}\left({\hat{\beta }}_{MLE}\right)$ at: (**a**) $\beta_{true}=2$; (**b**) $\beta_{true}=3$; and (**c**) $\beta_{true}=4$ and ${\text{SE}}_{50\%}\left({\hat{\beta }}_{MRR}\right)$ at: (**d**) $\beta_{true}=2$; (**e**) $\beta_{true}=3$; and (**f**) $\beta_{true}=4$ (censoring interval: 20% of $\eta_{true}$).

Figure 12 shows the distributions of ${\text{SE}}_{50\%}\left(\hat{\beta }\right)$. For MLE, when the number of specimens was relatively small, there was likely a tendency toward overestimation (i.e., $\text{SE}\left(\hat{\beta }\right)>0$). For MRR, overestimation was shown at short test durations and underestimation was shown at long test durations.

**Figure 13:**
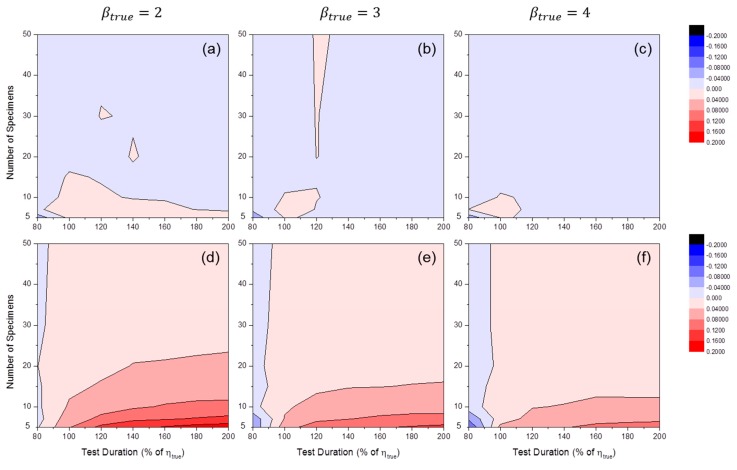
Distributions of ${\text{SE}}_{50\%}\left({\hat{\eta }}_{MLE}\right)$ at: (**a**) $\beta_{true}=2$; (**b**) $\beta_{true}=3$; and (**c**) $\beta_{true}=4$; and ${\text{SE}}_{50\%}\left({\hat{\eta }}_{MRR}\right)$ at: (**d**) $\beta_{true}=2$; (**e**) $\beta_{true}=3$; and (**f**) $\beta_{true}=4$ (censoring interval: 20% of $\eta_{true}$).

Figure 13 shows the distributions of ${\text{SE}}_{50\%}\left(\hat{\eta }\right)$. When MLE was used, very little bias was observed in all simulation ranges, as in the fixed test duration case (see Figure 8a–c). For MRR, overestimation (i.e., $\text{SE}\left(\hat{\eta }\right)>0$) occurred when the number of specimens was relatively small, except in cases of short test duration. This tendency was amplified when $\beta_{true}$ was relatively small.

**Figure 14:**
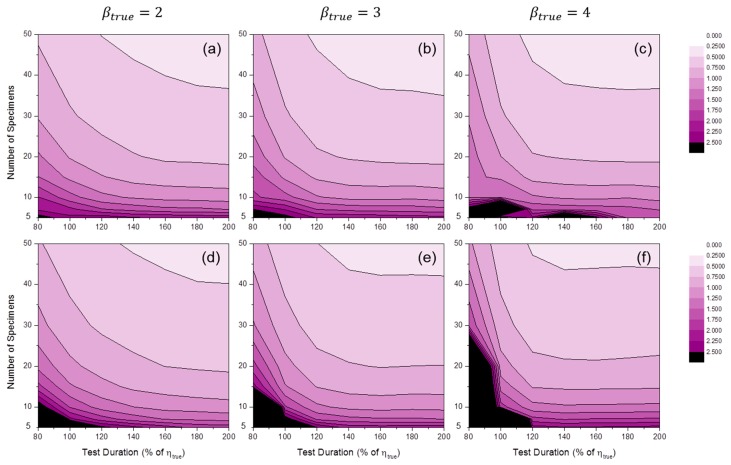
Distributions of ${\text{SLCI}}_{90\%}\left({\hat{\beta }}_{MLE}\right)$ at: (**a**) $\beta_{true}=2$; (**b**) $\beta_{true}=3$; (**c**) $\beta_{true}=4$ and ${\text{SLCI}}_{90\%}\left({\hat{\beta }}_{MRR}\right)$ at: (**d**) $\beta_{true}=2$; (**e**) $\beta_{true}=3$; (**f**) $\beta_{true}=4$ (censoring interval: 20% of $\eta_{true}$).

Figure 14 shows the distributions of ${\text{SLCI}}_{90\%}\left(\hat{\beta }\right)$. As anticipated, there was quite large variance in $\hat{\beta }$ when the number of specimens was relatively small and the test duration was short. It is likely that very long test durations are not useful for reducing estimator variance. This phenomenon is natural because censoring beyond a certain time only returned repeated meaningless results (i.e., all the specimens were cracked after this time). As in the fixed test duration case (see Figure 9), critical lines are observed in the distributions of ${\text{SLCI}}_{90\%}\left(\hat{\beta }\right)$. . The areas after critical line region increased when the value of $\beta_{\text{true}}$ was relatively high.

**Figure 15:**
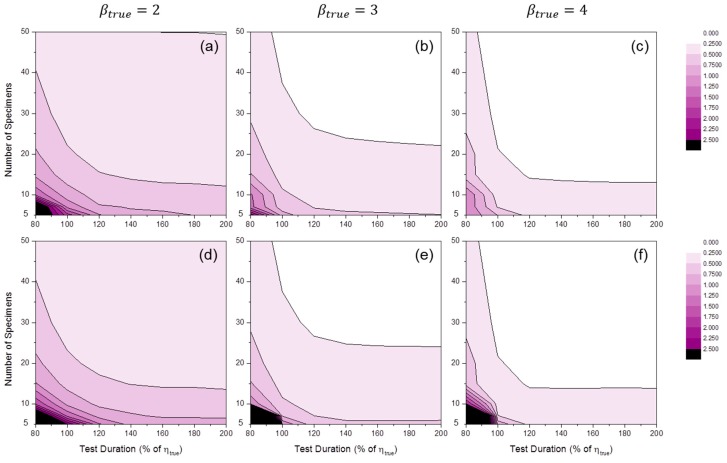
Distributions of ${\text{SLCI}}_{90\%}\left({\hat{\eta }}_{MLE}\right)$ at: (**a**) $\beta_{true}=2$; (**b**) $\beta_{true}=3$; and (**c**) $\beta_{true}=4$; and ${\text{SLCI}}_{90\%}\left({\hat{\eta }}_{MRR}\right)$ at: (**d**) $\beta_{true}=2$; (**e**) $\beta_{true}=3$; and (**f**) $\beta_{true}=4$ (censoring interval: 20% of $\eta_{true}$).

Figure 15 shows the distributions of ${\text{SLCI}}_{90\%}\left(\hat{\eta }\right)$. The overall values of the ${\text{SLCI}}_{90\%}\left(\hat{\eta }\right)$ distributions were much lower than those of the ${\text{SLCI}}_{90\%}\left(\hat{\beta }\right)$ distributions especially at high $\beta_{true}$. As in the case of ${\text{SLCI}}_{90\%}\left(\hat{\beta }\right)$, too long a test duration was not useful for reducing estimator variance. Contrary to the fixed test duration case (see Figure 10), there were critical lines in ${\text{SLCI}}_{90\%}\left(\hat{\eta }\right)$ distributions.

The upper and lower bounds for this fixed censoring interval case are also represented in the Supplementary Materials.

### 4.3. Fixed Number of Specimen

**Figure 16:**
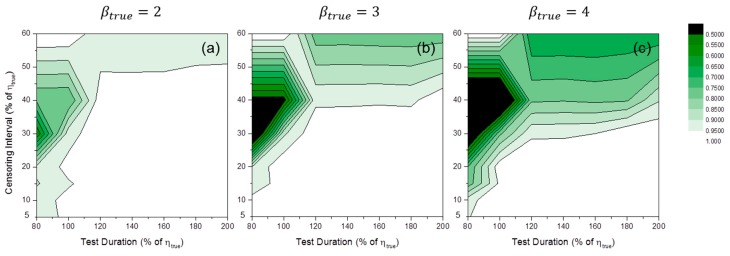
Convergence ratio distributions of MLE numerical estimation at: (**a**) $\beta_{true}=2$; (**b**) $\beta_{true}=3$; and (**c**) $\beta_{true}=4$ (number of specimens: 10).

**Figure 17:**
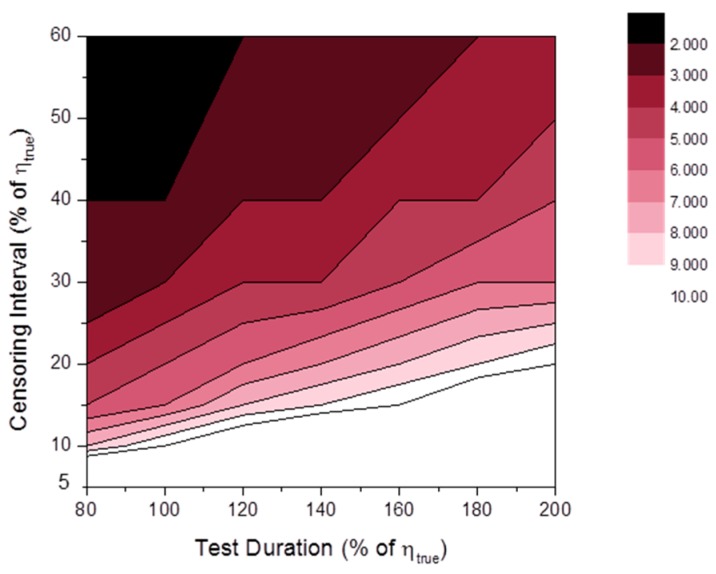
Distribution of the number of censoring times during the test duration.

We fixed the number of specimen at 10, the baseline case for the number of specimens, to examine both the effects of censoring interval and test duration. Figure 16 shows the convergence ratio distributions of MLE numerical estimation. It was quite complicated to find a general tendency from these results. We hypothesize this complexity is due to the complex distribution of the number of censoring times (see Figure 17). For example, if only one censoring was implemented during the simulation, the convergence ratio reaches unity even though the experimental condition of the simulation was very poor. For MRR estimation, the convergence ratios were mostly close to unity in all simulation cases.

In this case, with a fixed number of specimens, the distributions of ${\text{SE}}_{50\%}$ and ${\text{SLCI}}_{90\%}$ were very complex and it was difficult to find general tendencies for both the MLE and MRR cases. Therefore, we do not represent these results in this manuscript, but in the Supplementary Materials instead.

In fact, we think that the end cracking fraction would be a more appropriate factor of estimation uncertainty than test duration. First, end cracking fraction of the test is not directly related to the number of censoring times when the censoring interval is pre-determined. Second, it does not produce repeated meaningless results after a certain time (i.e., test will end when all specimens are cracked if end cracking fraction is set to unity). We will study the effects of end cracking fraction on Weibull estimation uncertainties in later research.

## 5. Conclusions

The main goal of this study is to suggest proper experimental conditions for experimenters who want to develop a probabilistic SCC initiation model through cracking tests. We consider the widely used MRR and MLE methods for Weibull estimation. By using Monte Carlo simulation, MRR and MLE estimator uncertainties were quantified in various experimental conditions. The following conclusions can be drawn:

- It is possible to calculate the confidence interval and bias of estimators when the real cracking test conditions are given.
- Very little bias is observed in all simulation ranges when MLE is used to estimate the scale parameter η.
- The overall deviations of $\hat{\eta }$ are much lower than those of $\hat{\beta }$ in the simulation study range. This effect is enlarged when the value of $\beta_{true}$ is relatively high. Therefore, it is not recommended to estimate β from a cracking test when the experimental conditions are poor.
- It is likely that there are critical lines after which estimators whose variances are too large are produced. Near the critical lines, the gradients of ${\text{SLCI}}_{90\%}$ are very high. It is recommended that experimenters avoid this region.
- Before the critical line region, too narrow censoring interval, or too long test duration, is not useful for reducing the estimation uncertainty.

## 6. Outlook

The following issues will be considered in the later research:

- In this study, it is assumed that censoring interval is time-independent variable. However, time-dependent censoring interval is more general for a real SCC test.
- The end cracking fraction seems more appropriate than the test duration for use as a factor of estimation uncertainty.
- To improve the convergence ratio of MLE, we will consider the numerical algorithm which restricts *β* > 1.
- If a cost function (e.g., specimen cost and labor cost) is obtained for an experiment, it will be possible to find out an optimum experimental condition which returns minimum estimation uncertainty with a given cost.

## Figures and Tables

**Table 1:** Example of a cracking initiation test result with six specimens.

Censoring Time (h)	Cracked Fraction	Median Rank
100	0/6	0
250	0/6	0
500	1/6	0.1091
700	1/6	0.1091
900	3/6	0.4214
1200	6/6	0.8909

**Table 2:** Experimental conditions for the Monte Carlo simulation.

True Weibull Parameter		Number of Specimens	Test Duration (% of $\eta_{true}$)	Censoring Interval (% of $\eta_{true}$)
$\eta_{true}$ (Dimensionless Time)	$\beta_{true}$			
100	2	5	80	5
	3	7	100	10
	4	10 *	120 *	15
		15	140	20 *
		20	160	30
		30	180	40
		50	200	60

* Baseline case of the simulation study.

**Table 3:** Combinations of experimental factors applied to examples in Figure 4.

Factors	Figure 4a	Figure 4b
$\eta_{true}$ (dimensionless time)	100	100
$\beta_{true}$	2	4
The number of specimen	10	50
Test duration (% of $\eta_{true}$)	120	200
Censoring interval (% of $\eta_{true}$)	40	5

## Supplementary Materials

The following are available online at www.mdpi.com/1996-1944/9/7/521/s1. Derivation of interval censored ML simultaneous equation for 2-parameter Weibull distribution (word file); Raw data of the simulation results for “fixed test duration”, “fixed censoring interval”, and “fixed specimen number” cases (excel files); All contour plots of the simulation results, which contains the upper bounds (i.e., ${\text{SE}}_{95\%}$), lower bounds (i.e., ${\text{SE}}_{5\%}$), and the contour plots for the fixed specimen number case (pdf file).

## References

[1] Lunceford W., DeWees T., Scott P. (2013). EPRI Materials Degradation Matrix, Rev. 3.

[2] Scott P., Meunier M.-C. (2007). Materials Reliability Program: Review of Stress Corrosion Cracking of Alloys 182 and 82 in PWR Primary Water Service (MRP-220).

[3] Kim K.J., Do E.S. (2015). Technical Report: Inspection of Bottom Mounted Instrumentation Nozzle.

[4] Amzallag C., Hong S.L., Pages C., Gelpi A. Stress corrosion life assessment of Alloy 600 PWR components. Proceedings of the 9th International Symposium on Environmental Degradation of Materials in Nuclear Power Systems, Water Reactors 1999.

[5] Garud Y.S. (2009). Stress Corrosion Cracking Initiation Model for Stainless Steel and Nickel Alloys.

[6] Erickson M., Ammirato F., Brust B., Dedhia D., Focht E., Kirk M., Lange C., Olsen R., Scott P., Shim D. (2011). Models and Inputs Selected for Use in the xLPR Pilot Study.

[7] Weibull W. (1939). A Statistical Theory of the Strength of Materials.

[8] Eason E. (2005). Materials Reliability Program: Effects of Hydrogen, pH, Lithium and Boron on Primary Water Stress Corrosion Crack Initiation in Alloy 600 for Temperatures in the Range 320–330 °C (MRP-147).

[9] Hwang I.S., Kwon S.U., Kim J.H., Lee S.G. (2001). An intraspecimen method for the statistical characterization of stress corrosion crack initiation behavior. Corrosion.

[10] McCool J. (2012). Using the Weibull Distribution: Reliability, Modeling, and Inference.

[11] Wadsworth G.P., Bryan J.G. (1960). Introduction to Probability and Random Variables.

[12] Benard A., Bos-Levenbach E.C. (1953). The plotting of observations on probability paper. Stat. Neerl..

[13] ReliaSoft Corporation (2014). Life Data Analysis Reference Book (E-Book).

[14] Merkle J.G., Wallin K., McCabe D.E. (1998). Technical Basis for an ASTM Standard on Determining the Reference Temperature, T0, for Ferritic Steels in the Transition Range.

[15] Genschel U., Meeker W.Q. (2010). A comparison of maximum likelihood and median-rank regression for Weibull estimation. Qual. Eng..

[16] Ross S.M. (2009). Introduction to Probability and Statistics for Engineers and Scientists.

[17] Hong J.D., Jang C., Kim T.S. (2012). PFM application for the PWSCC integrity of Ni-base alloy welds—Development and application of PINEP-PWSCC. Nucl. Eng. Technol..

[18] Dozaki K., Akutagawa D., Nagata N., Takihuchi H., Norring K. (2010). Effects of dissolved hydrogen condtent in PWR primary water on PWSCC initiation property. EJAM.

[19] Garud Y.S. SCC initiation model and its implementation for probabilistic assessment. Proceedings of the ASME Pressure Vessels & Piping Division.

[20] Pradhan B., Kundu D. (2014). Analysis of interval-censored data with Weibull lifetime distribution. Sankhya B.

