# Supplementary material for: Uncertainty Evaluation of Weibull Estimators through Monte Carlo Simulation: Applications for Crack Initiation Testing
Source: Materials (Basel). 2016 Jun 27;9(7):521. doi: 10.3390/ma9070521 (PMC5456946; doi:10.3390/ma9070521)

Contents

- Fixed Test Duration (120%)
- Fixed Censoring Interval (20%)
- Fixed Specimen Number (10 ea.)

Fixed Test Duration (120%)

- $\beta_{true} = 2$
  - ✓ Convergence ratio

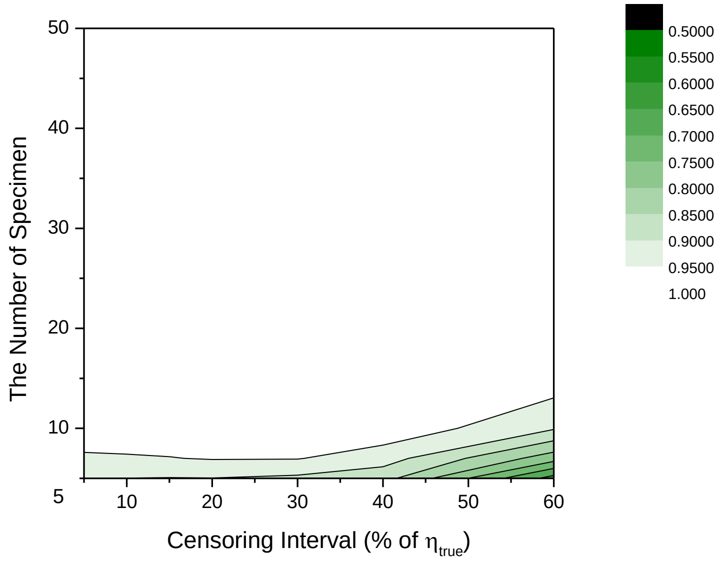

Fixed Test Duration (120%)

- $\beta_{true} = 3$
  - ✓ Convergence ratio

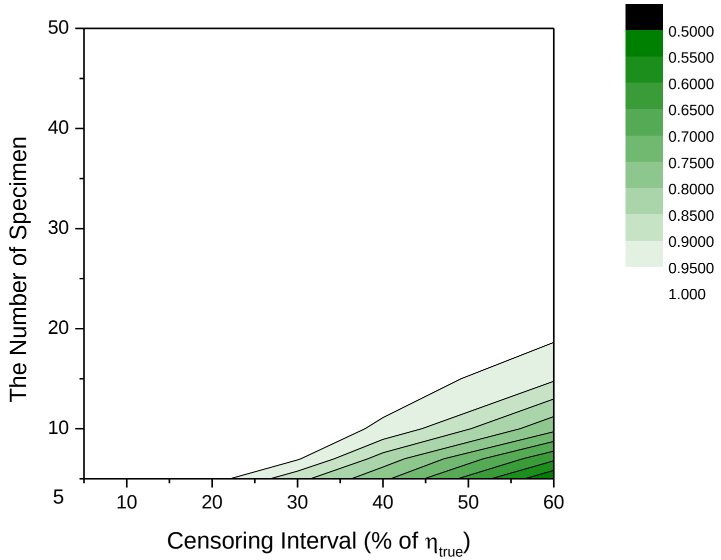

Fixed Test Duration (120%)

- $\beta_{true} = 4$
  - ✓ Convergence ratio

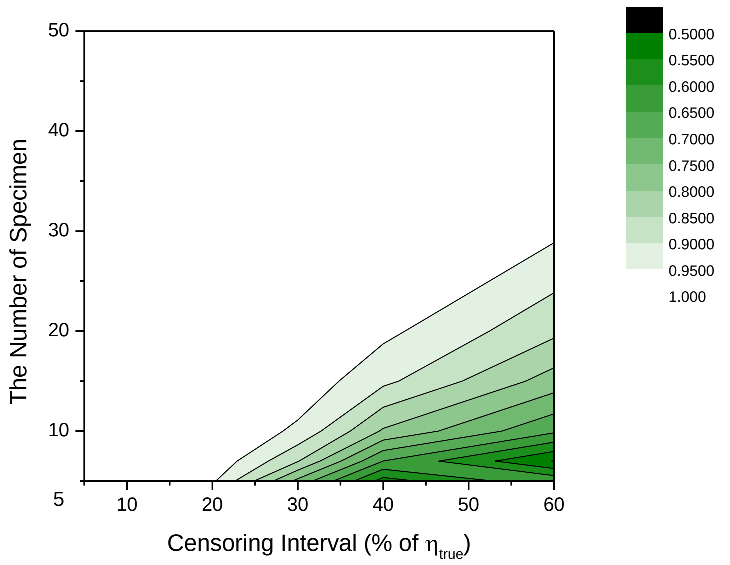

Fixed Test Duration (120%)

- $\beta_{true} = 2$

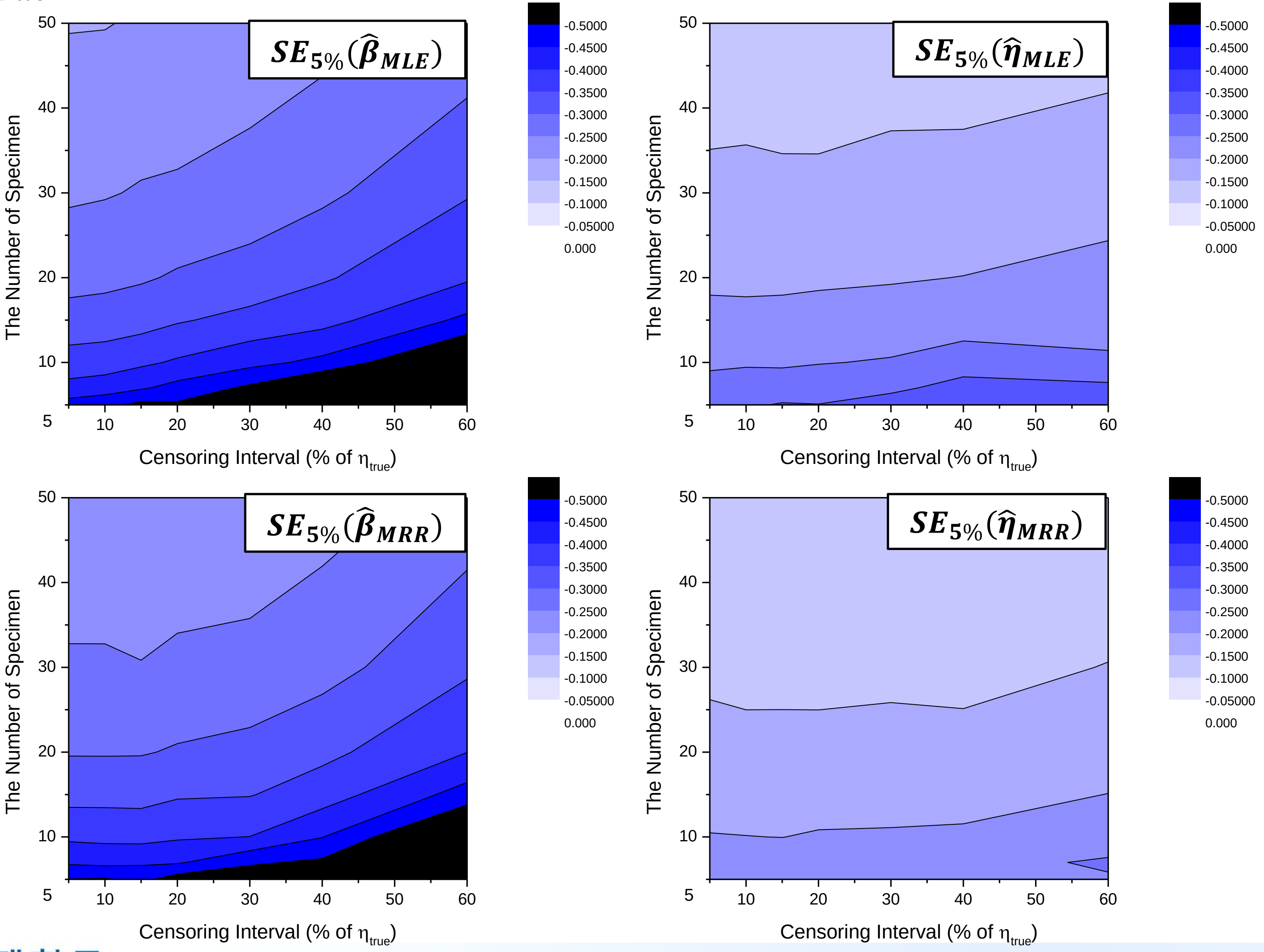

Fixed Test Duration (120%)

- $\beta_{true} = 3$

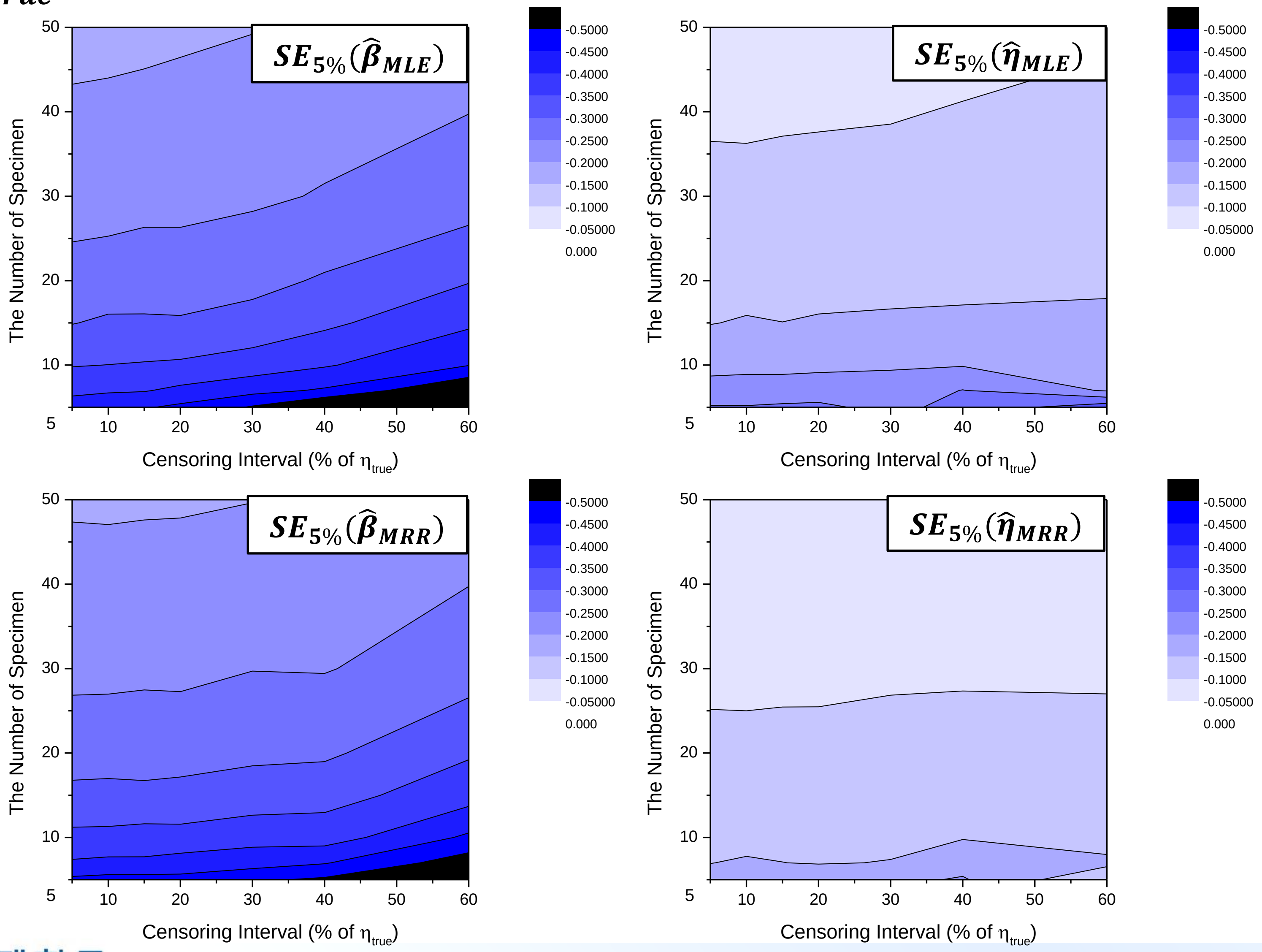

Fixed Test Duration (120%)

- $\beta_{true} = 4$

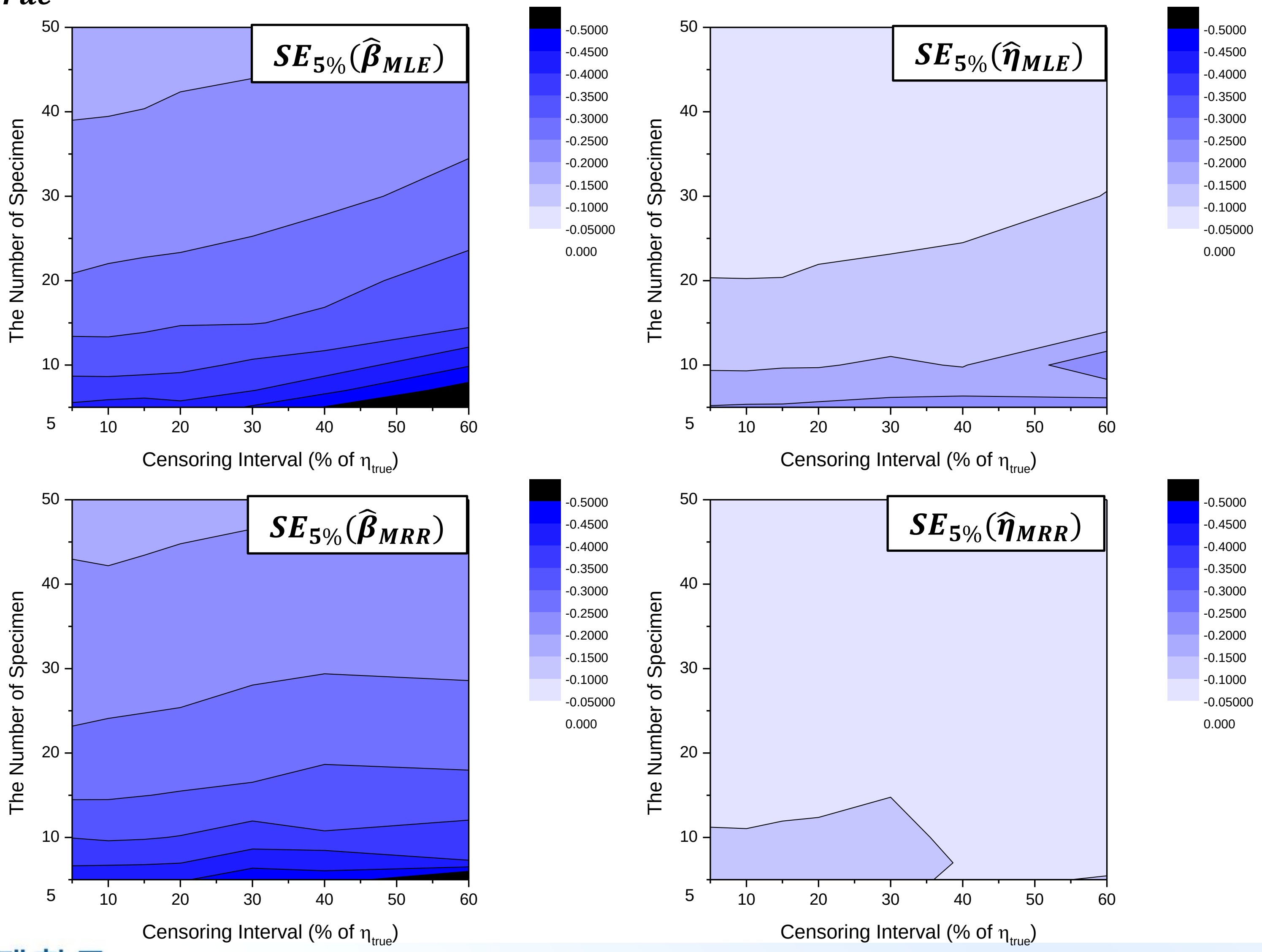

Fixed Test Duration (120%)

- $\beta_{true} = 2$

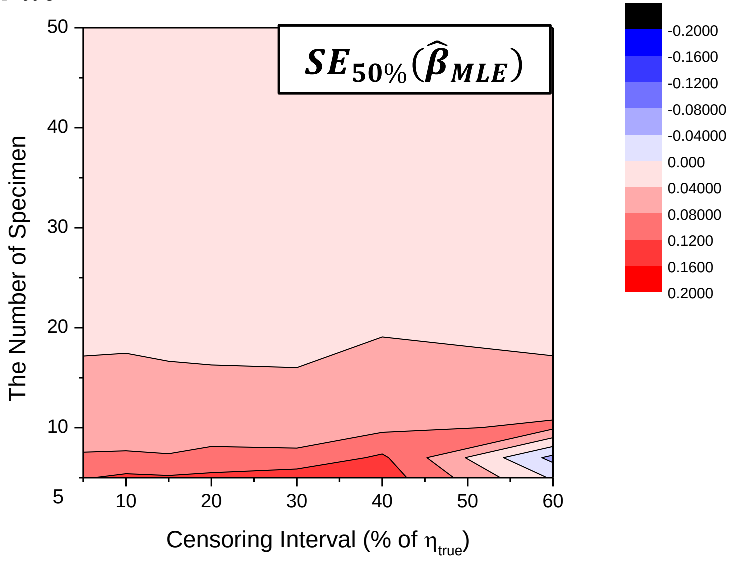

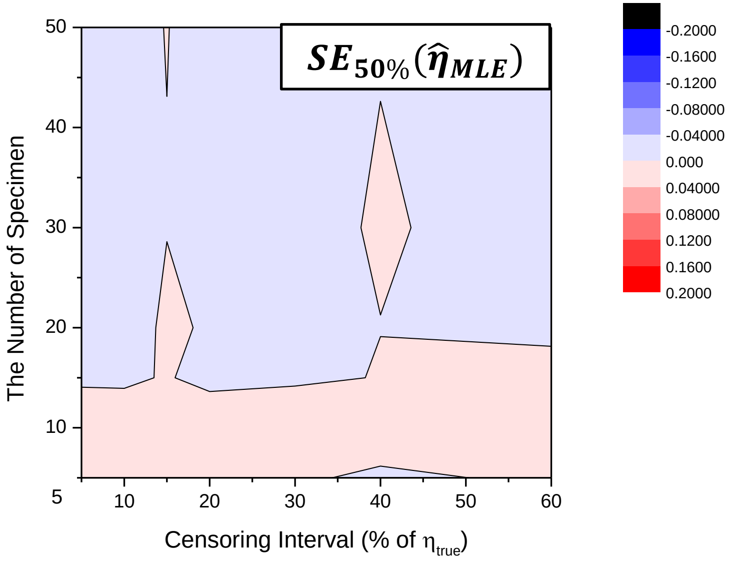

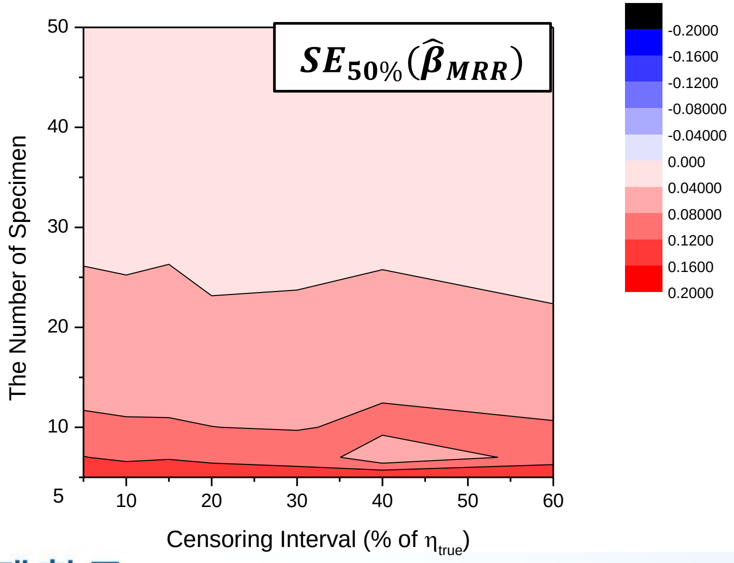

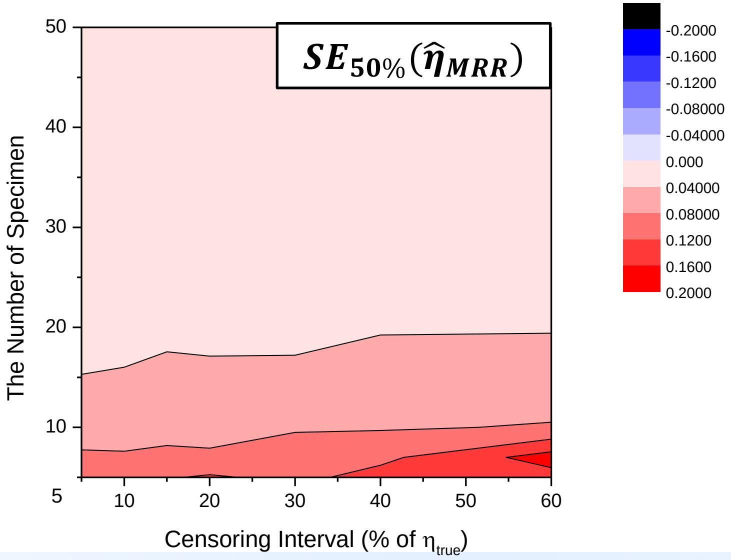

Fixed Test Duration (120%)

- $\beta_{true} = 3$

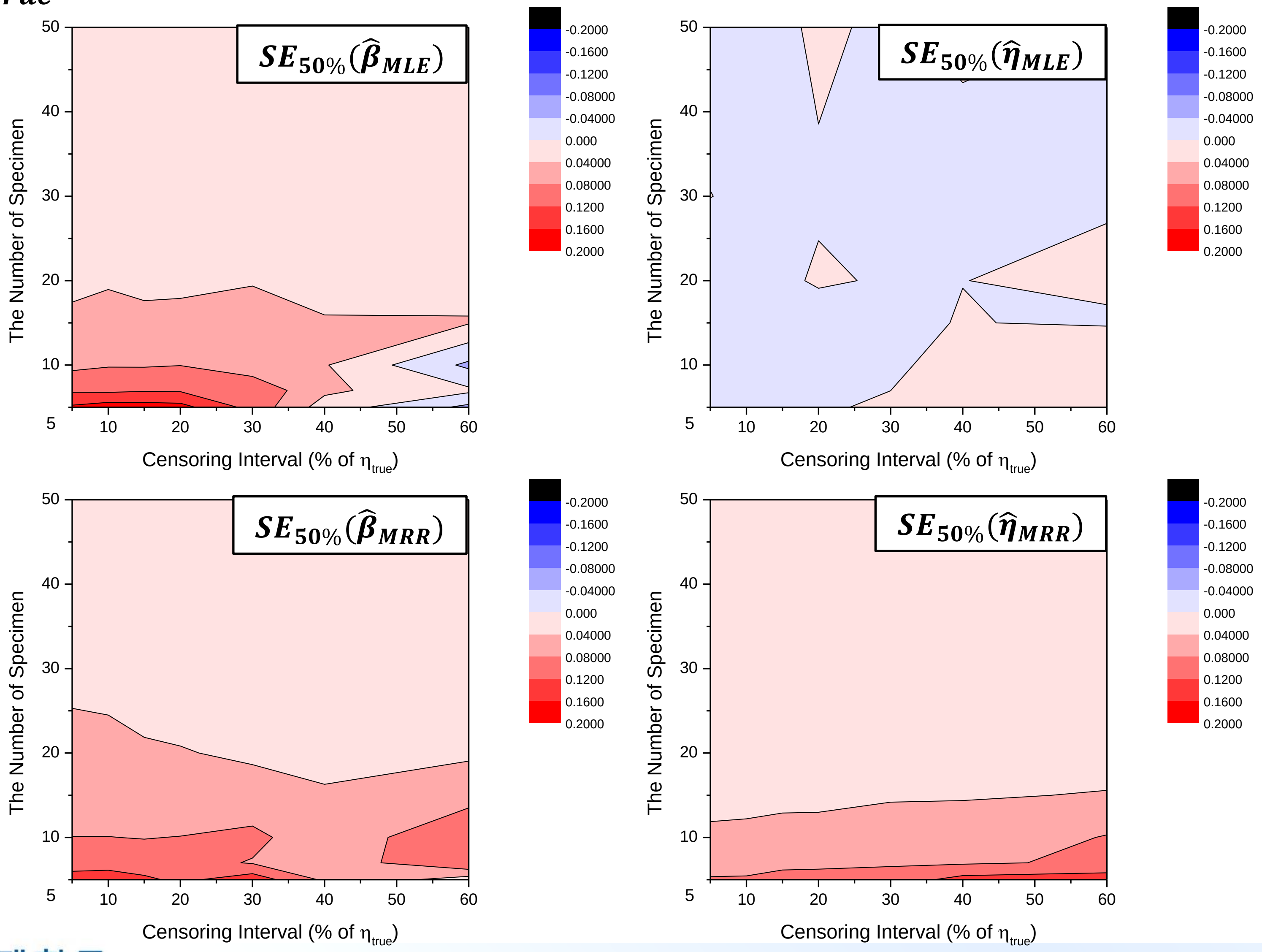

Fixed Test Duration (120%)

- $\beta_{true} = 4$

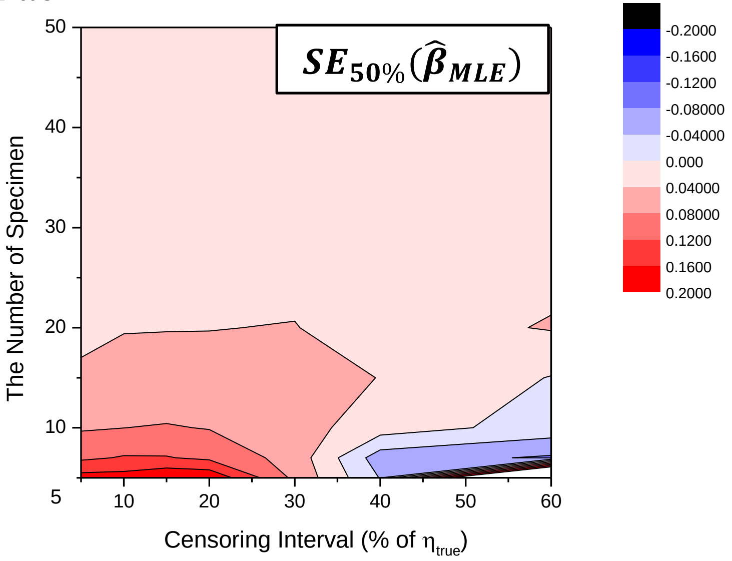

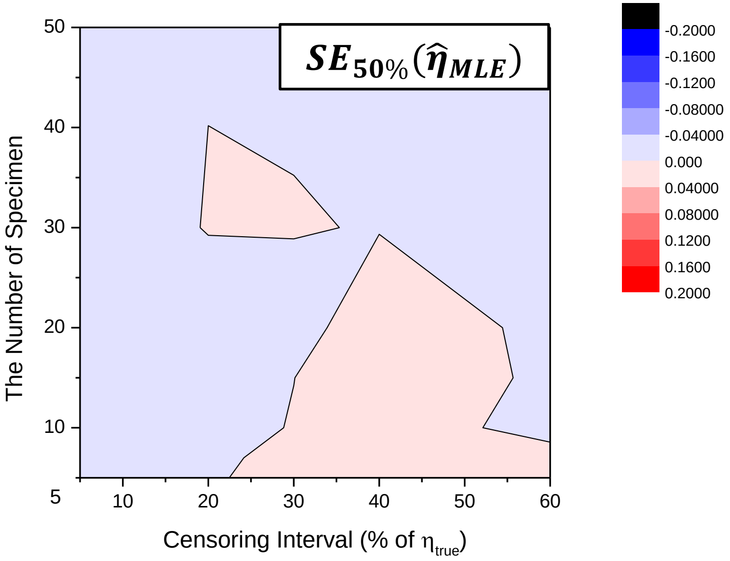

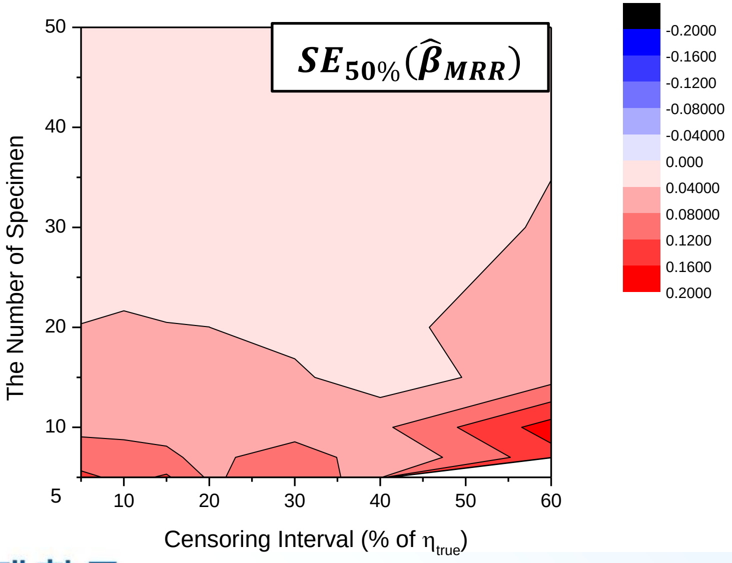

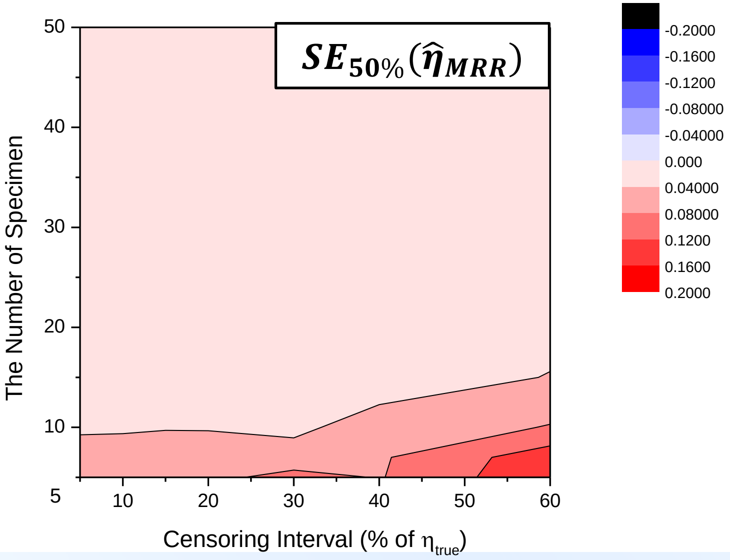

Fixed Test Duration (120%)

- $\beta_{true} = 2$

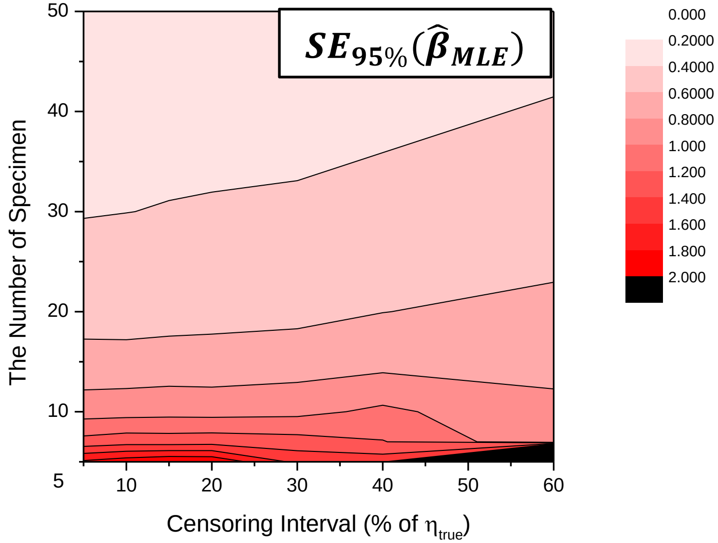

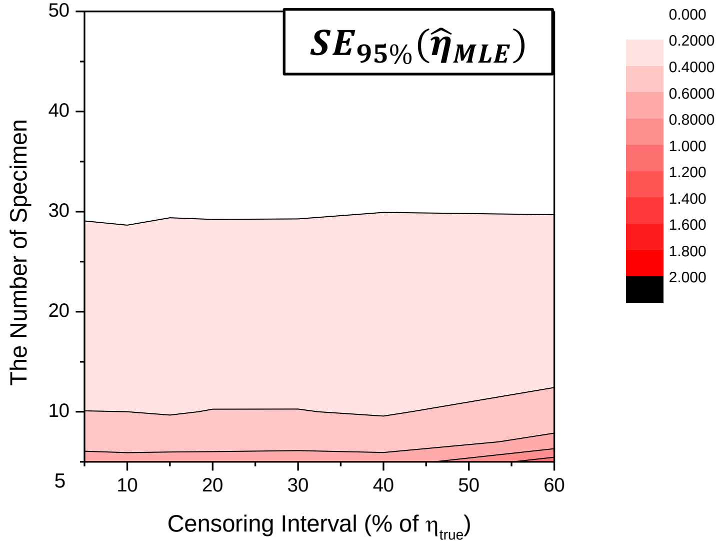

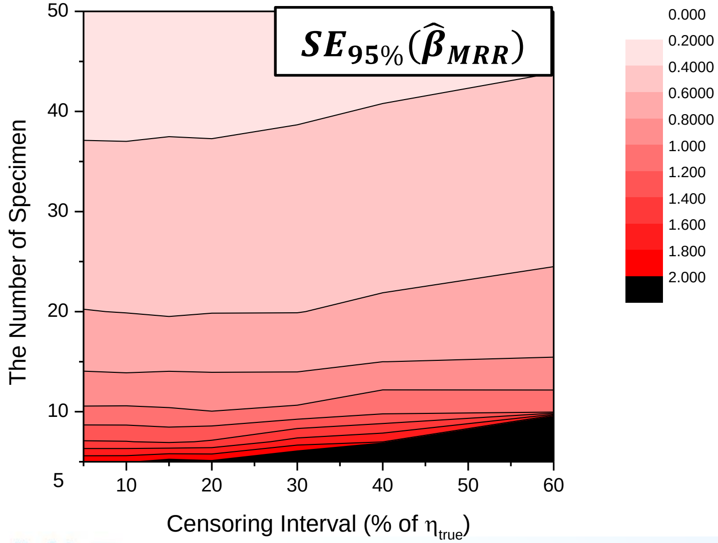

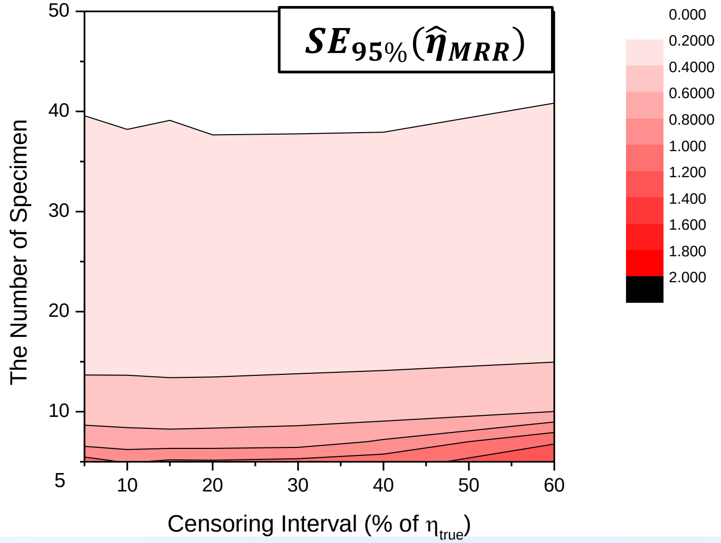

Fixed Test Duration (120%)

- $\beta_{true} = 3$

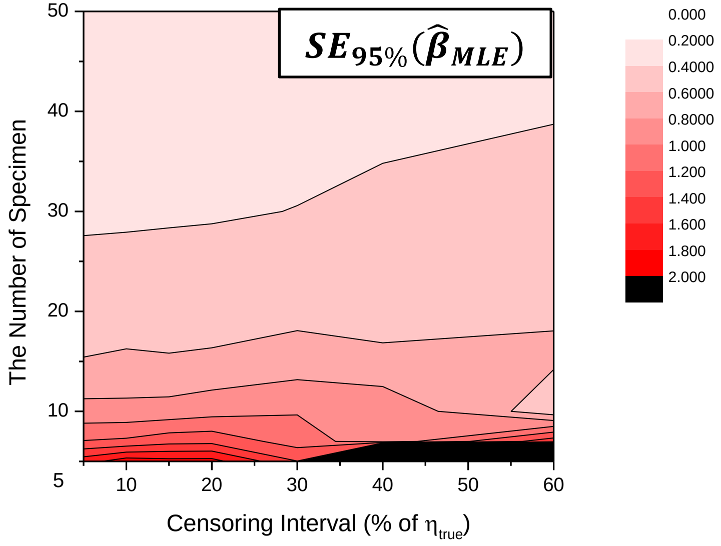

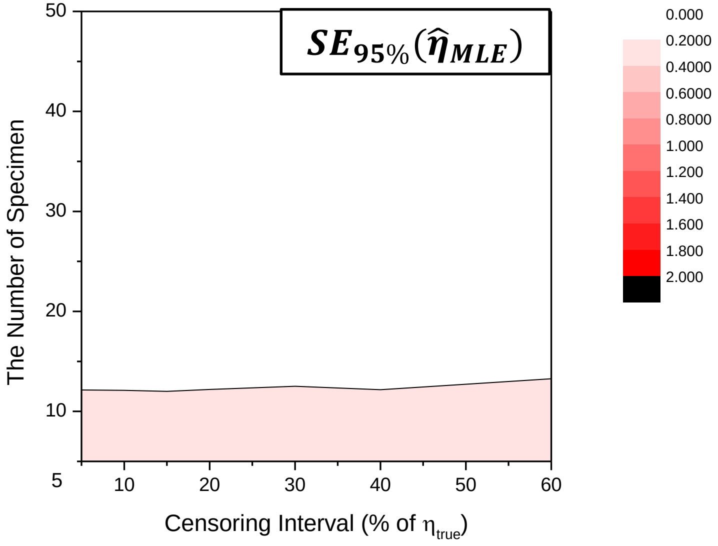

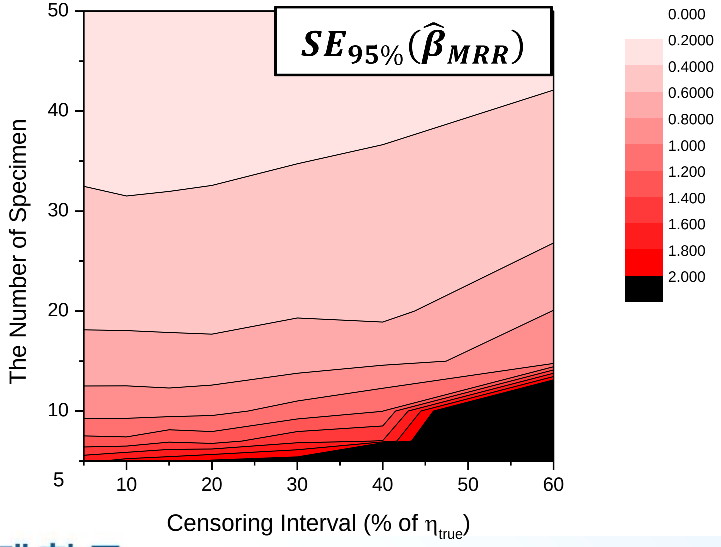

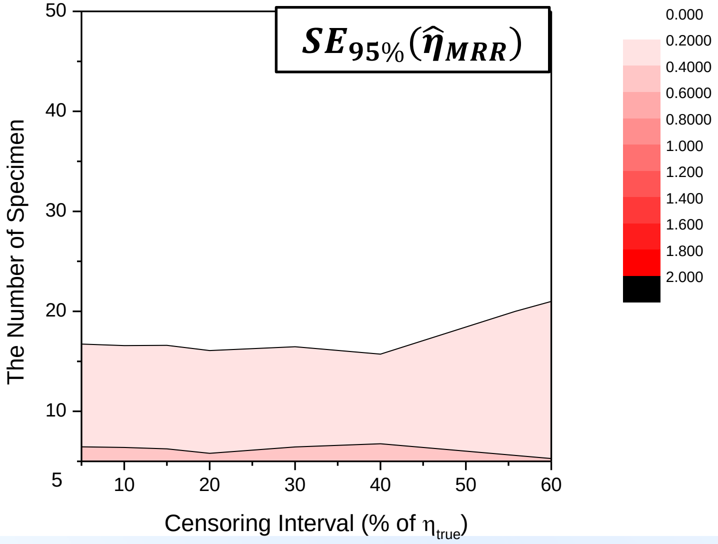

Fixed Test Duration (120%)

- $\beta_{true} = 4$

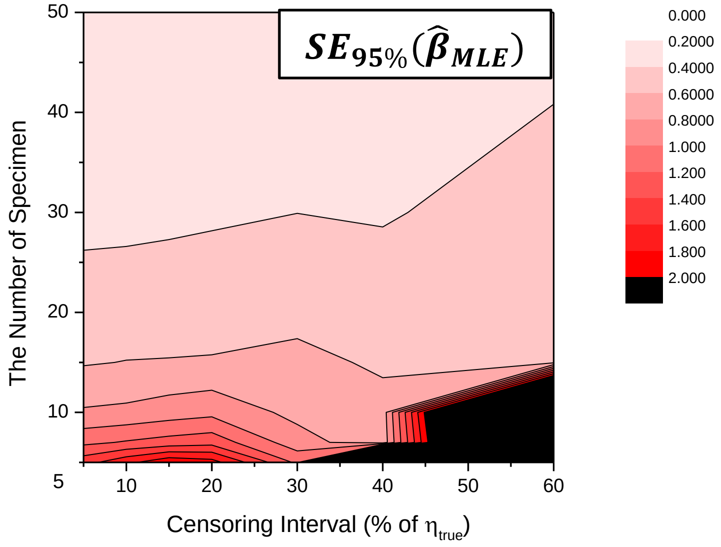

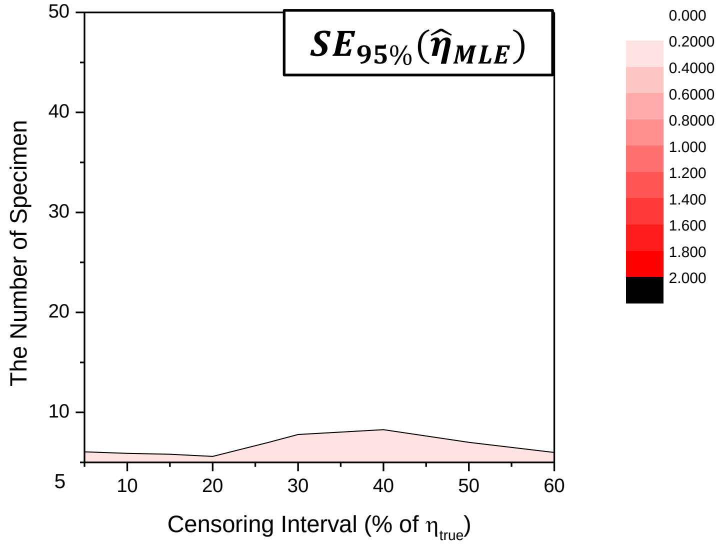

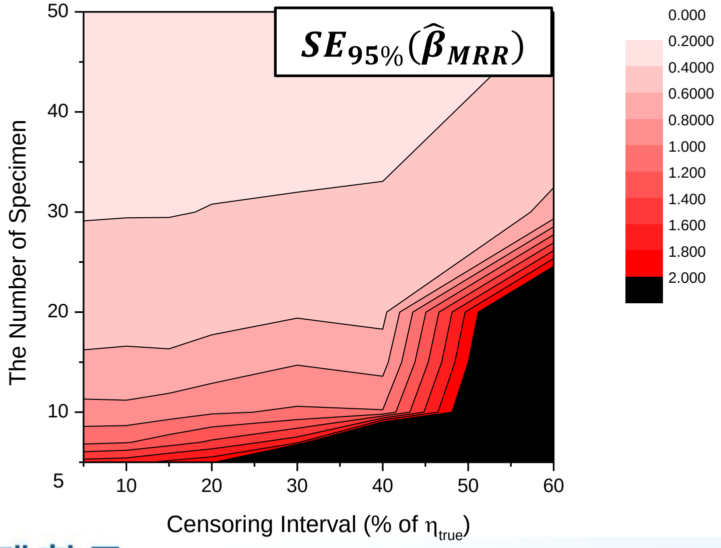

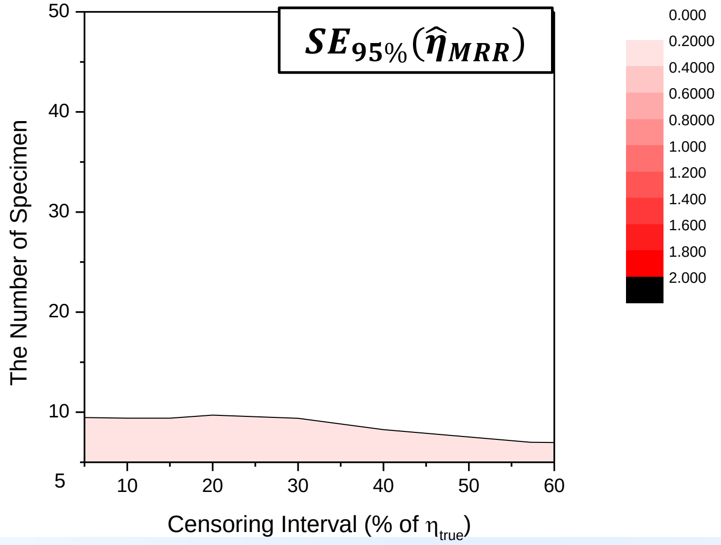

Fixed Test Duration (120%)

- $\beta_{true} = 2$

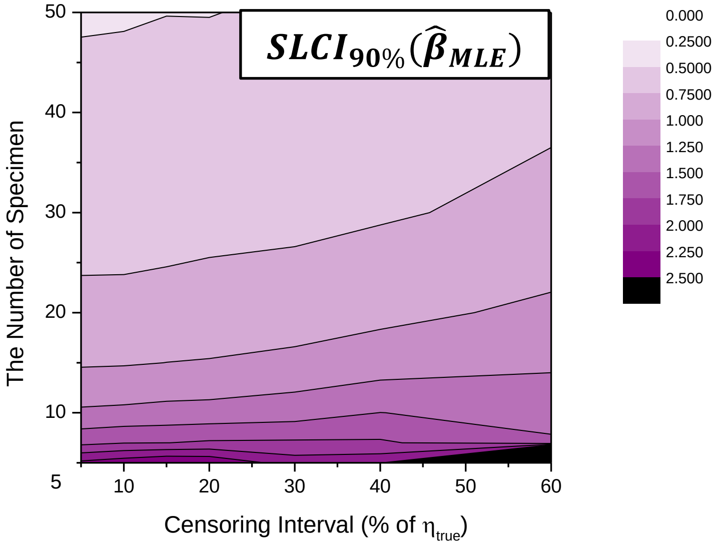

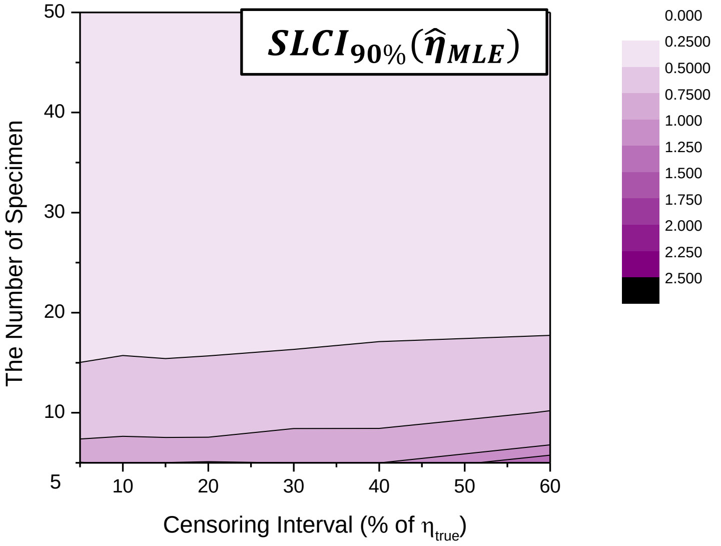

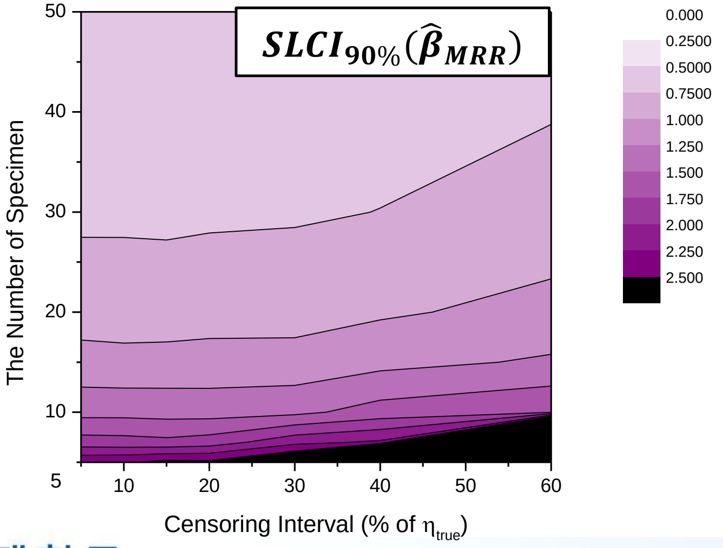

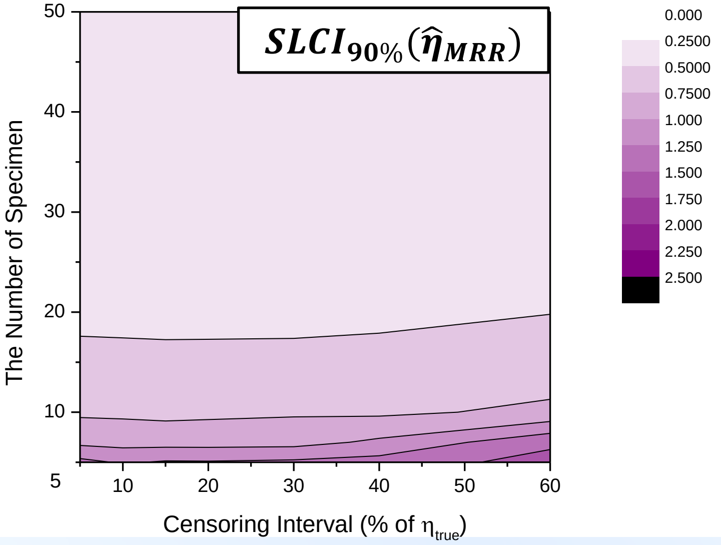

Fixed Test Duration (120%)

- $\beta_{true} = 3$

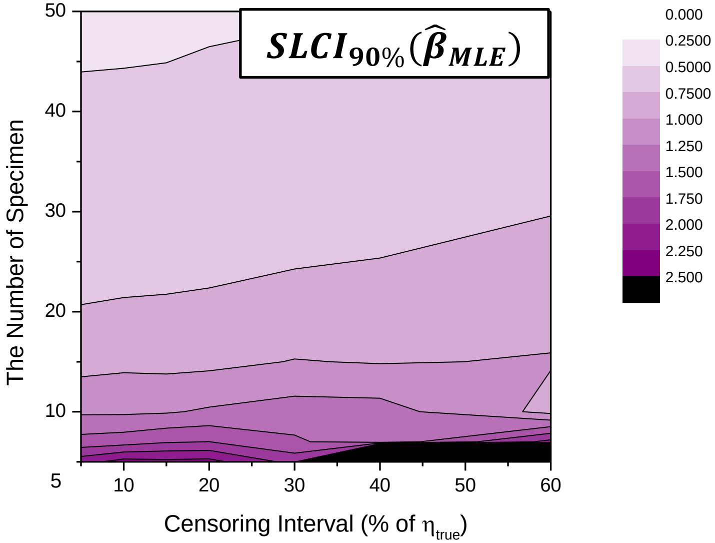

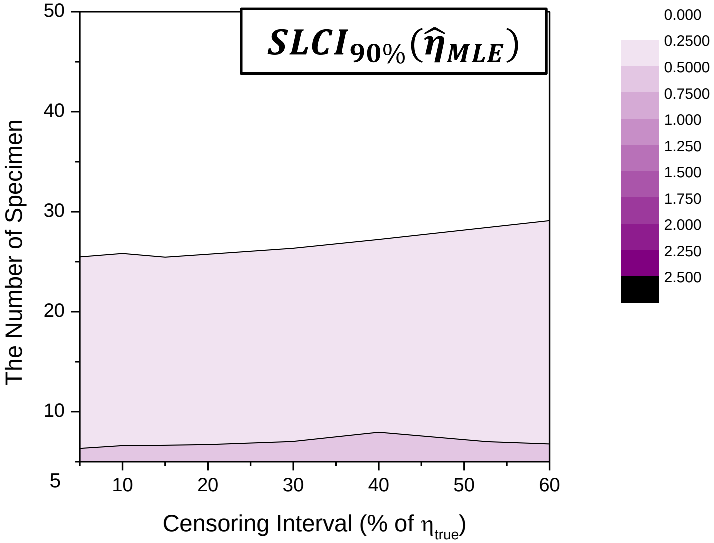

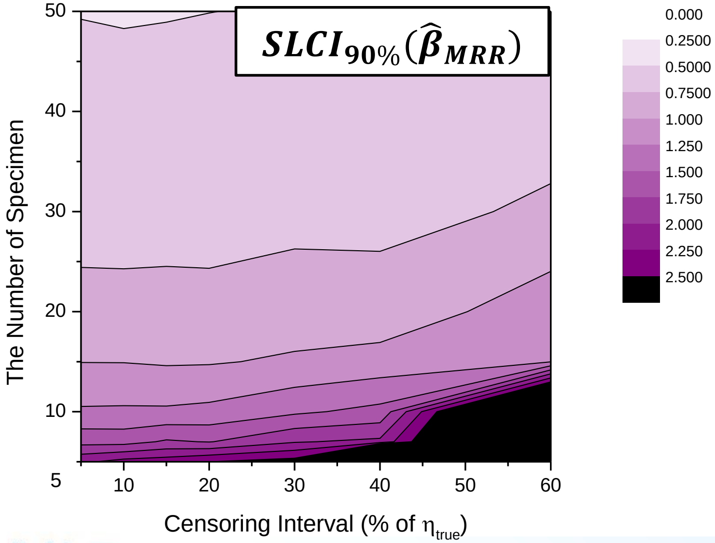

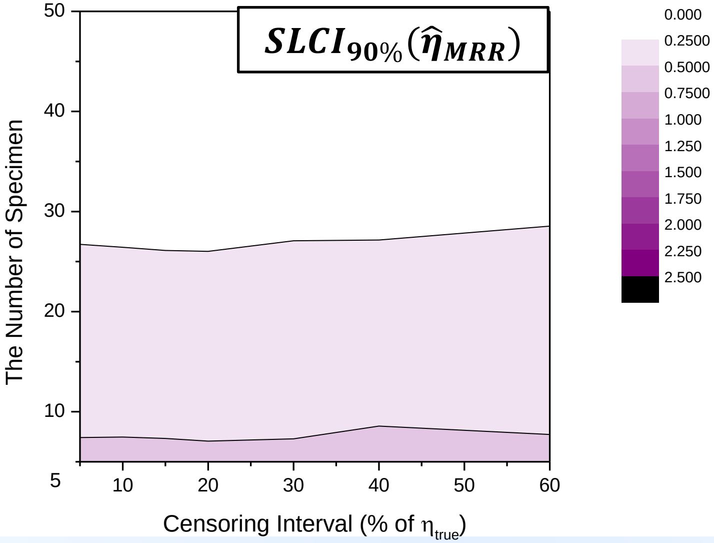

Fixed Test Duration (120%)

- $\beta_{true} = 4$

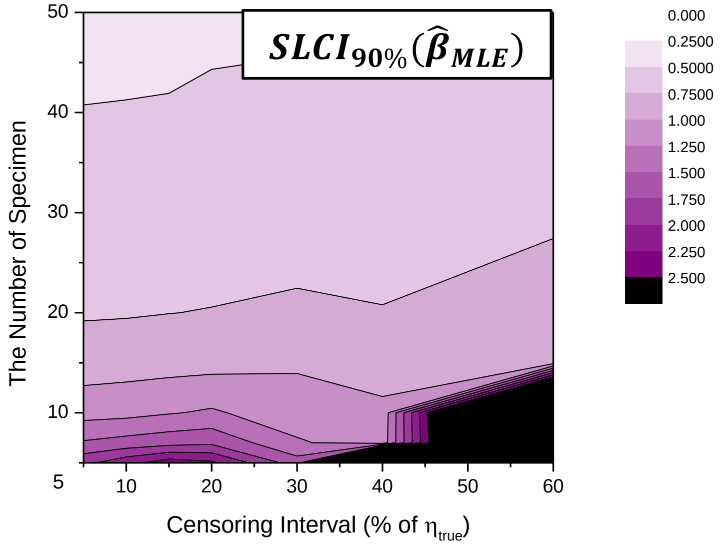

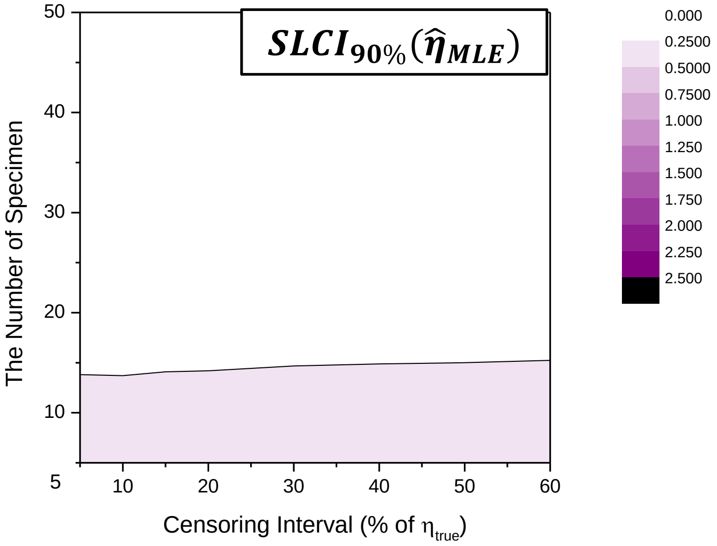

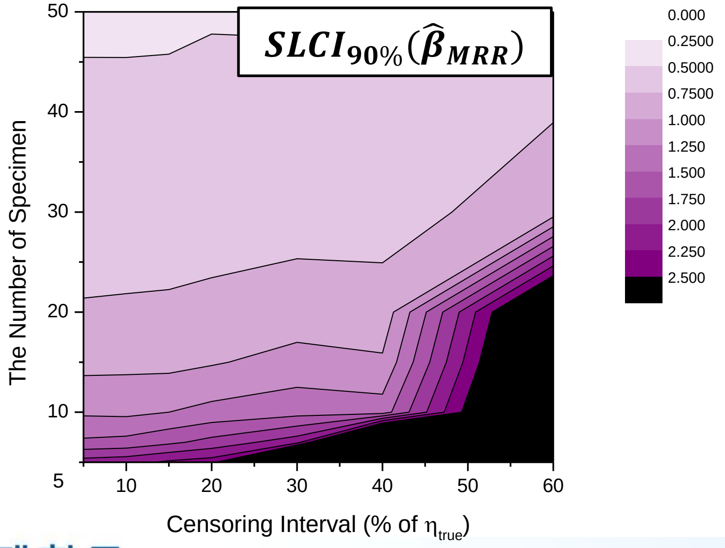

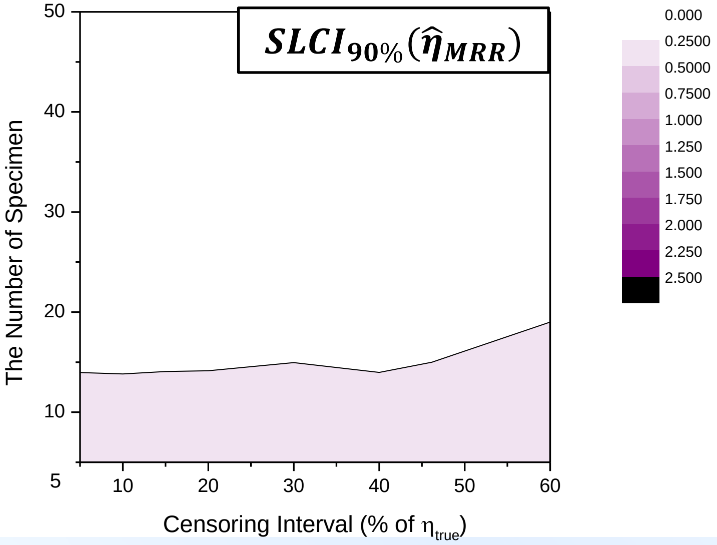

Fixed Censoring Interval (20%)

- $\beta_{true} = 2$
  - ✓ Convergence ratio

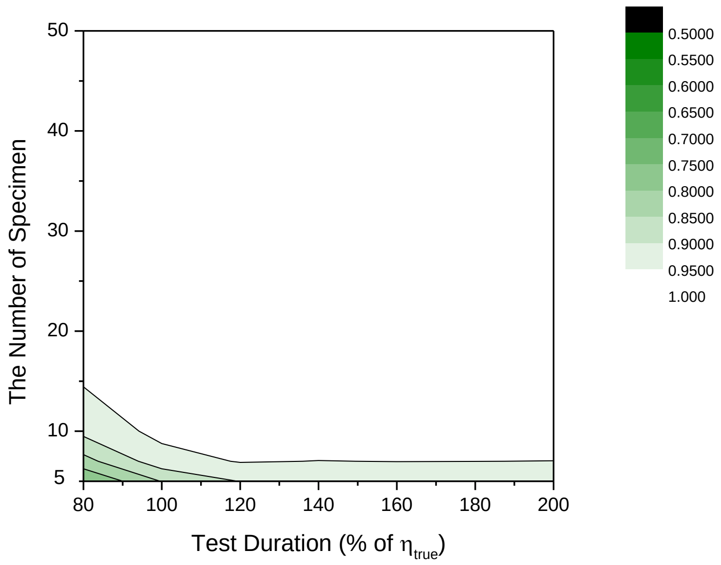

Fixed Censoring Interval (20%)

- $\beta_{true} = 3$
  - ✓ Convergence ratio

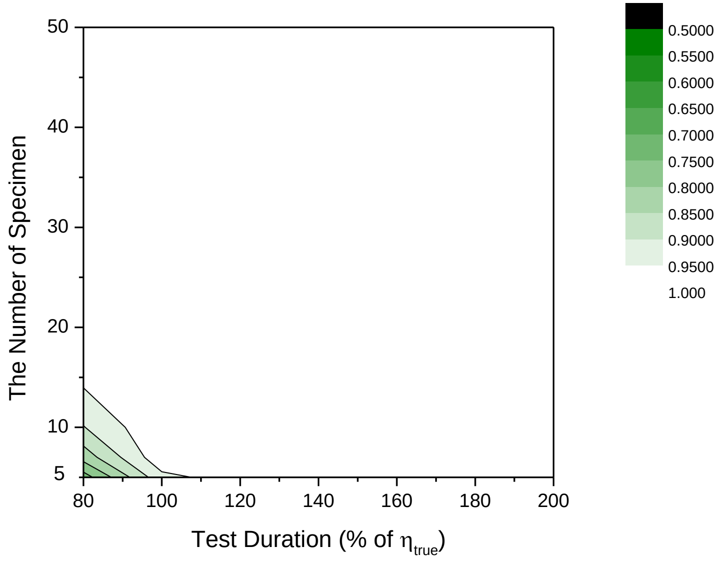

Fixed Censoring Interval (20%)

- $\beta_{true} = 4$
  - ✓ Convergence ratio

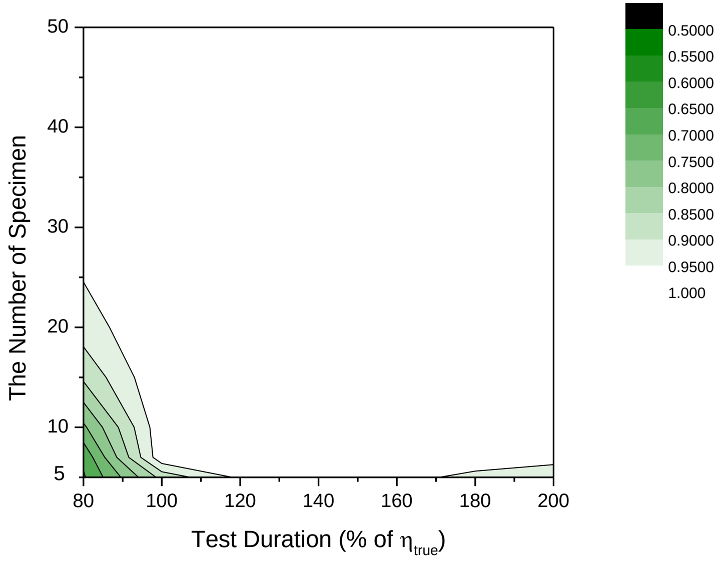

Fixed Censoring Interval (20%)

- $\beta_{true} = 2$

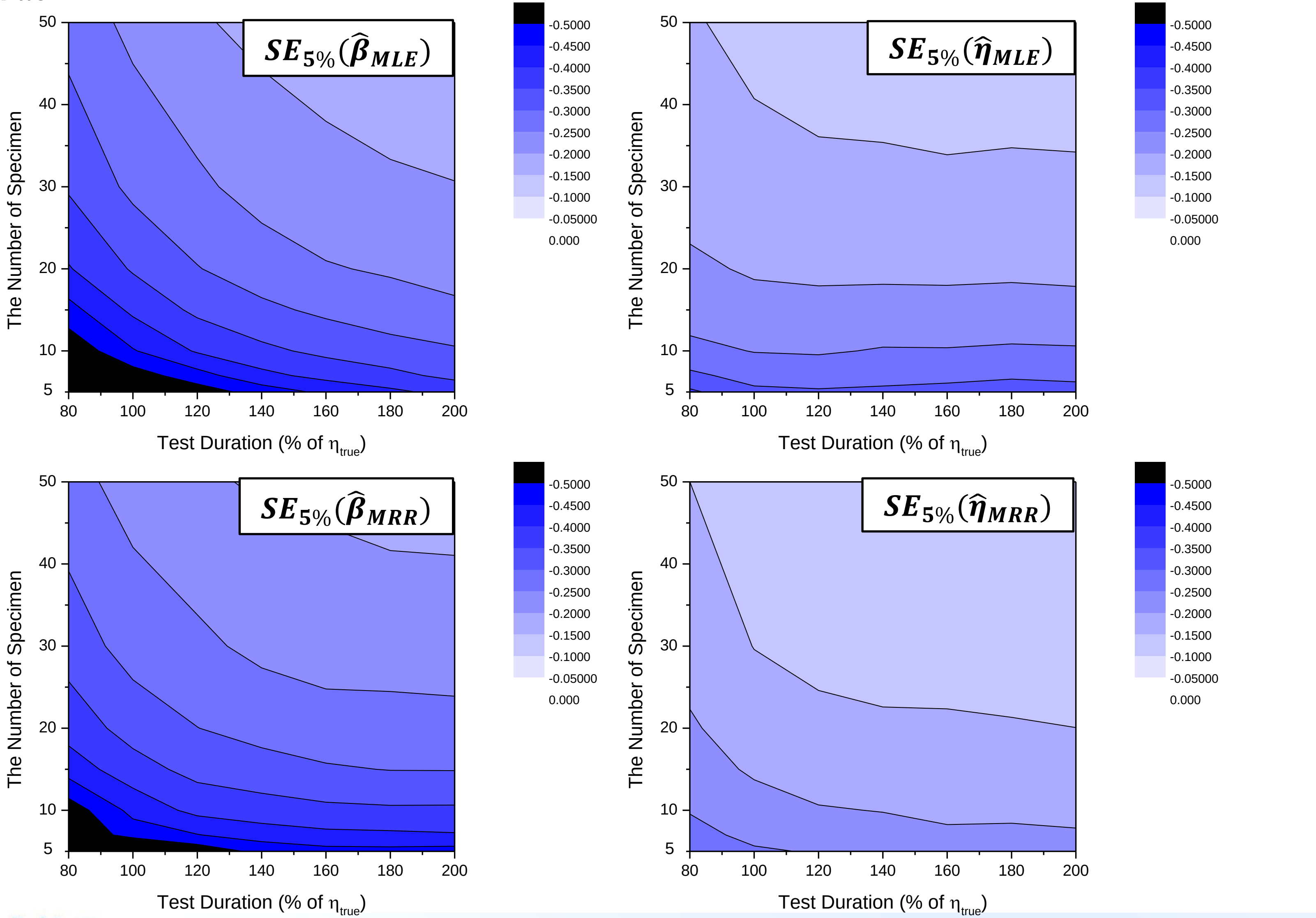

Fixed Censoring Interval (20%)

- $\beta_{true} = 3$

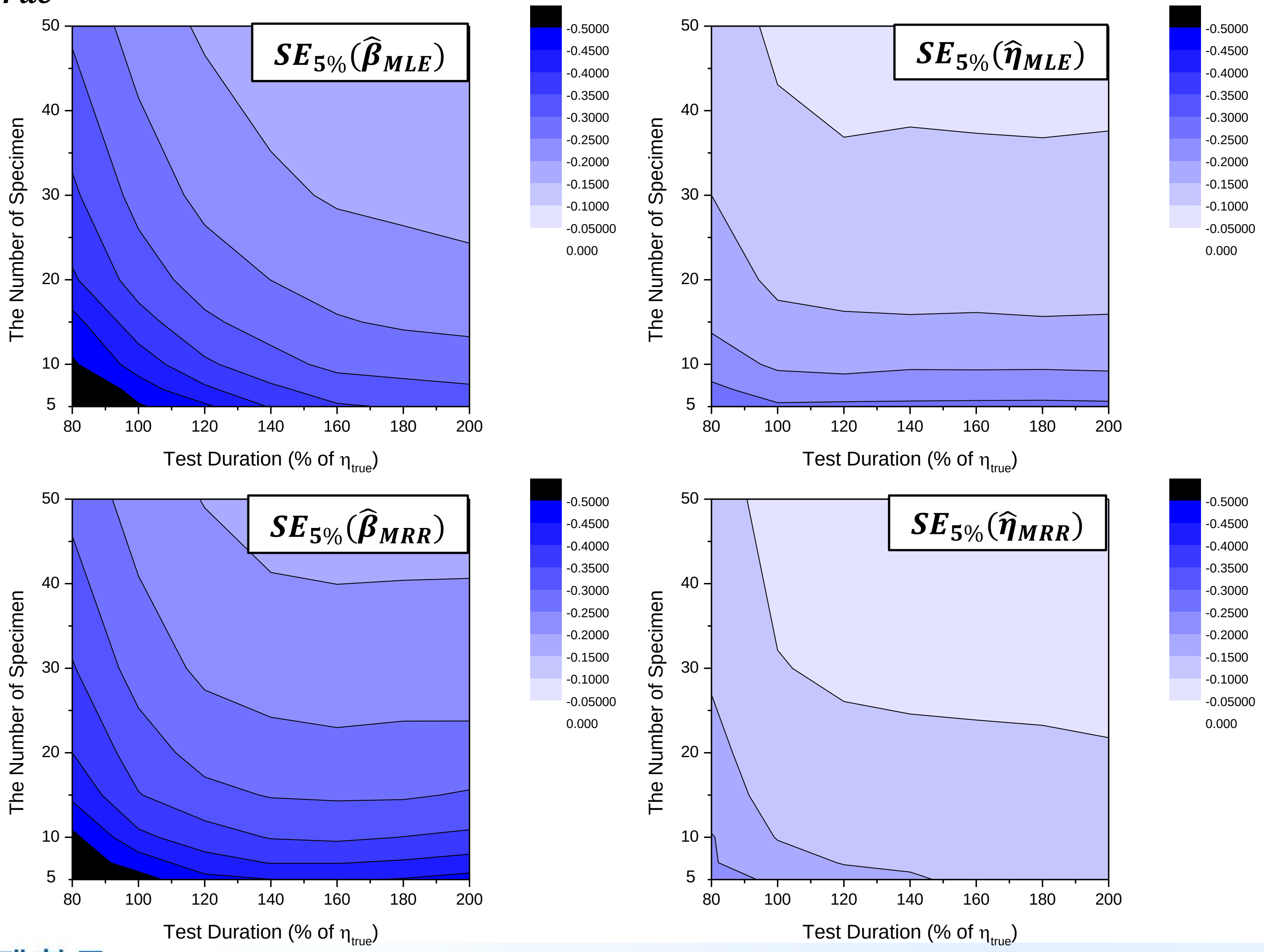

Fixed Censoring Interval (20%)

- $\beta_{true} = 4$

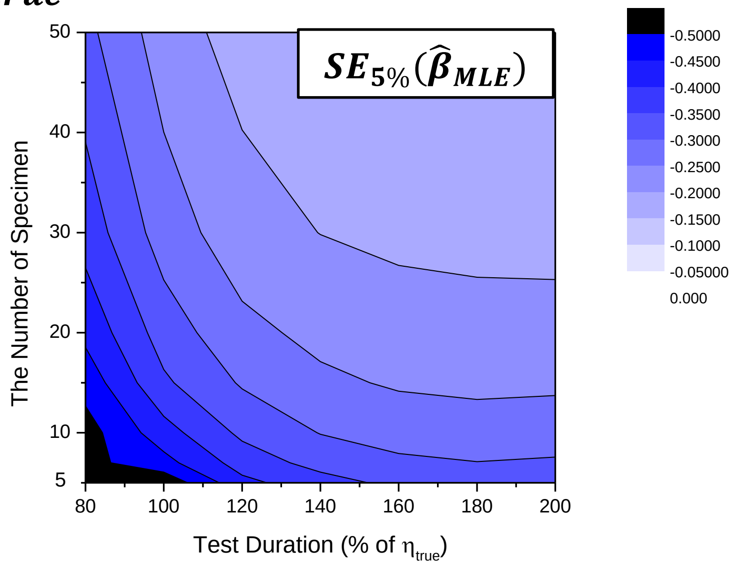

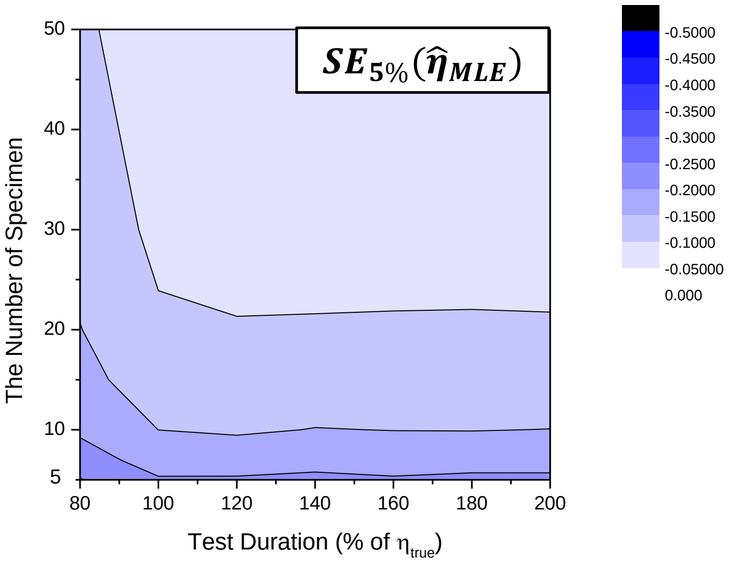

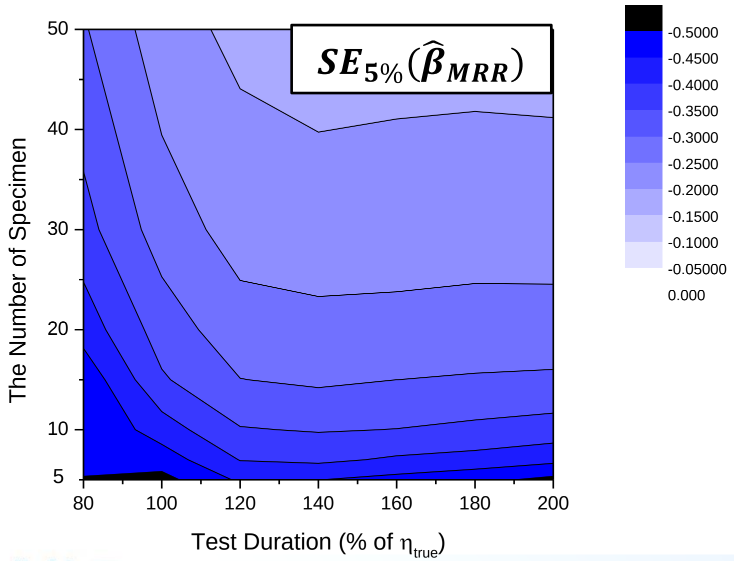

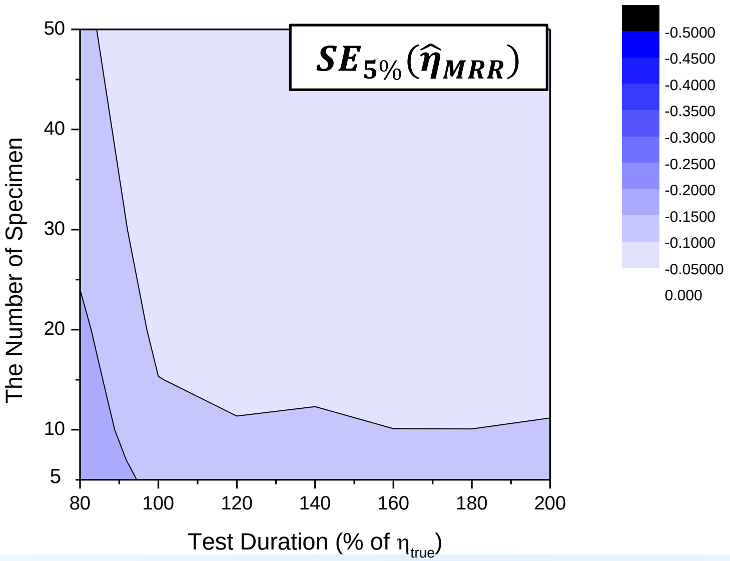

Fixed Censoring Interval (20%)

- $\beta_{true} = 2$

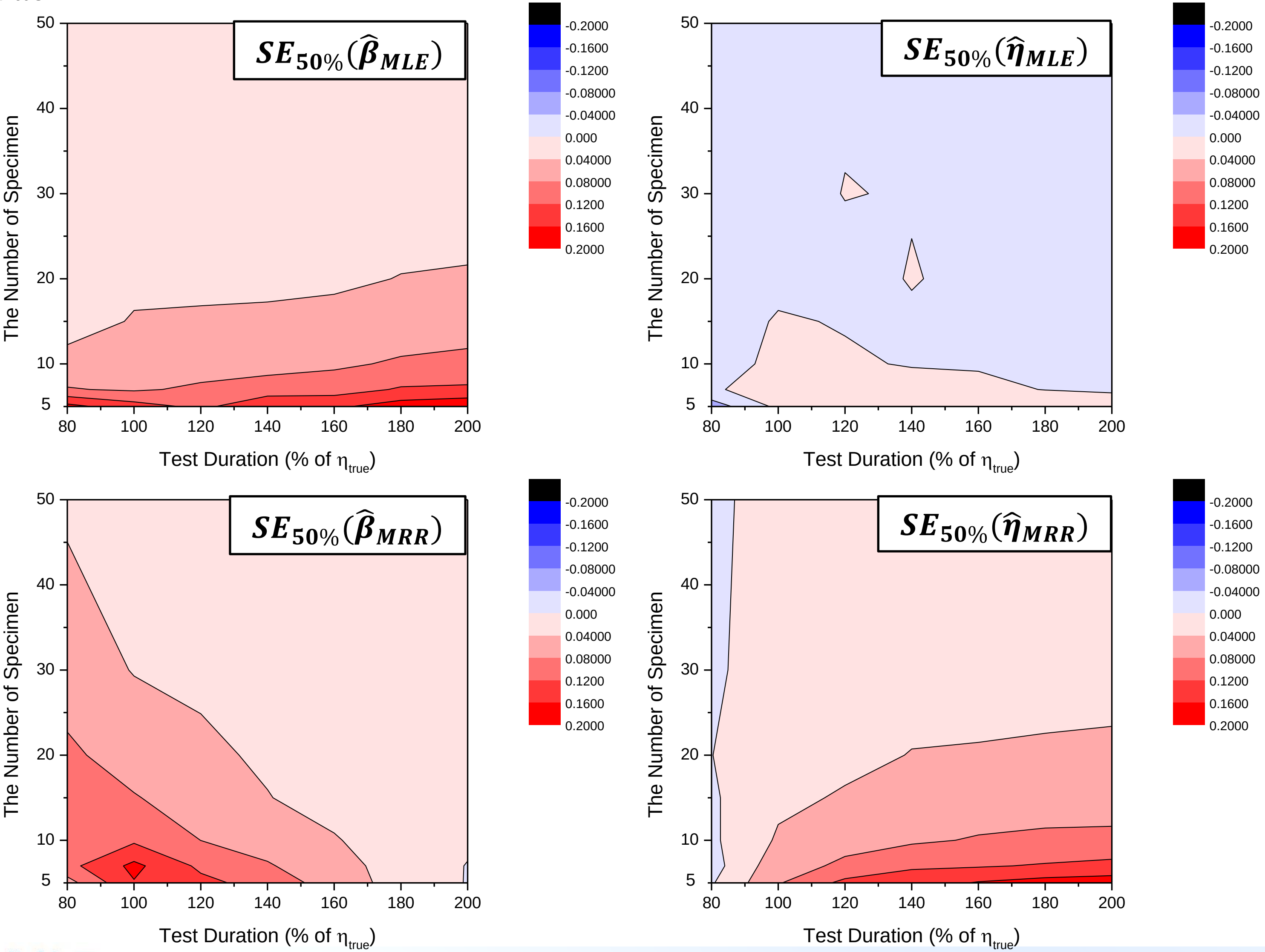

Fixed Censoring Interval (20%)

- $\beta_{true} = 3$

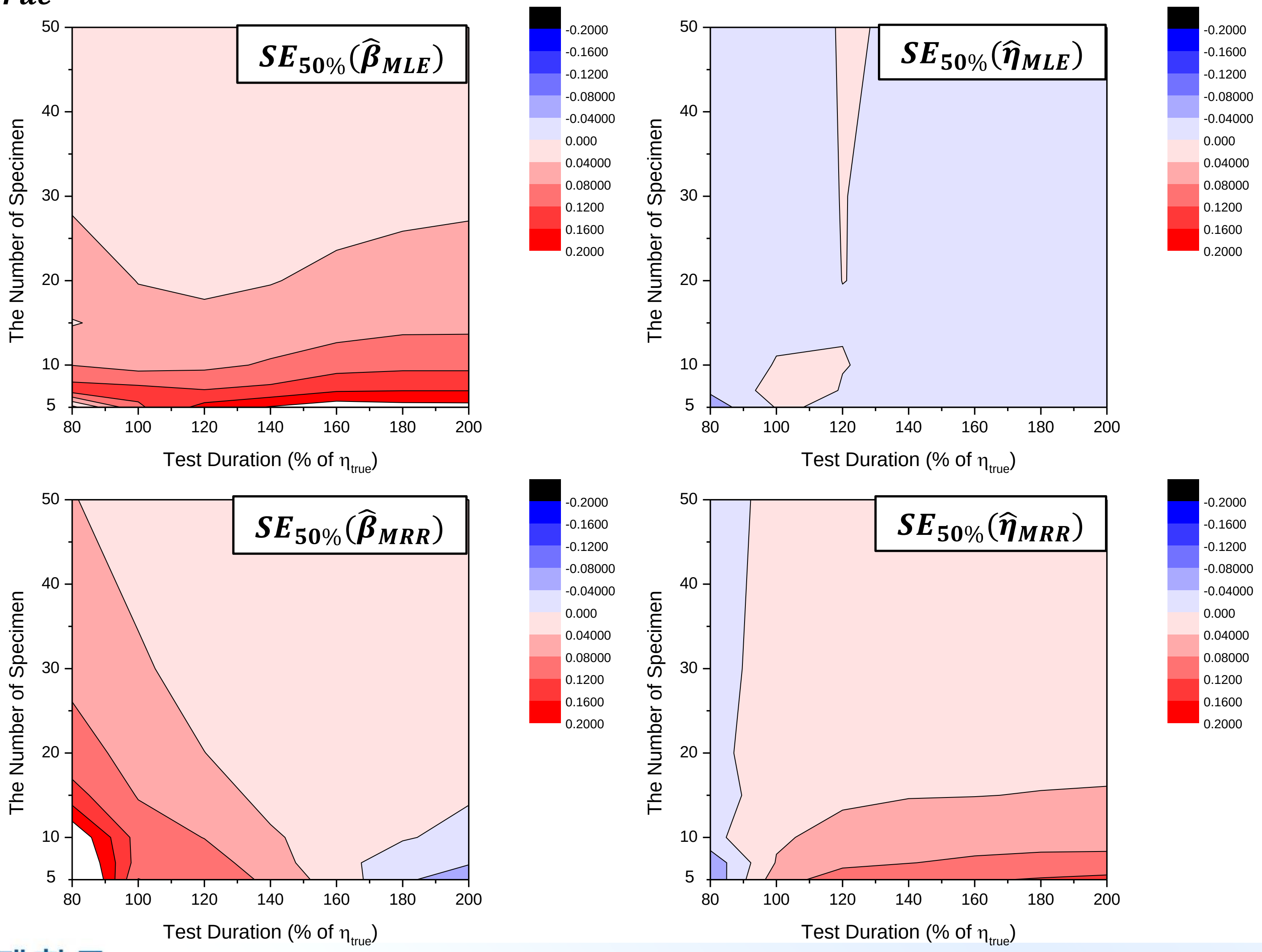

Fixed Censoring Interval (20%)

- $\beta_{true} = 4$

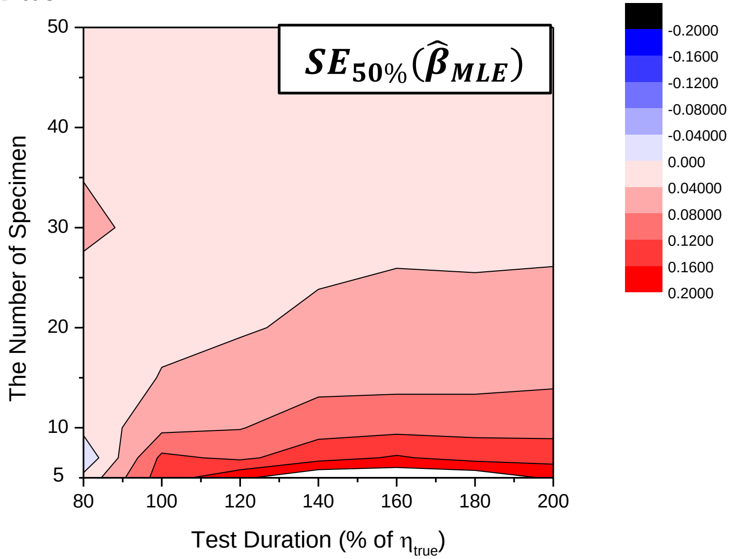

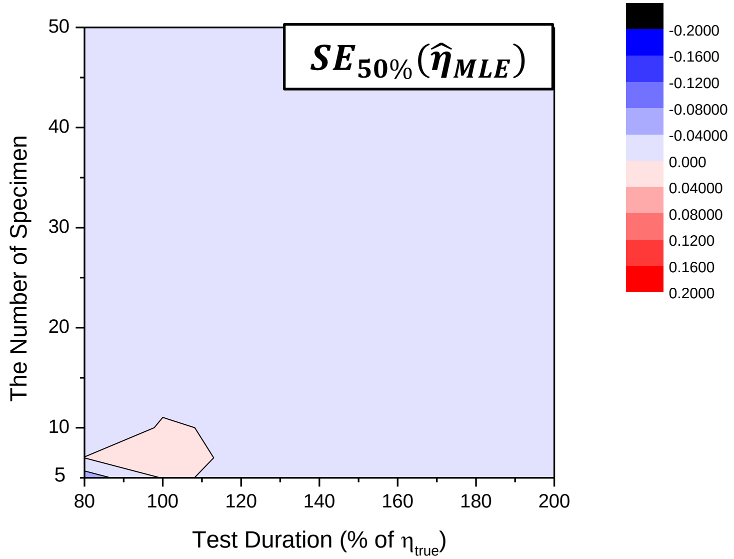

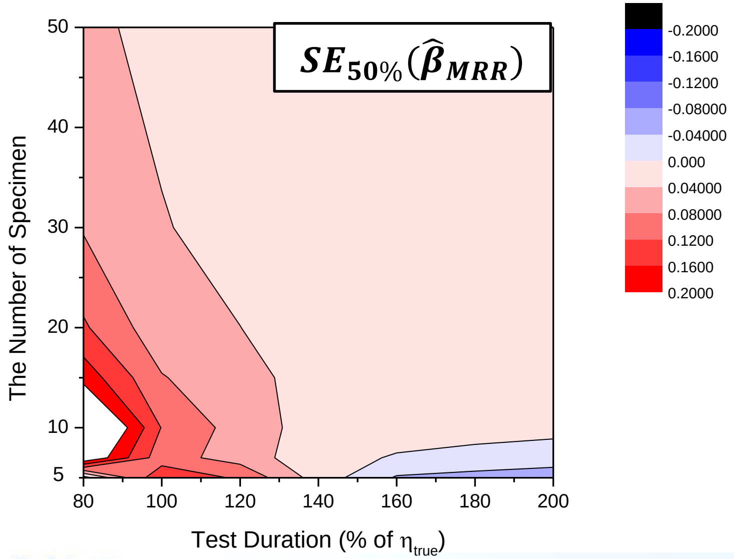

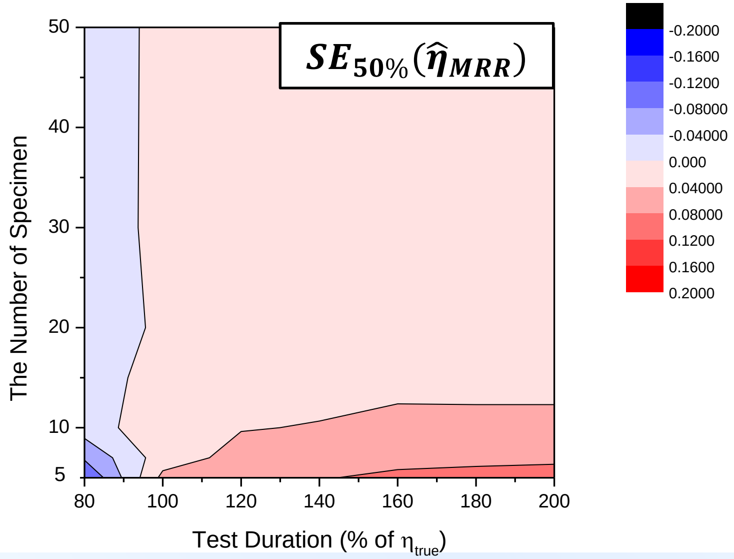

Fixed Censoring Interval (20%)

- $\beta_{true} = 2$

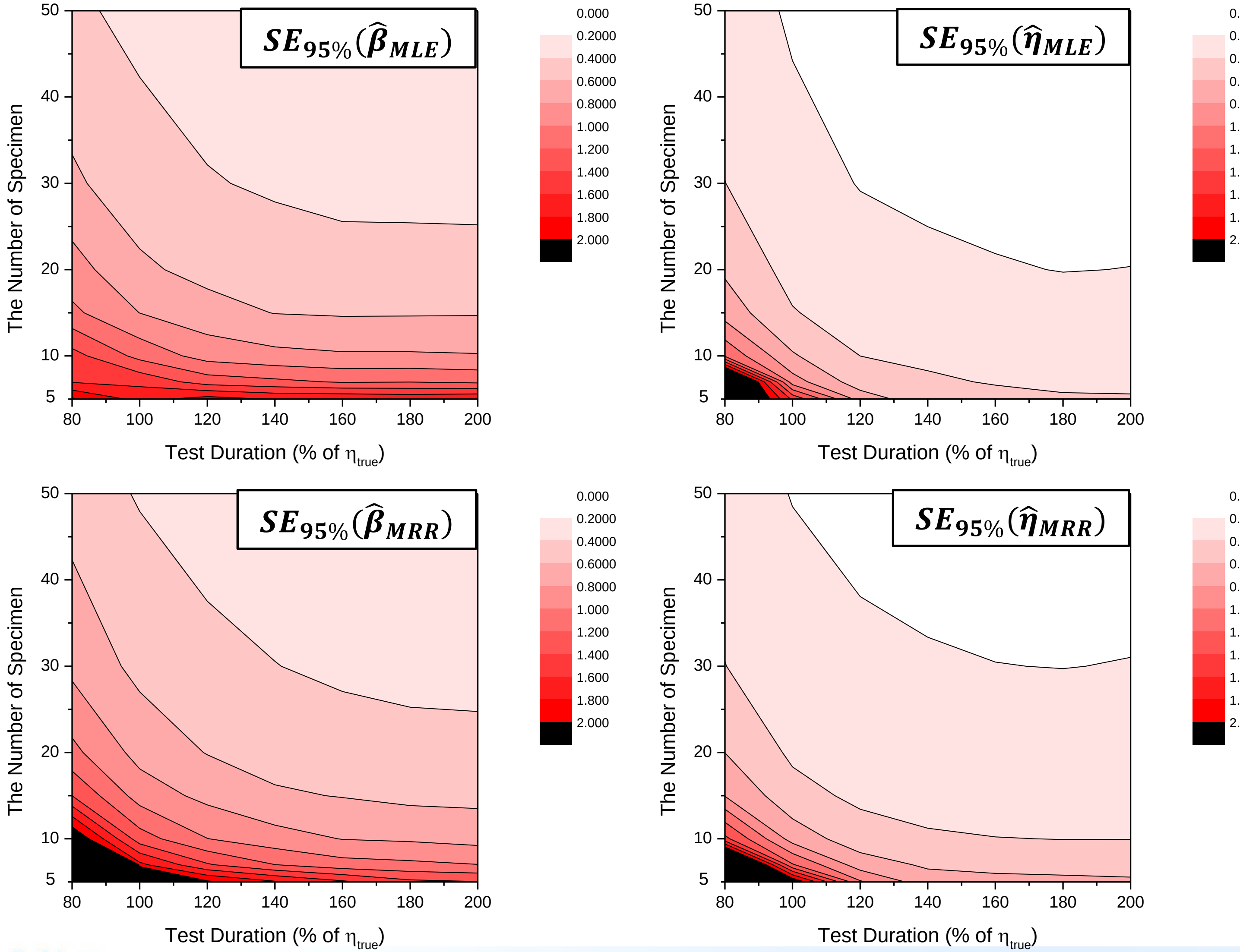

Fixed Censoring Interval (20%)

- $\beta_{true} = 3$

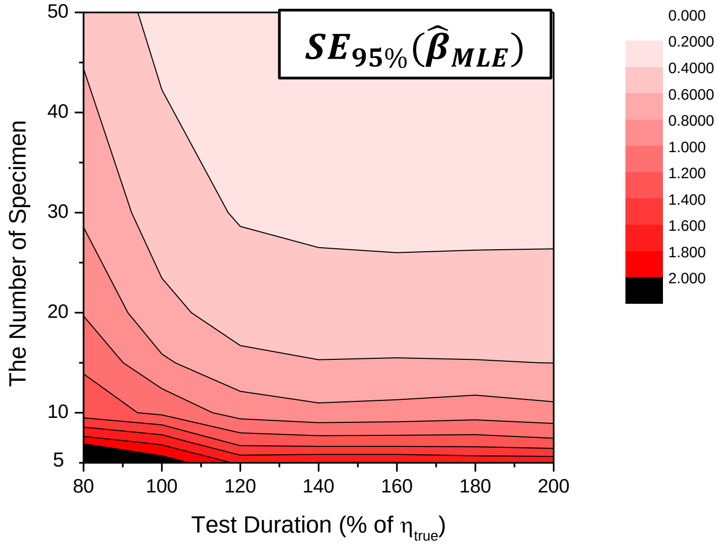

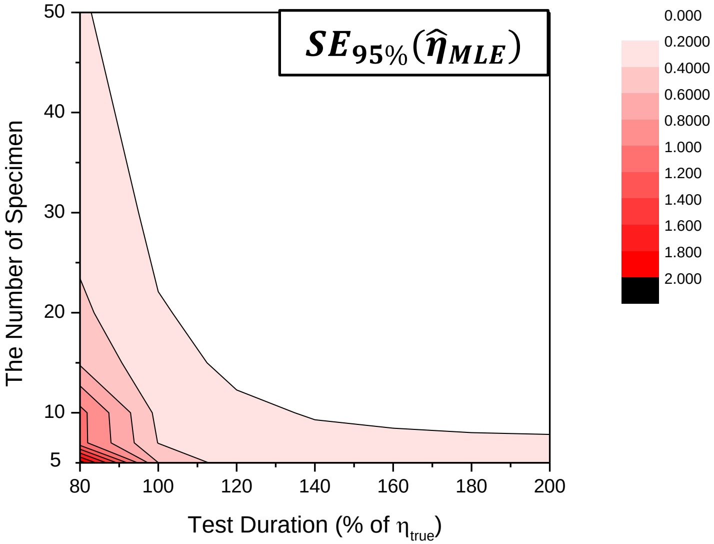

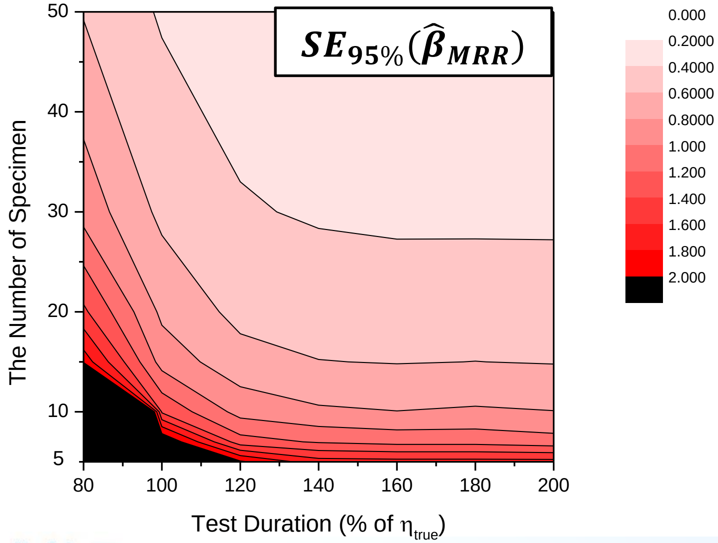

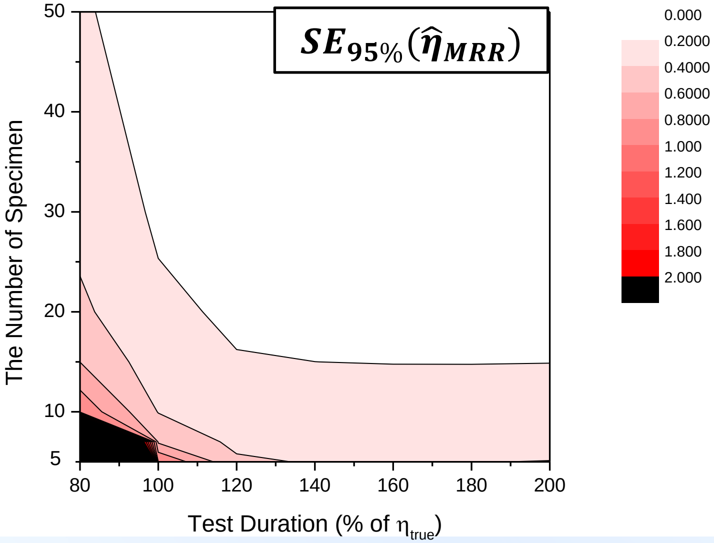

Fixed Censoring Interval (20%)

- $\beta_{true} = 4$

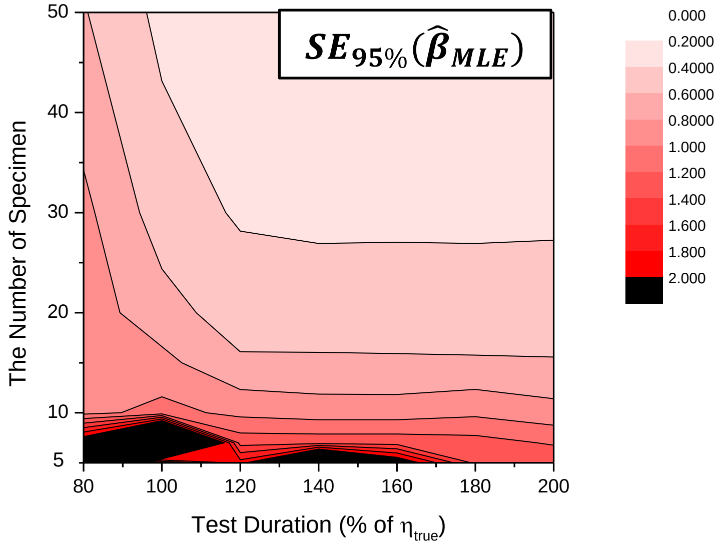

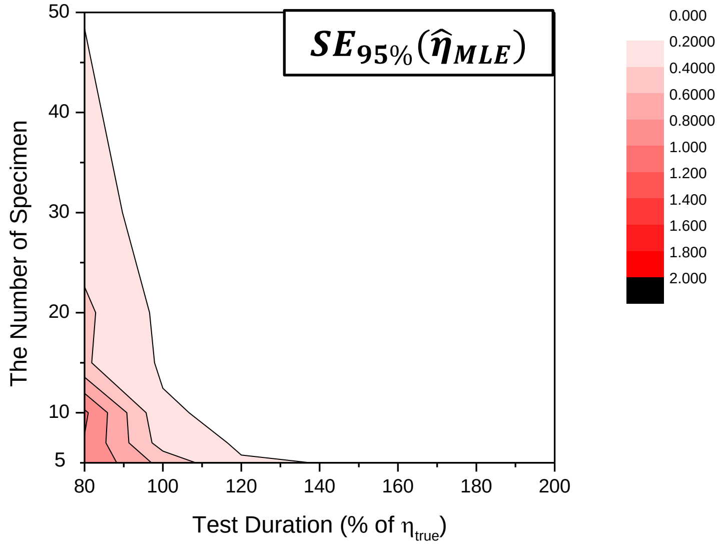

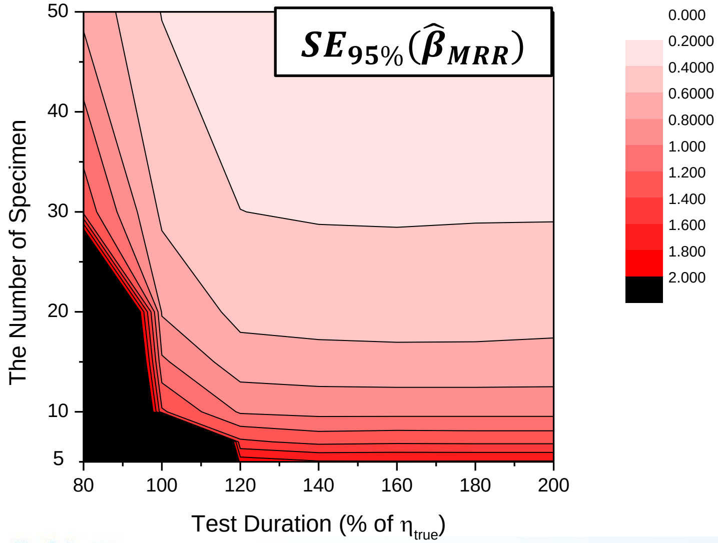

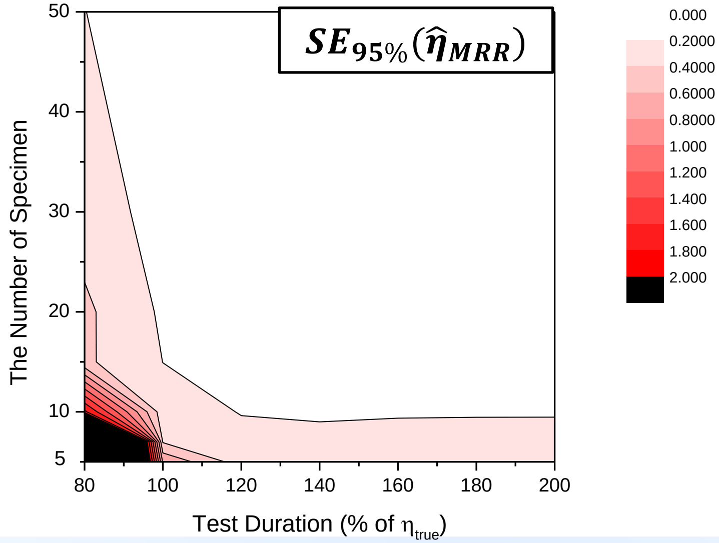

Fixed Censoring Interval (20%)

- $\beta_{true} = 2$

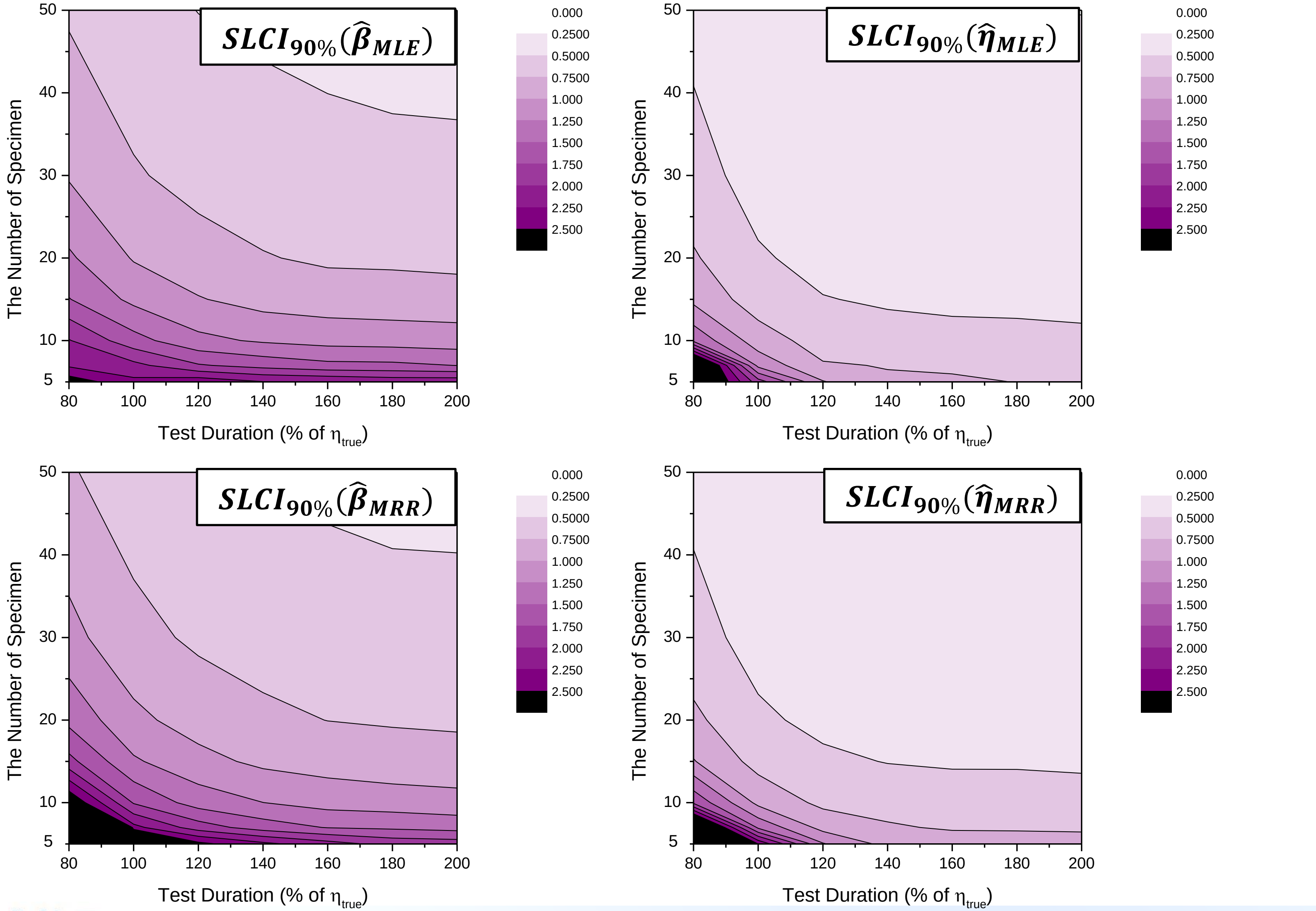

Fixed Censoring Interval (20%)

- $\beta_{true} = 3$

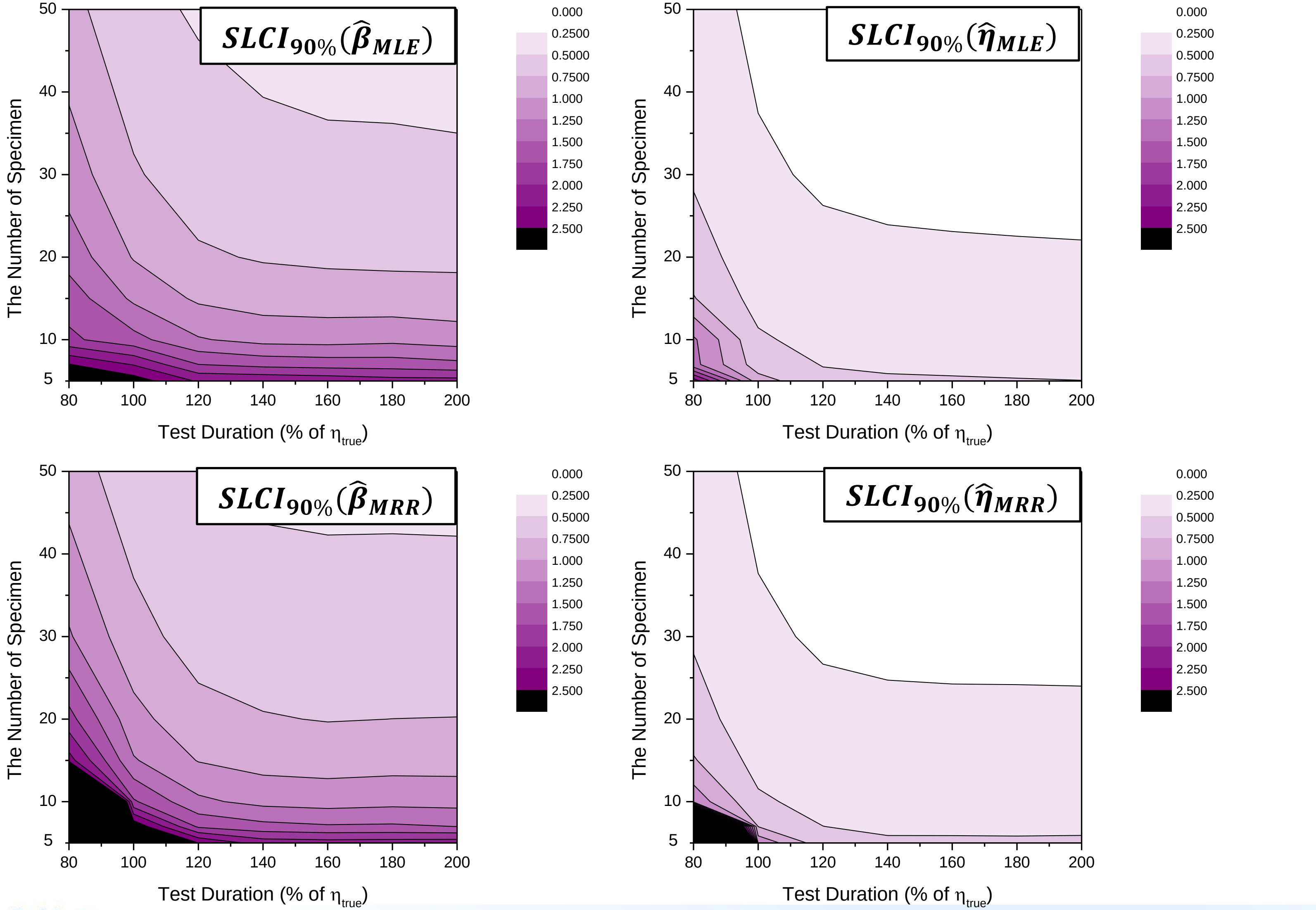

Fixed Censoring Interval (20%)

- $\beta_{true} = 4$

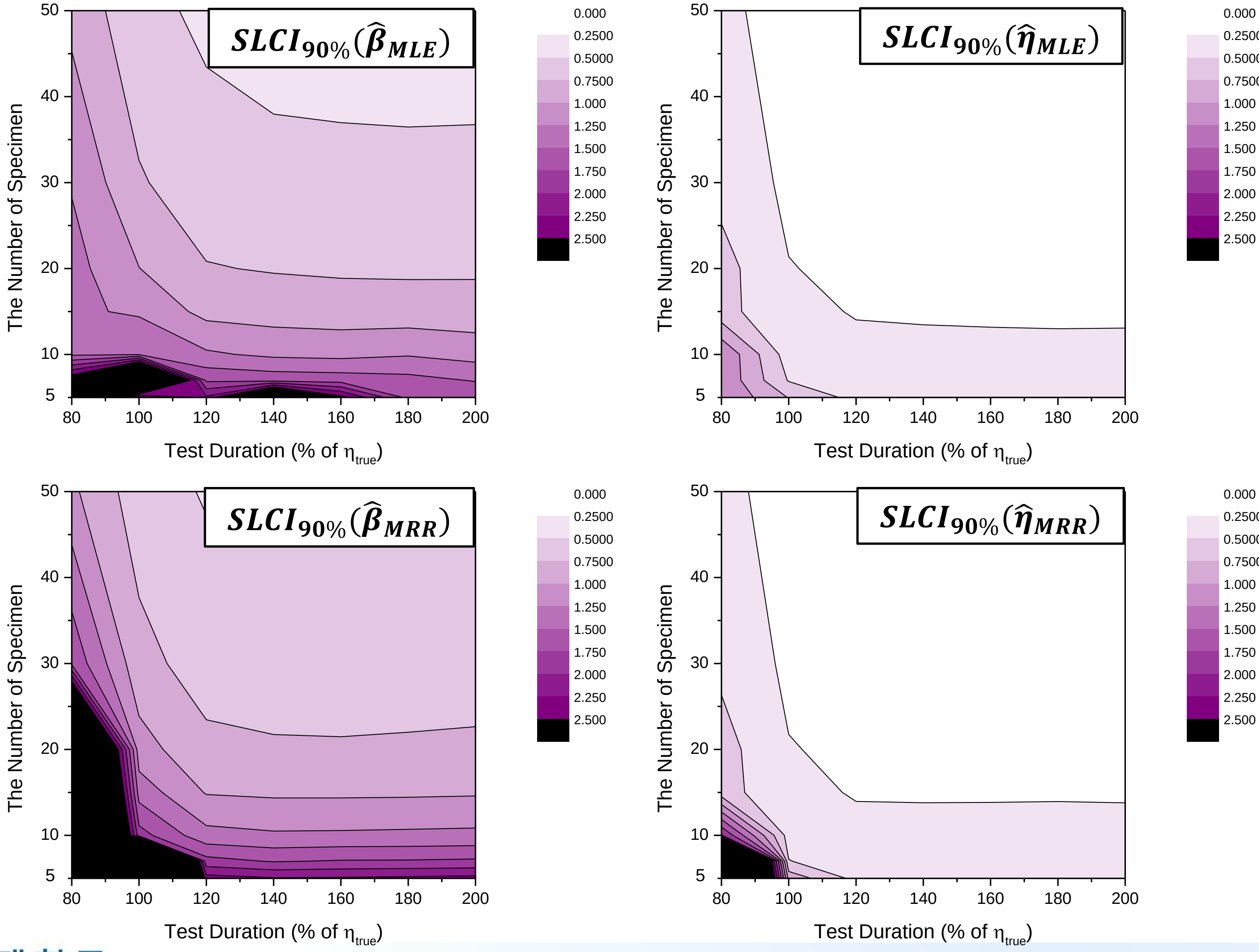

Fixed Specimen Number (10 ea.)

- **Censoring Times**

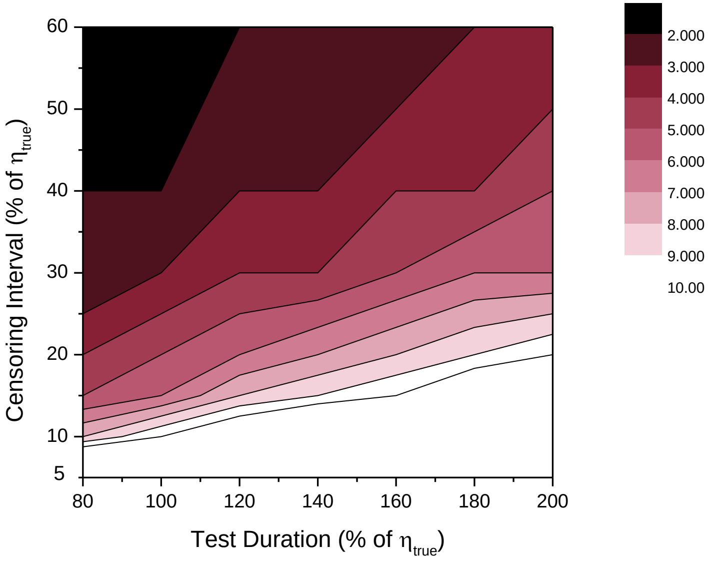

Fixed Specimen Number (10 ea.)

- $\beta_{true} = 2$
  - ✓ Convergence ratio

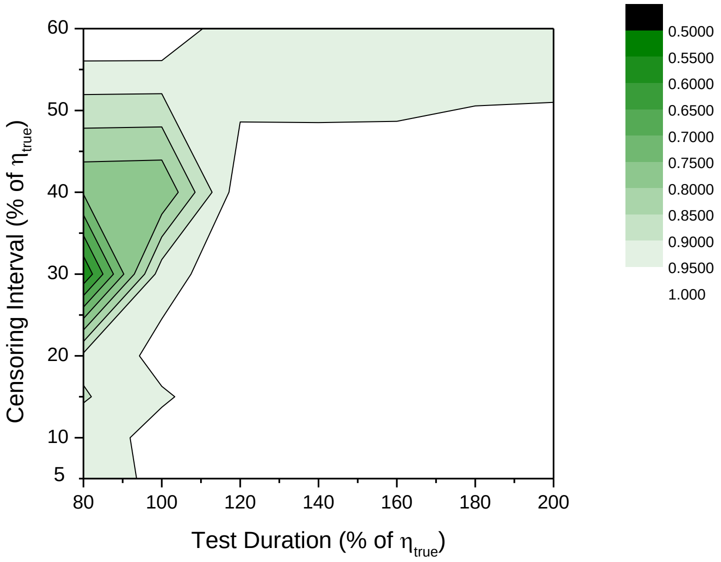

Fixed Specimen Number (10 ea.)

- $\beta_{true} = 3$
  - ✓ Convergence ratio

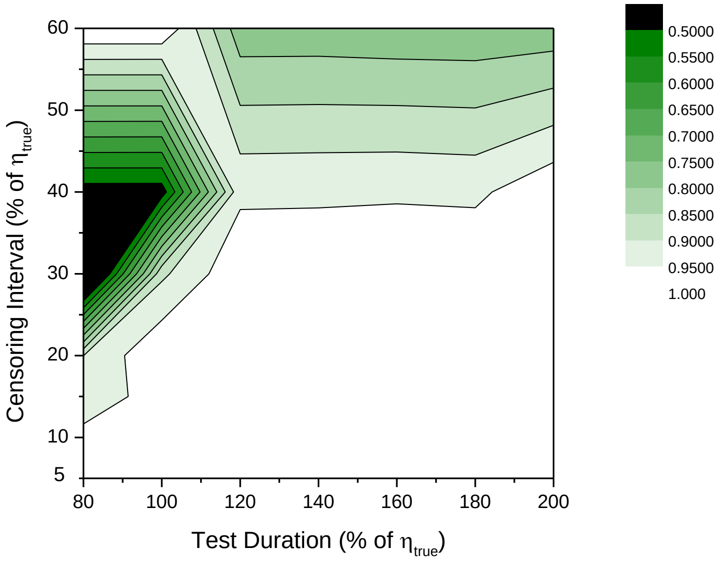

Fixed Specimen Number (10 ea.)

- $\beta_{true} = 4$
  - ✓ Convergence ratio

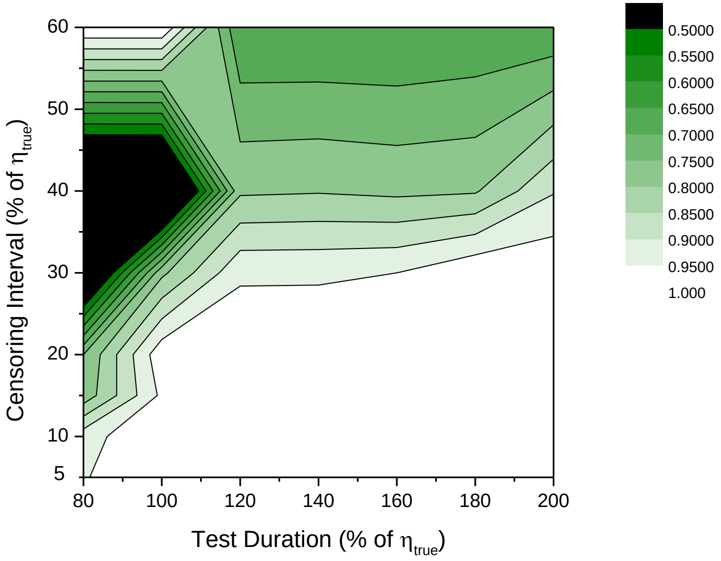

Fixed Specimen Number (10 ea.)

- $\beta_{true} = 2$

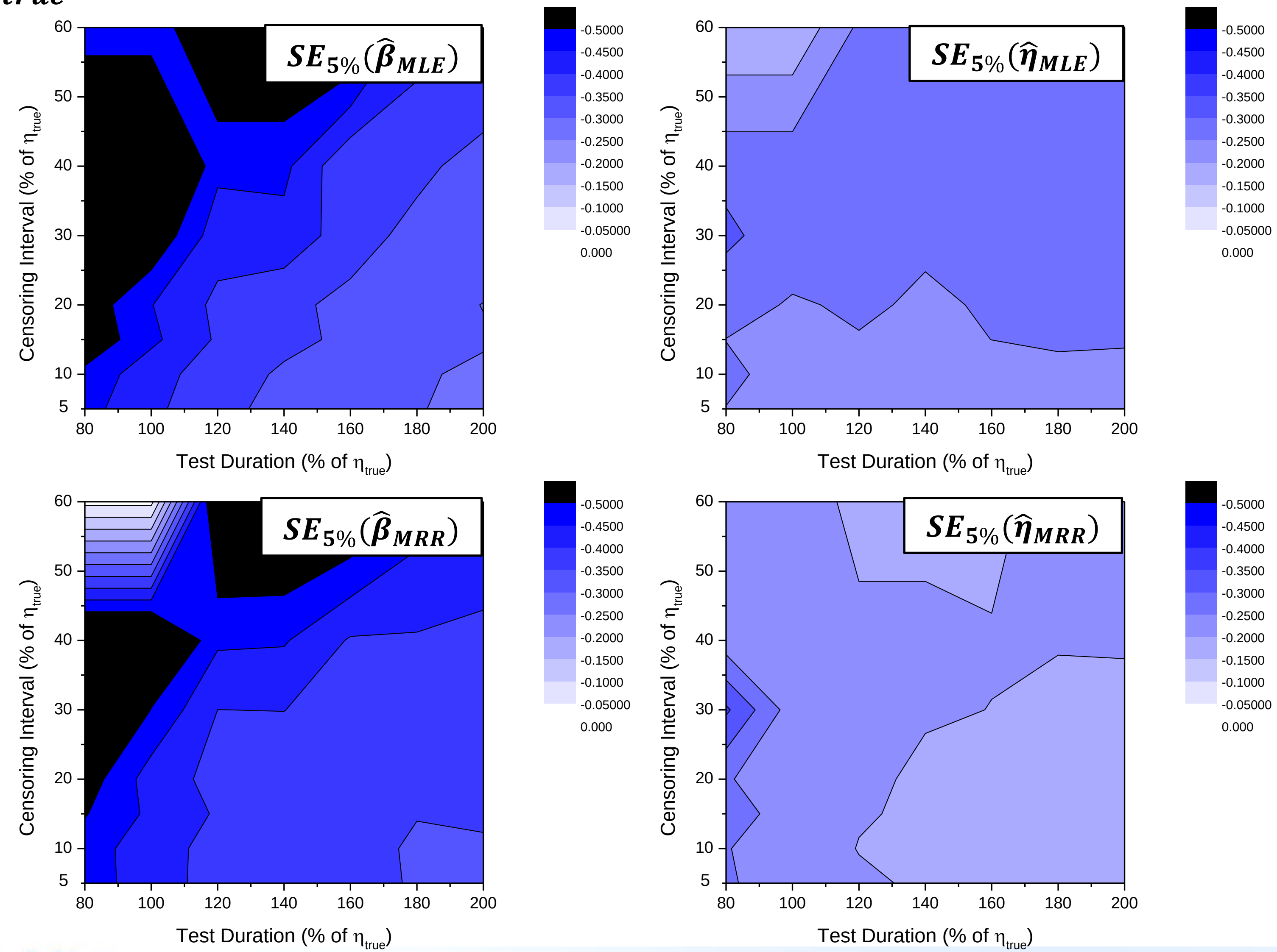

Fixed Specimen Number (10 ea.)

- $\beta_{true} = 3$

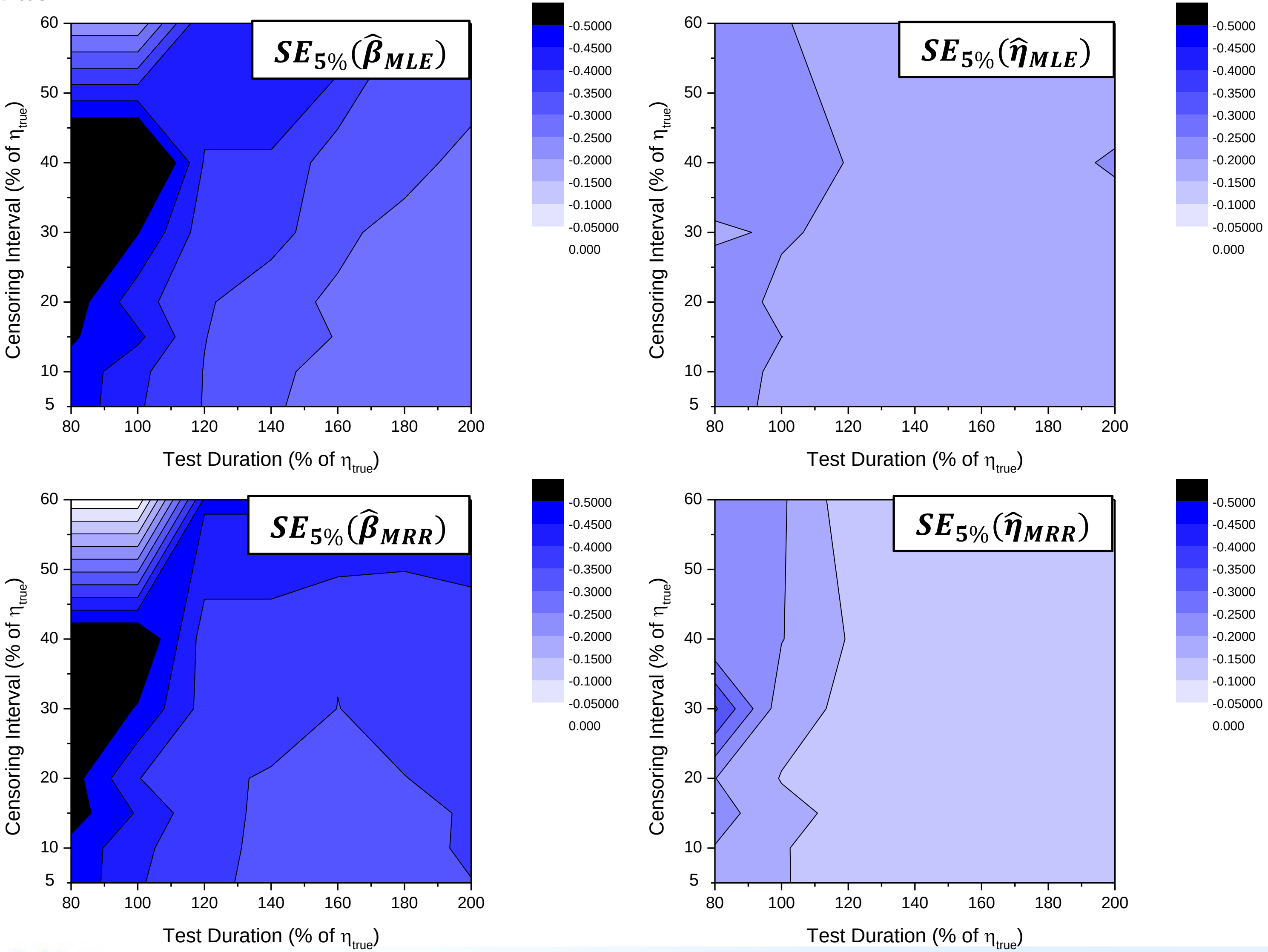

Fixed Specimen Number (10 ea.)

- $\beta_{true} = 4$

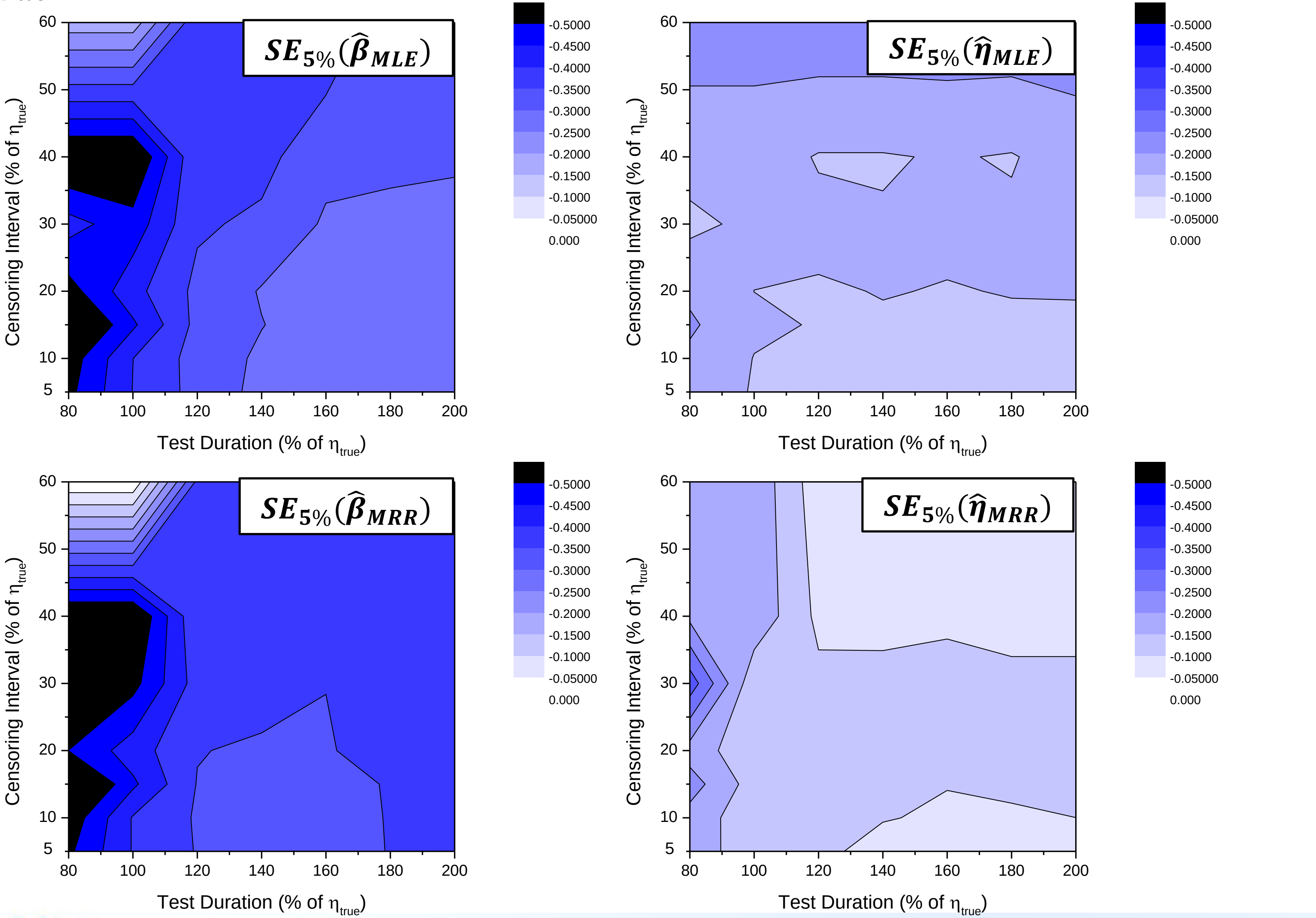

Fixed Specimen Number (10 ea.)

- $\beta_{true} = 2$

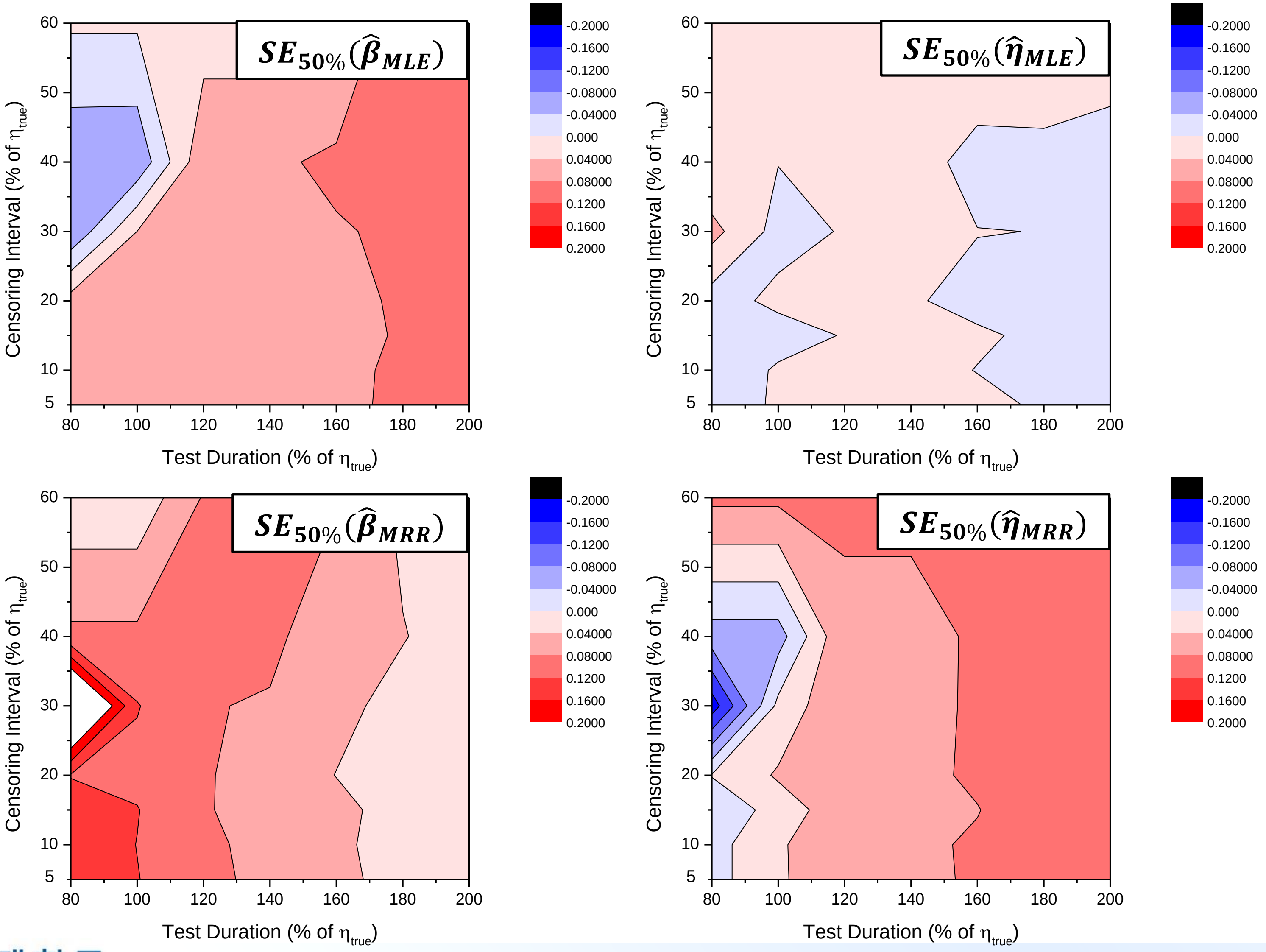

Fixed Specimen Number (10 ea.)

- $\beta_{true} = 3$

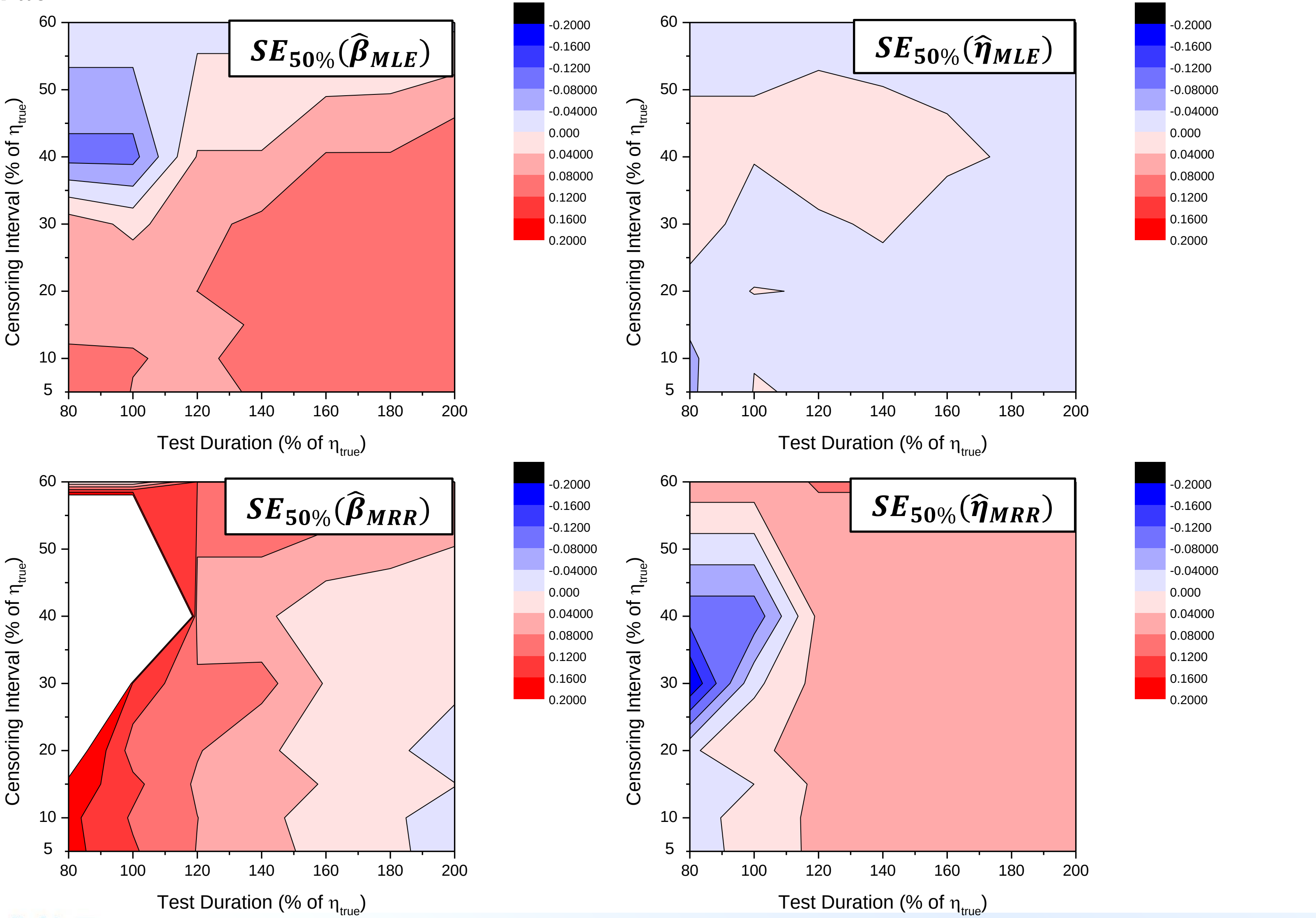

Fixed Specimen Number (10 ea.)

- $\beta_{true} = 4$

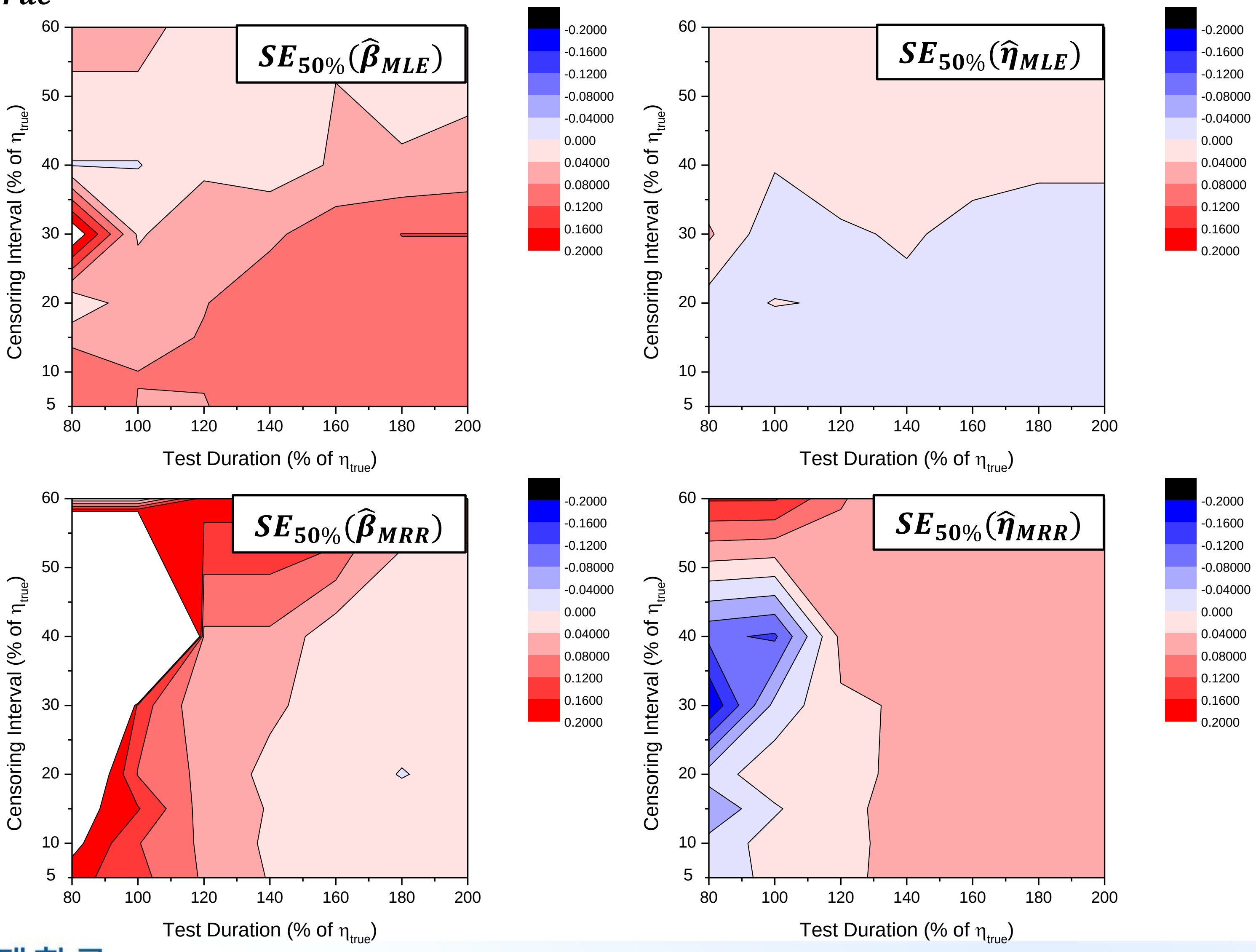

Fixed Specimen Number (10 ea.)

- $\beta_{true} = 2$

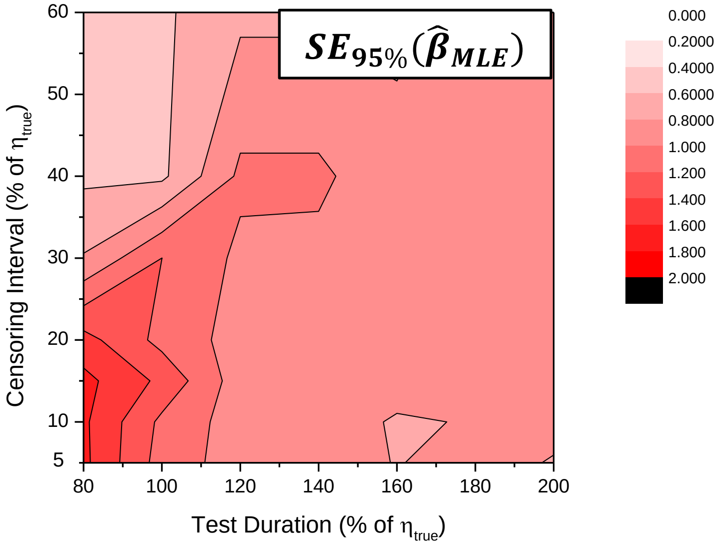

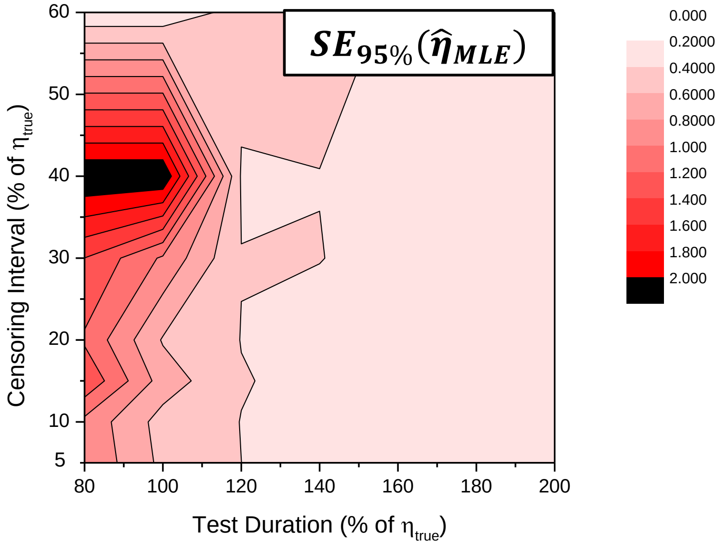

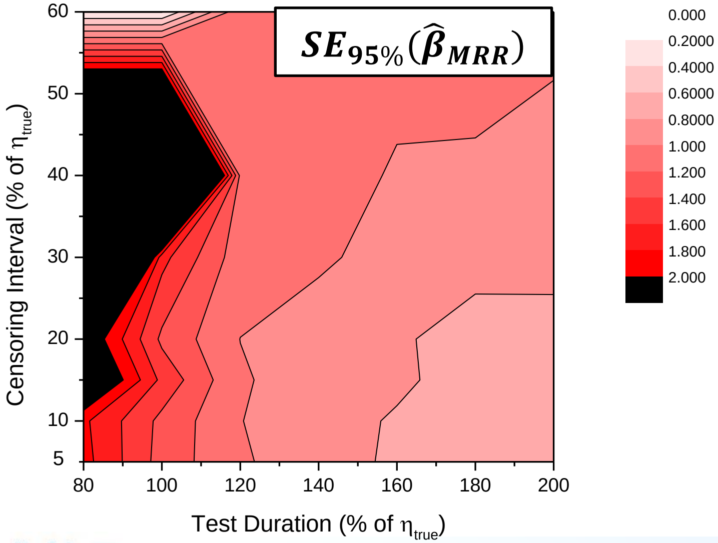

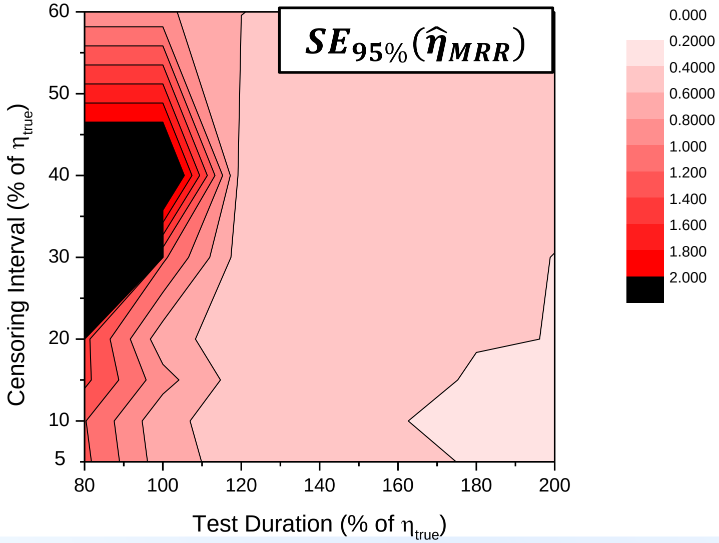

Fixed Specimen Number (10 ea.)

- $\beta_{true} = 3$

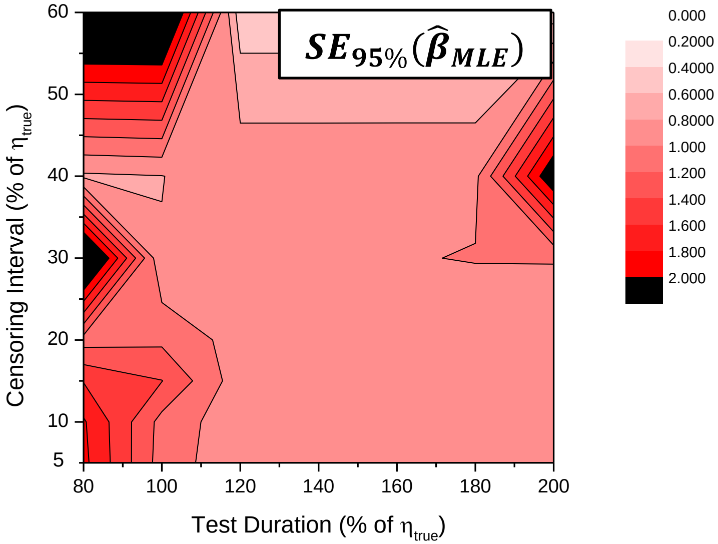

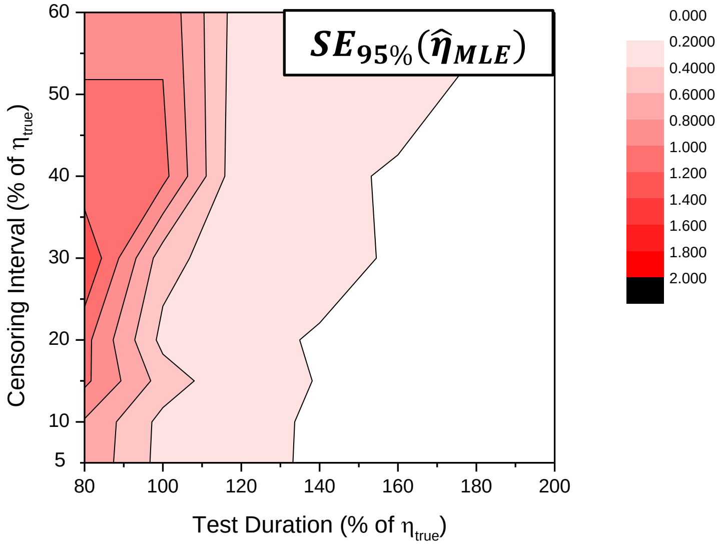

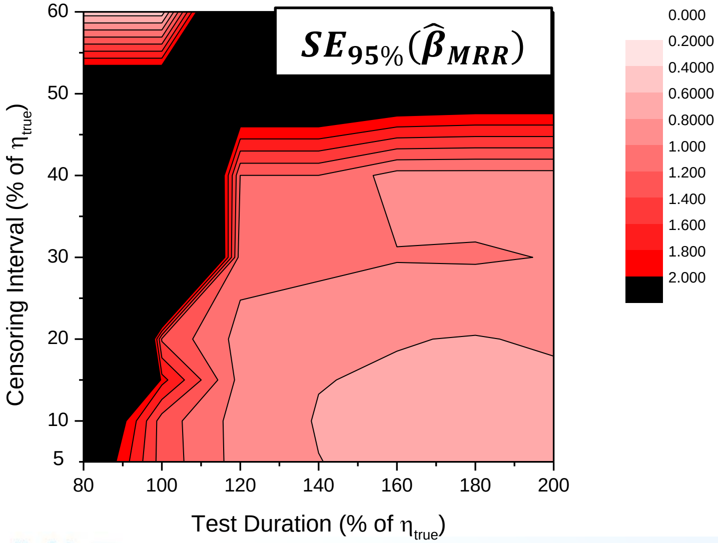

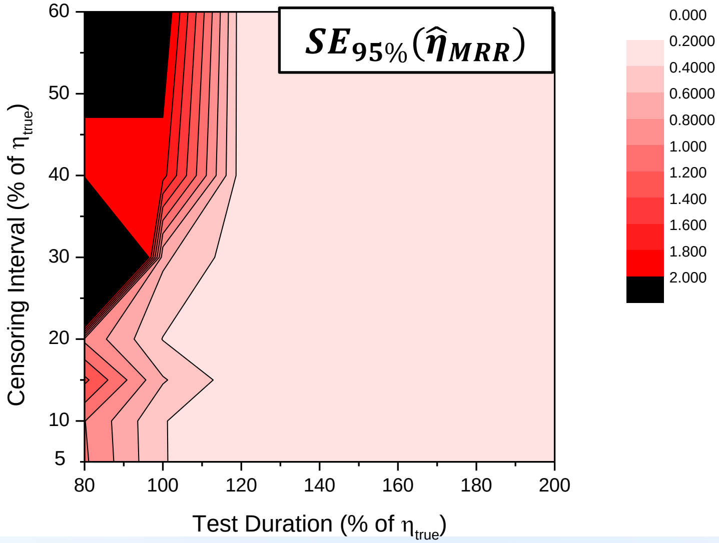

Fixed Specimen Number (10 ea.)

- $\beta_{true} = 4$

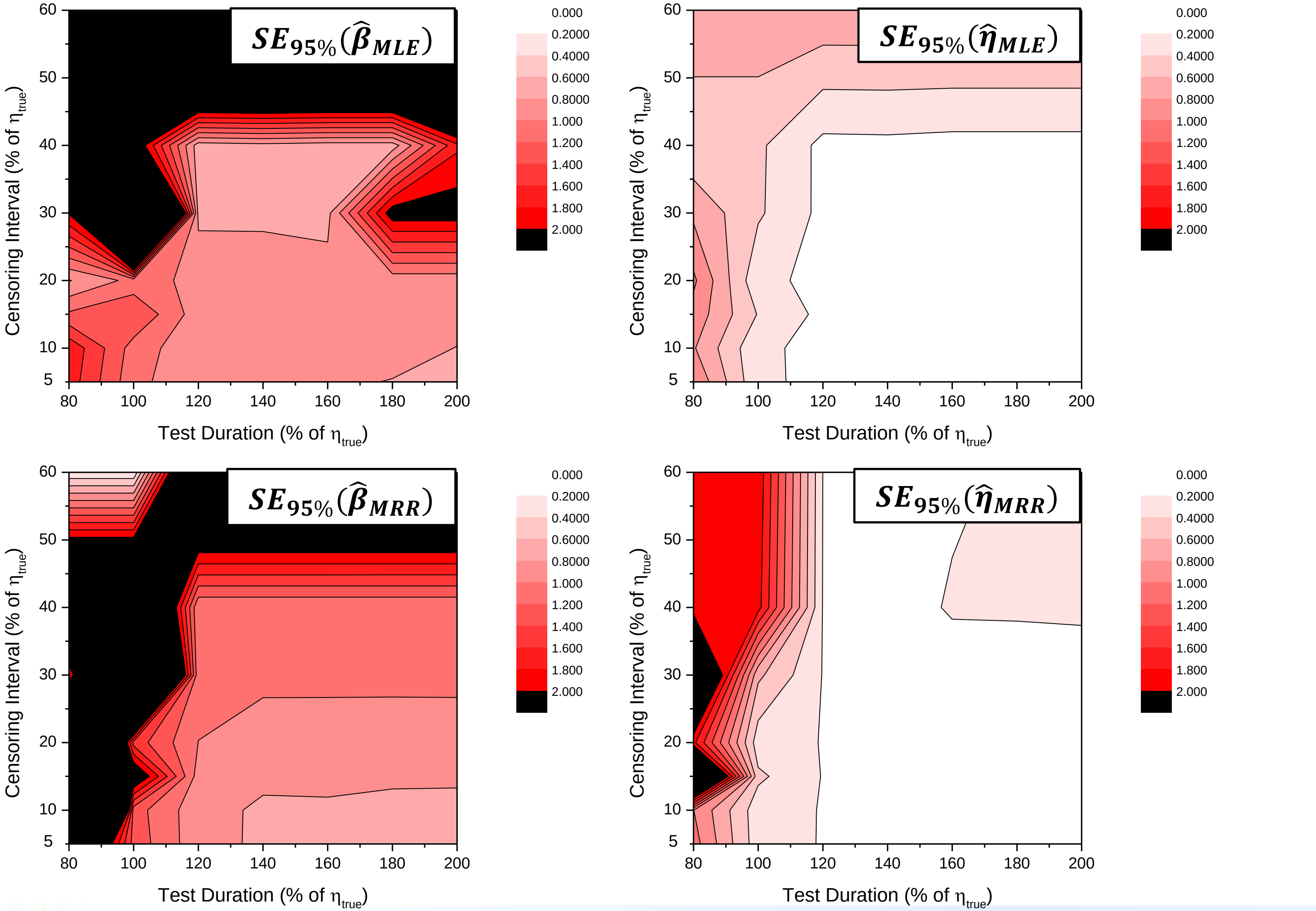

Fixed Specimen Number (10 ea.)

- $\beta_{true} = 2$

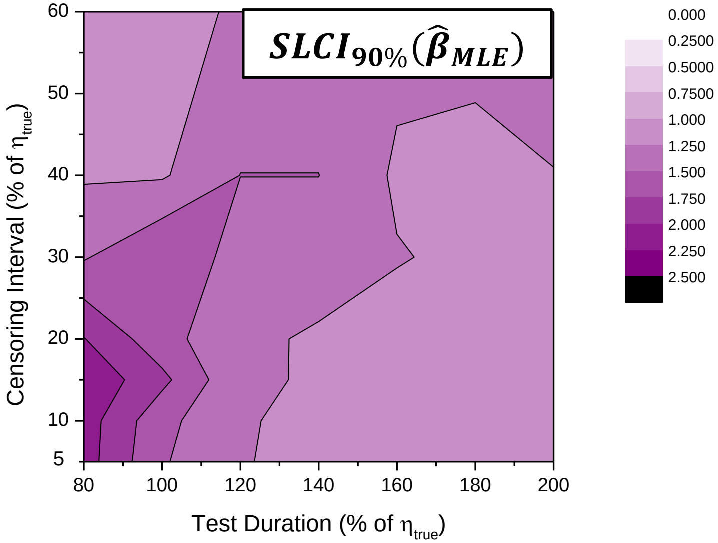

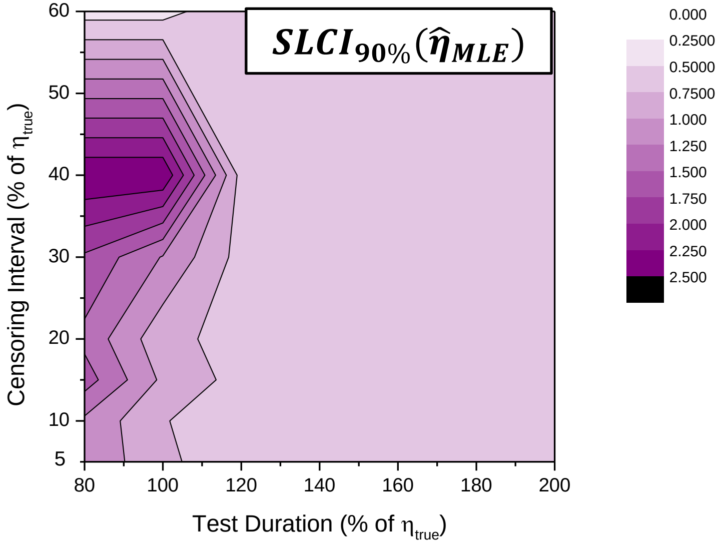

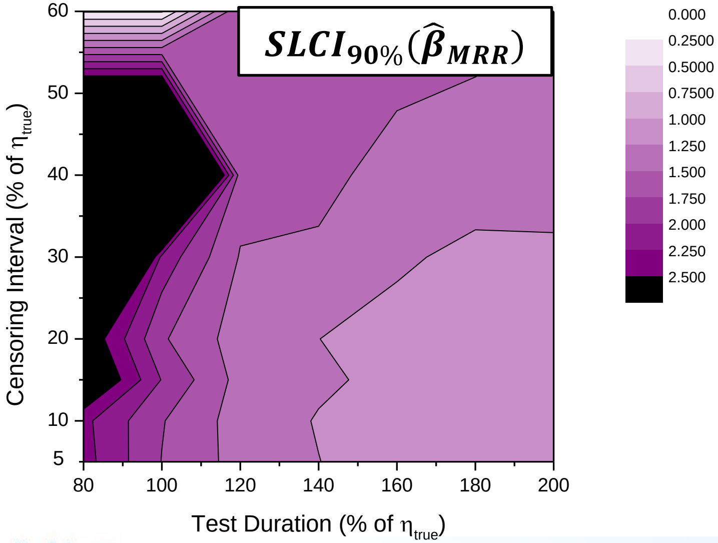

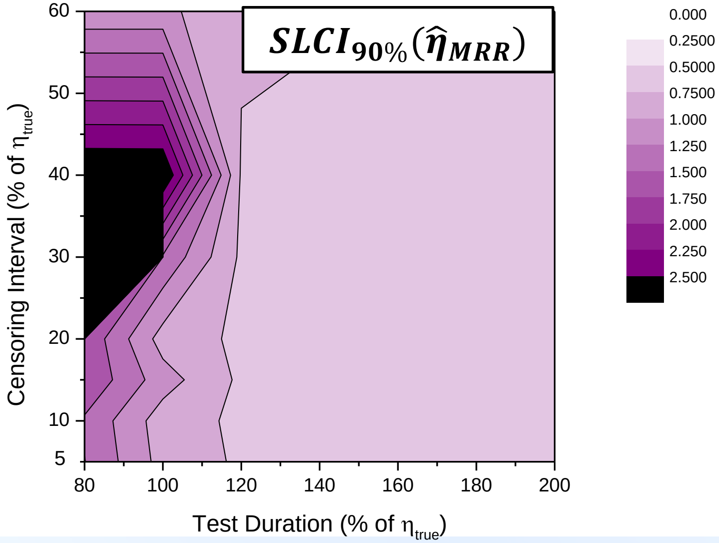

Fixed Specimen Number (10 ea.)

- $\beta_{true} = 3$

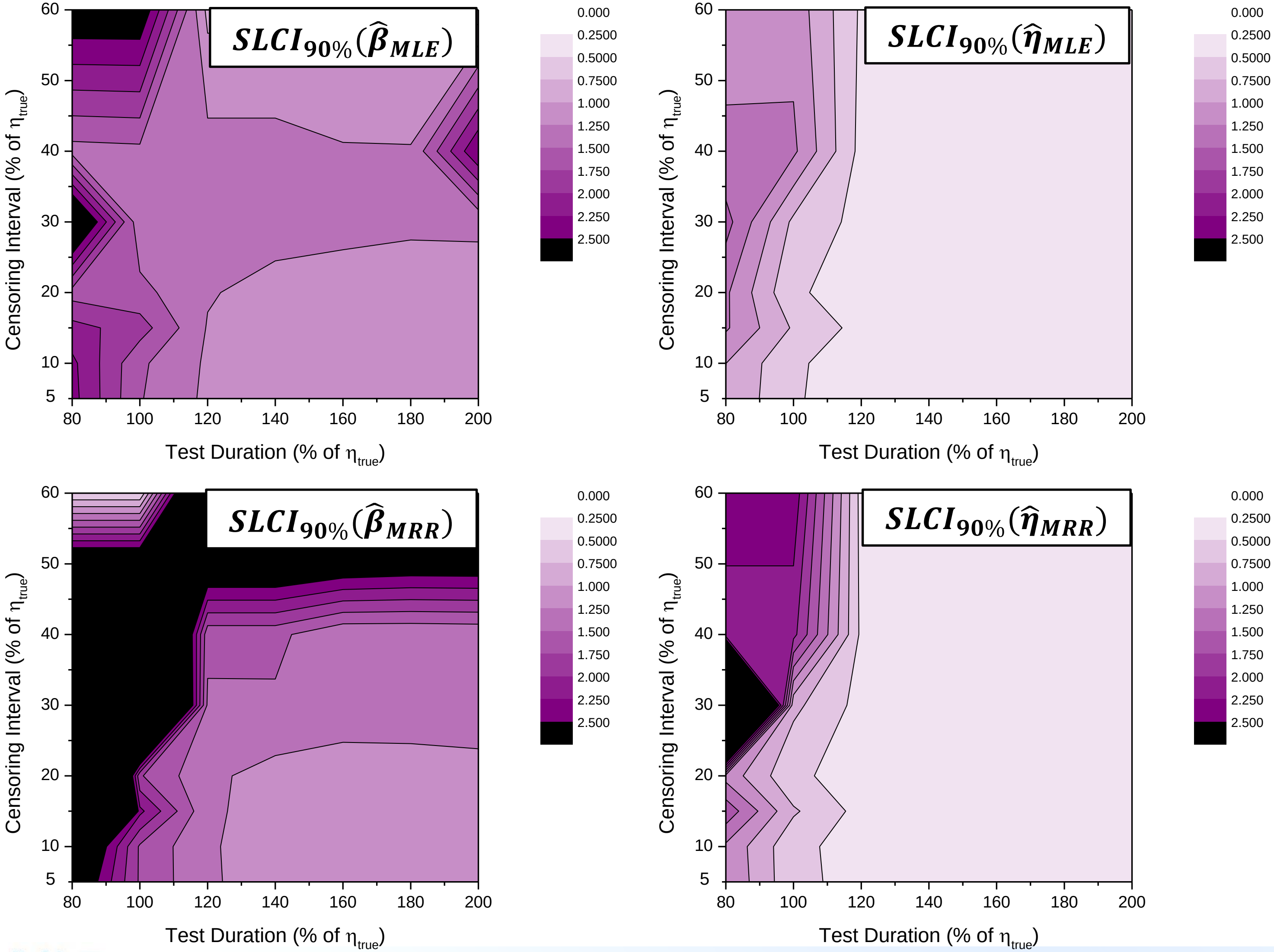

Fixed Specimen Number (10 ea.)

- $\beta_{true} = 4$

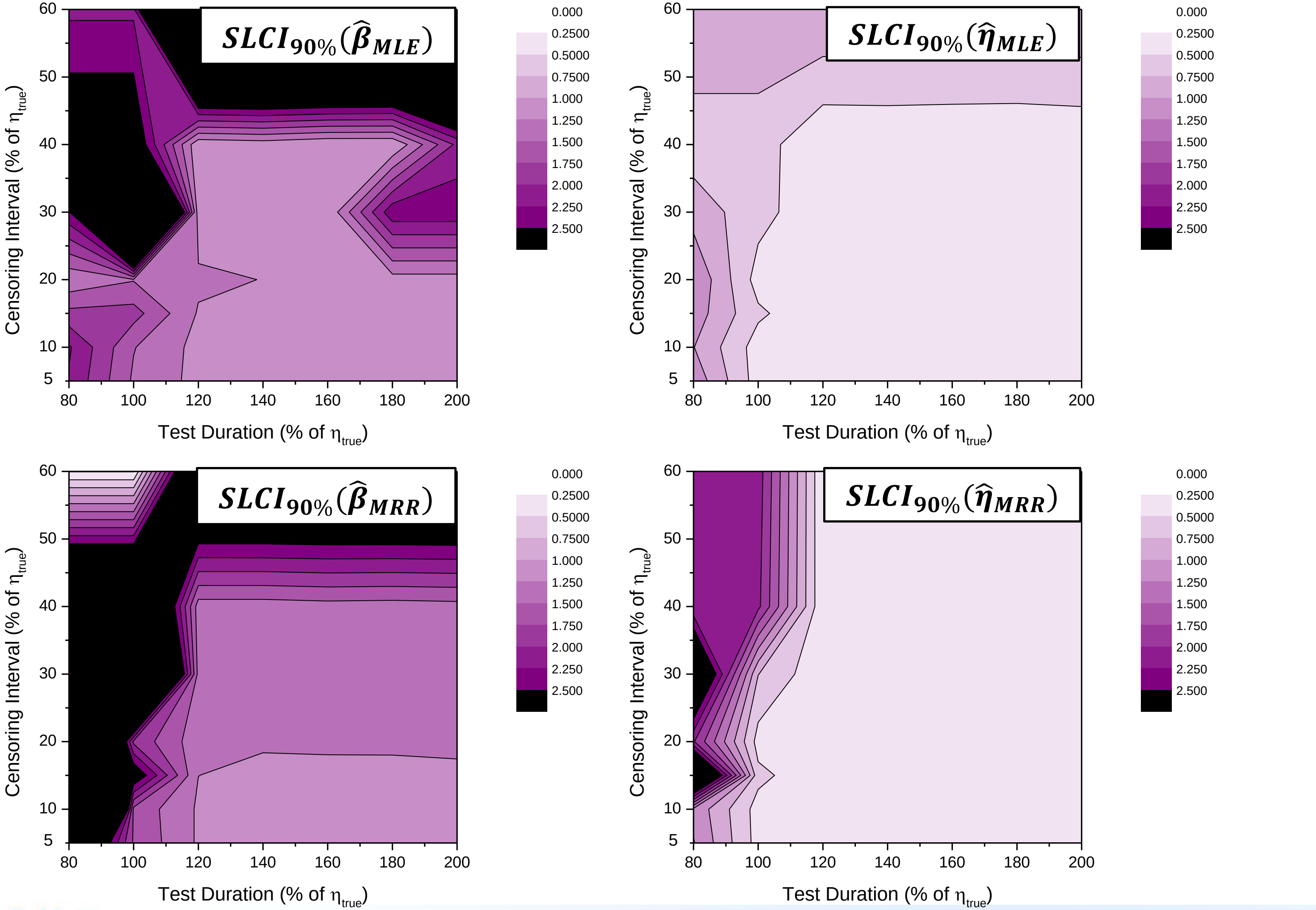

Supplement: Supplementary file 1 [file materials-09-00521-s001.zip › supp/All contour plots.pdf]
