# Supplementary material for: Uncertainty Evaluation of Weibull Estimators through Monte Carlo Simulation: Applications for Crack Initiation Testing
Source: Materials (Basel). 2016 Jun 27;9(7):521. doi: 10.3390/ma9070521 (PMC5456946; doi:10.3390/ma9070521)
Supplement: Supplementary file 1 [file materials-09-00521-s001.zip › supp/Derivation of interval censored ML simultaneous equation for 2-parameter Weibull distribution.docx]

**Supplementary Materials: Uncertainty Evaluation of Weibull Estimators through Monte Carlo Simulation: Applications for Crack Initiation Testing**

Jae Phil Park and Chi Bum Bahn

Derivation of Interval Censored ML Simultaneous Equation for 2-Parameter Weibull Distribution

1. Given Equations

CDF of the 2-parameter Weibull distribution:

| $F\left( t;\beta,\eta\right)=1-\exp\left[ -\left( \frac{t}{\eta} \right)^{\beta} \right]; t\geq0; \beta, \eta>0$ | (S1) |
| --- | --- |

Likelihood function for interval censored data:

| $L(\beta,\eta)= \prod_{i=1}^{S} [1-F(s_{i};\beta,\eta)]\cdot\prod_{j=1}^{C} [F\left( c_{j_{U}};\beta,\eta\right)-F(c_{j_{L}};\beta,\eta)]$ | (S2) |
| --- | --- |

Log-likelihood function for interval censored data:

| $\Lambda\left( \beta,\eta\right)=\ln L\left( \beta,\eta\right)$  $= \sum_{i=1}^{S} \ln[1-F\left( s_{i};\beta,\eta\right)]+ \sum_{j=1}^{C} \ln[F\left( c_{j_{U}};\beta,\eta\right)-F(c_{j_{L}};\beta,\eta)]$ | (S3) |
| --- | --- |

The condition of maximum likelihood point (i.e., ML simultaneous equation):

| $\left\{ \begin{aligned} \frac{\partial}{\partial\beta}\Lambda\left( \beta,\eta\right)=0 \\ \frac{\partial}{\partial\eta}\Lambda\left( \beta,\eta\right)=0 \end{aligned} \right.$ | (S4) |
| --- | --- |

2. Derivation

Substituting the Equation (3) into the Equation (4), we can get

| $\frac{\partial}{\partial\beta}\Lambda\left( \beta,\eta\right)=\frac{\partial}{\partial\beta}\left[ \sum_{i=1}^{S} \ln\left[ 1-F\left( s_{i};\beta,\eta\right) \right]+ \sum_{j=1}^{C} \ln\left[ F\left( c_{j_{U}};\beta,\eta\right)-F\left( c_{j_{L}};\beta,\eta\right) \right] \right]$  $= \sum_{i=1}^{S} \frac{\partial}{\partial\beta}\ln\left[ 1-F\left( s_{i};\beta,\eta\right) \right]+ \sum_{j=1}^{C} \frac{\partial}{\partial\beta}\ln\left[ F\left( c_{j_{U}};\beta,\eta\right)-F\left( c_{j_{L}};\beta,\eta\right) \right]$ | (S5) |
| --- | --- |

and

| $\frac{\partial}{\partial\eta}\Lambda\left( \beta,\eta\right)= \frac{\partial}{\partial\eta}\left[ \sum_{i=1}^{S} \ln\left[ 1-F\left( s_{i};\beta,\eta\right) \right]+ \sum_{j=1}^{C} \ln\left[ F\left( c_{j_{U}};\beta,\eta\right)-F\left( c_{j_{L}};\beta,\eta\right) \right] \right]$  $= \sum_{i=1}^{S} \frac{\partial}{\partial\eta}\ln\left[ 1-F\left( s_{i};\beta,\eta\right) \right]+ \sum_{j=1}^{C} \frac{\partial}{\partial\eta}\ln\left[ F\left( c_{j_{U}};\beta,\eta\right)-F\left( c_{j_{L}};\beta,\eta\right) \right].$ | (S6) |
| --- | --- |

The left term of the Equation (5) is

| $\sum_{i=1}^{S} \frac{\partial}{\partial\beta}\ln\left[ 1-F\left( s_{i};\beta,\eta\right) \right]= \sum_{i=1}^{S} \frac{\partial}{\partial\beta}\ln\left[ 1-\left[ 1-\exp\left[ -\left( \frac{s_{i}}{\eta} \right)^{\beta} \right] \right] \right]$  $= \sum_{i=1}^{S} \frac{\partial}{\partial\beta}\ln\left[ \exp\left[ -\left( \frac{s_{i}}{\eta} \right)^{\beta} \right] \right]$  $= \sum_{i=1}^{S} \frac{\partial}{\partial\beta}\left[ -\left( \frac{s_{i}}{\eta} \right)^{\beta} \right]$  $= \sum_{i=1}^{S} \left[ -\left( \frac{s_{i}}{\eta} \right)^{\beta}\ln\left( \frac{s_{i}}{\eta} \right) \right].$ | (S7) |
| --- | --- |

The right term of the Equation (5) is

| $\sum_{j=1}^{C} \frac{\partial}{\partial\beta}\ln\left[ F\left( c_{j_{U}};\beta,\eta\right)-F\left( c_{j_{L}};\beta,\eta\right) \right]$  $= \sum_{j=1}^{C} \frac{\partial}{\partial\beta}\ln\left[ \left[ 1-\exp\left[ -\left( \frac{c_{j_{U}}}{\eta} \right)^{\beta} \right] \right]-\left[ 1-\exp\left[ -\left( \frac{c_{j_{L}}}{\eta} \right)^{\beta} \right] \right] \right]$  $= \sum_{j=1}^{C} \frac{\partial}{\partial\beta}\ln\left[ \exp\left[ -\left( \frac{c_{j_{L}}}{\eta} \right)^{\beta} \right]-\exp\left[ -\left( \frac{c_{j_{U}}}{\eta} \right)^{\beta} \right] \right]$  $= \sum_{j=1}^{C} \frac{\frac{\partial}{\partial\beta}\left[ \exp\left[ -\left( \frac{c_{j_{L}}}{\eta} \right)^{\beta} \right]-\exp\left[ -\left( \frac{c_{j_{U}}}{\eta} \right)^{\beta} \right] \right]}{\exp\left[ -\left( \frac{c_{j_{L}}}{\eta} \right)^{\beta} \right]-\exp\left[ -\left( \frac{c_{j_{U}}}{\eta} \right)^{\beta} \right]}$  $= \sum_{j=1}^{C} \frac{\exp\left[ -\left( \frac{c_{j_{L}}}{\eta} \right)^{\beta} \right]\frac{\partial}{\partial\beta}\left[ -\left( \frac{c_{j_{L}}}{\eta} \right)^{\beta} \right]-\exp\left[ -\left( \frac{c_{j_{U}}}{\eta} \right)^{\beta} \right]\frac{\partial}{\partial\beta}\left[ -\left( \frac{c_{j_{U}}}{\eta} \right)^{\beta} \right]}{\exp\left[ -\left( \frac{c_{j_{L}}}{\eta} \right)^{\beta} \right]-\exp\left[ -\left( \frac{c_{j_{U}}}{\eta} \right)^{\beta} \right]}$  $= \sum_{j=1}^{C} \frac{\exp\left[ -\left( \frac{c_{j_{L}}}{\eta} \right)^{\beta} \right]\left[ -\left( \frac{c_{j_{L}}}{\eta} \right)^{\beta} \right]\ln\left( \frac{c_{j_{L}}}{\eta} \right)-\exp\left[ -\left( \frac{c_{j_{U}}}{\eta} \right)^{\beta} \right]\left[ -\left( \frac{c_{j_{U}}}{\eta} \right)^{\beta} \right]\ln\left( \frac{c_{j_{U}}}{\eta} \right)}{\exp\left[ -\left( \frac{c_{j_{L}}}{\eta} \right)^{\beta} \right]-\exp\left[ -\left( \frac{c_{j_{U}}}{\eta} \right)^{\beta} \right]}$  $= \sum_{j=1}^{C} \left[ \frac{-\left( \frac{c_{j_{L}}}{\eta} \right)^{\beta}\ln\left( \frac{c_{j_{L}}}{\eta} \right)\exp\left[ -\left( \frac{c_{j_{L}}}{\eta} \right)^{\beta} \right] + \left( \frac{c_{j_{U}}}{\eta} \right)^{\beta}\ln\left( \frac{c_{j_{U}}}{\eta} \right)\exp\left[ -\left( \frac{c_{j_{U}}}{\eta} \right)^{\beta} \right]}{\exp\left[ -\left( \frac{c_{j_{L}}}{\eta} \right)^{\beta} \right] - \exp\left[ -\left( \frac{c_{j_{U}}}{\eta} \right)^{\beta} \right]} \right].$ | (S8) |
| --- | --- |

The left term of the Equation (6) is

| $\sum_{i=1}^{S} \frac{\partial}{\partial\eta}\ln\left[ 1-F\left( s_{i};\beta,\eta\right) \right]= \sum_{i=1}^{S} \frac{\partial}{\partial\eta}\ln\left[ 1-\left[ 1-\exp\left[ -\left( \frac{s_{i}}{\eta} \right)^{\beta} \right] \right] \right]$  $= \sum_{i=1}^{S} \frac{\partial}{\partial\eta}\ln\left[ \exp\left[ -\left( \frac{s_{i}}{\eta} \right)^{\beta} \right] \right]$  $= \sum_{i=1}^{S} \frac{\partial}{\partial\eta}\left[ -\left( \frac{s_{i}}{\eta} \right)^{\beta} \right]$  $= \sum_{i=1}^{S} \frac{\partial}{\partial\eta}\left[ -\left( \frac{\eta}{s_{i}} \right)^{-\beta} \right]$  $= \sum_{i=1}^{S} \left( -\beta\right)\left[ -\left( \frac{\eta}{s_{i}} \right)^{-\beta-1} \right]\left( \frac{1}{s_{i}} \right)$  $= \sum_{i=1}^{S} \left( -\beta\right)\left[ -\left( \frac{s_{i}}{\eta} \right)^{\beta+1} \right]\left( \frac{1}{s_{i}} \right)$  $= \sum_{i=1}^{S} \left[ \left( \frac{\beta}{\eta} \right)\left( \frac{s_{i}}{\eta} \right)^{\beta} \right].$ | (S9) |
| --- | --- |

The right term of the Equation (6) is

| $\sum_{j=1}^{C} \frac{\partial}{\partial\eta}\ln\left[ F\left( c_{j_{U}};\beta,\eta\right)-F\left( c_{j_{L}};\beta,\eta\right) \right]$  $= \sum_{j=1}^{C} \frac{\partial}{\partial\eta}\ln\left[ \left[ 1-\exp\left[ -\left( \frac{c_{j_{U}}}{\eta} \right)^{\beta} \right] \right]-\left[ 1-\exp\left[ -\left( \frac{c_{j_{L}}}{\eta} \right)^{\beta} \right] \right] \right]$  $= \sum_{j=1}^{C} \frac{\partial}{\partial\eta}\ln\left[ \exp\left[ -\left( \frac{c_{j_{L}}}{\eta} \right)^{\beta} \right]-\exp\left[ -\left( \frac{c_{j_{U}}}{\eta} \right)^{\beta} \right] \right]$  $= \sum_{j=1}^{C} \frac{\frac{\partial}{\partial\eta}\left[ \exp\left[ -\left( \frac{c_{j_{L}}}{\eta} \right)^{\beta} \right]-\exp\left[ -\left( \frac{c_{j_{U}}}{\eta} \right)^{\beta} \right] \right]}{\exp\left[ -\left( \frac{c_{j_{L}}}{\eta} \right)^{\beta} \right]-\exp\left[ -\left( \frac{c_{j_{U}}}{\eta} \right)^{\beta} \right]}$  $= \sum_{j=1}^{C} \frac{\exp\left[ -\left( \frac{c_{j_{L}}}{\eta} \right)^{\beta} \right]\frac{\partial}{\partial\eta}\left[ -\left( \frac{c_{j_{L}}}{\eta} \right)^{\beta} \right]-\exp\left[ -\left( \frac{c_{j_{U}}}{\eta} \right)^{\beta} \right]\frac{\partial}{\partial\eta}\left[ -\left( \frac{c_{j_{U}}}{\eta} \right)^{\beta} \right]}{\exp\left[ -\left( \frac{c_{j_{L}}}{\eta} \right)^{\beta} \right]-\exp\left[ -\left( \frac{c_{j_{U}}}{\eta} \right)^{\beta} \right]}$  $= \sum_{j=1}^{C} \frac{\exp\left[ -\left( \frac{c_{j_{L}}}{\eta} \right)^{\beta} \right]\frac{\partial}{\partial\eta}\left[ -\left( \frac{\eta}{c_{j_{L}}} \right)^{-\beta} \right]-\exp\left[ -\left( \frac{c_{j_{U}}}{\eta} \right)^{\beta} \right]\frac{\partial}{\partial\eta}\left[ -\left( \frac{\eta}{c_{j_{U}}} \right)^{-\beta} \right]}{\exp\left[ -\left( \frac{c_{j_{L}}}{\eta} \right)^{\beta} \right]-\exp\left[ -\left( \frac{c_{j_{U}}}{\eta} \right)^{\beta} \right]}$  $= \sum_{j=1}^{C} \frac{\exp\left[ -\left( \frac{c_{j_{L}}}{\eta} \right)^{\beta} \right]\left[ \left( -\beta\right)\left[ -\left( \frac{\eta}{c_{j_{L}}} \right)^{-\beta-1} \right]\left( \frac{1}{c_{j_{L}}} \right) \right]-\exp\left[ -\left( \frac{c_{j_{U}}}{\eta} \right)^{\beta} \right]\left[ \left( -\beta\right)\left[ -\left( \frac{\eta}{c_{j_{U}}} \right)^{-\beta-1} \right]\left( \frac{1}{c_{j_{U}}} \right) \right]}{\exp\left[ -\left( \frac{c_{j_{L}}}{\eta} \right)^{\beta} \right]-\exp\left[ -\left( \frac{c_{j_{U}}}{\eta} \right)^{\beta} \right]}$  $= \sum_{j=1}^{C} \frac{\exp\left[ -\left( \frac{c_{j_{L}}}{\eta} \right)^{\beta} \right]\left[ \left( -\beta\right)\left[ -\left( \frac{c_{j_{L}}}{\eta} \right)^{\beta+1} \right]\left( \frac{1}{c_{j_{L}}} \right) \right]-\exp\left[ -\left( \frac{c_{j_{U}}}{\eta} \right)^{\beta} \right]\left[ \left( -\beta\right)\left[ -\left( \frac{c_{j_{U}}}{\eta} \right)^{\beta+1} \right]\left( \frac{1}{c_{j_{U}}} \right) \right]}{\exp\left[ -\left( \frac{c_{j_{L}}}{\eta} \right)^{\beta} \right]-\exp\left[ -\left( \frac{c_{j_{U}}}{\eta} \right)^{\beta} \right]}$  $= \sum_{j=1}^{C} \frac{\exp\left[ -\left( \frac{c_{j_{L}}}{\eta} \right)^{\beta} \right]\left[ \left( \frac{\beta}{\eta} \right)\left( \frac{c_{j_{L}}}{\eta} \right)^{\beta} \right]-\exp\left[ -\left( \frac{c_{j_{U}}}{\eta} \right)^{\beta} \right]\left[ \left( \frac{\beta}{\eta} \right)\left( \frac{c_{j_{U}}}{\eta} \right)^{\beta} \right]}{\exp\left[ -\left( \frac{c_{j_{L}}}{\eta} \right)^{\beta} \right]-\exp\left[ -\left( \frac{c_{j_{U}}}{\eta} \right)^{\beta} \right]}$  $= \sum_{j=1}^{C} \left[ \left( \frac{\beta}{\eta} \right)\frac{\left( \frac{c_{j_{L}}}{\eta} \right)^{\beta}\exp\left[ -\left( \frac{c_{j_{L}}}{\eta} \right)^{\beta} \right] - \left( \frac{c_{j_{U}}}{\eta} \right)^{\beta}\exp\left[ -\left( \frac{c_{j_{U}}}{\eta} \right)^{\beta} \right]}{\exp\left[ -\left( \frac{c_{j_{L}}}{\eta} \right)^{\beta} \right] - \exp\left[ -\left( \frac{c_{j_{U}}}{\eta} \right)^{\beta} \right]} \right].$ | (S10) |
| --- | --- |

Therefore, the final form of the interval censored ML simultaneous equation is

| $\left\{ \begin{aligned} \sum_{i=1}^{S} \left[ -\left( \frac{s_{i}}{\eta} \right)^{\beta}\ln\left( \frac{s_{i}}{\eta} \right) \right]+\sum_{j=1}^{C} \left[ \frac{-\left( \frac{c_{j_{L}}}{\eta} \right)^{\beta}\ln\left( \frac{c_{j_{L}}}{\eta} \right)\exp\left[ -\left( \frac{c_{j_{L}}}{\eta} \right)^{\beta} \right] + \left( \frac{c_{j_{U}}}{\eta} \right)^{\beta}\ln\left( \frac{c_{j_{U}}}{\eta} \right)\exp\left[ -\left( \frac{c_{j_{U}}}{\eta} \right)^{\beta} \right]}{\exp\left[ -\left( \frac{c_{j_{L}}}{\eta} \right)^{\beta} \right] - \exp\left[ -\left( \frac{c_{j_{U}}}{\eta} \right)^{\beta} \right]} \right]=0 \\ \sum_{i=1}^{S} \left[ \left( \frac{\beta}{\eta} \right)\left( \frac{s_{i}}{\eta} \right)^{\beta} \right]+ \sum_{j=1}^{C} \left[ \left( \frac{\beta}{\eta} \right)\frac{\left( \frac{c_{j_{L}}}{\eta} \right)^{\beta}\exp\left[ -\left( \frac{c_{j_{L}}}{\eta} \right)^{\beta} \right] - \left( \frac{c_{j_{U}}}{\eta} \right)^{\beta}\exp\left[ -\left( \frac{c_{j_{U}}}{\eta} \right)^{\beta} \right]}{\exp\left[ -\left( \frac{c_{j_{L}}}{\eta} \right)^{\beta} \right] - \exp\left[ -\left( \frac{c_{j_{U}}}{\eta} \right)^{\beta} \right]} \right]=0 \end{aligned}. \right.$ | (S11) |
| --- | --- |
